# The Effects of (Dis)similarities Between the Creator and the Assessor on Assessing Creativity: A Comparison of Humans and LLMs

**DOI:** 10.3390/jintelligence13070080

**Published:** 2025-07-03

**Authors:** Martin op ‘t Hof, Ke Hu, Song Tong, Honghong Bai

**Affiliations:** 1 School of Articifical Intelligence, Radboud University, 6500 HE Nijmegen, The Netherlands; martin.opthof@ru.nl; 2 Department of Psychological and Cognitive Sciences, Tsinghua University, Beijing 100084, China; kellykehu314@gmail.com; 3 Department of Psychology, Faculty of Arts and Sciences, Beijing Normal University at Zhuhai, Zhuhai 519087, China; 4 Beijing Key Laboratory of Applied Experimental Psychology, National Demonstration Center for Experimental Psychology Education, Faculty of Psychology, Beijing Normal University, Beijing 100875, China; 5 Behavioural Science Institute & Orthopedagogics: Learning and Development, Radboud University, 6500 HE Nijmegen, The Netherlands

**Keywords:** creativity assessment, large language models, cross-cultural comparison

## Abstract

Current research predominantly involves human subjects to evaluate AI creativity. In this explorative study, we questioned the validity of this practice and examined how creator–assessor (dis)similarity—namely to what extent the creator and the assessor were alike—along two dimensions of culture (Western and English-speaking vs. Eastern and Chinese-speaking) and agency (human vs. AI) influences the assessment of creativity. We first asked four types of subjects to create stories, including Eastern participants (university students from China), Eastern AI (Kimi from China), Western participants (university students from The Netherlands), and Western AI (ChatGPT 3.5 from the US). Both Eastern participants and AI created stories in Chinese, which were then translated into English, while both Western participants and AI created stories in English, which were then translated into Chinese. A subset of these stories (2 creative and 2 uncreative per creator type, in total 16 stories) was then randomly selected as assessment materials. Adopting a within-subject design, we then asked new subjects from the same four types (*n* = 120, 30 per type) to assess these stories on creativity, originality, and appropriateness. The results confirmed that similarities in both dimensions of culture and agency influence the assessment of originality and appropriateness. As for the agency dimension, human assessors preferred human-created stories for originality, while AI assessors showed no preference. Conversely, AI assessors rated AI-generated stories higher in appropriateness, whereas human assessors showed no preference. Culturally, both Eastern and Western assessors favored Eastern-created stories in originality. And as for appropriateness, the assessors always preferred stories from the creators with the same cultural backgrounds. The present study is significant in attempting to ask an often-overlooked question and provides the first empirical evidence to underscore the need for more discussion on using humans to judge AI agents’ creativity or the other way around.

## 1. Introduction

Generative artificial intelligence (GenAI) is an emerging field in artificial intelligence research that focuses on AI models such as ChatGPT, which are capable of, when prompted by human users, generating outputs that are seemingly ‘original’ and appropriate to humans (e.g., a story). This conforms to the standard definition of creativity that is frequently adopted in human studies, where creative ideas are defined by meeting two criteria: being original and appropriate (Runco and Jaeger 2012). In this regard, focusing primarily on the outcomes, an assertive voice has been raised in public space as well as in academia that AI can be considered creative and, in some tasks, may approach or even match the average level of human creativity (Koivisto and Grassini 2023; Wang et al. 2024). Meanwhile, a small number of researchers do hold doubts or even an opposing proposition towards this voice, especially questioning whether AI can be considered creative if we shift attention to the process of ‘AI-creation’ (Runco 2023).

In addition to theoretical debates, an increasing number of researchers have also attempted to empirically examine whether GenAI models are indeed creative by involving human subjects to evaluate the works produced by these models (e.g., Maltese et al. 2024; Guzik et al. 2023). A major limitation of this approach points to its human-centered nature, regardless of the prominent species gap between the creator (i.e., machine) and the assessor (i.e., human). As underscored by Arendt (2013), while no two people are interchangeable given their unique, diverse backgrounds and views, all human beings belong to the same species and are sufficiently alike to understand each other (Tömmel and d’Entreves 2024). It is exactly this ‘sufficient alikeness’ or, in other words, a certain level of commonality or similarity between humans that enables one to assess another’s actions. Empirical evidence has also shown, when the alikeness compromises, such as the creator and the assessor are from different cultural backgrounds, that the assessment of creativity could be biased (e.g., Hempel and Sue-Chan 2010). Given that the alikeness between humans and AI is evidently questionable, this paper aimed to initiate a discussion on the validity of judging AI’s creativity by humans and reported on findings of an explorative study on how creator–assessor (dis)similarity—to what extent the creator and the assessor were alike—along two dimensions of culture (Western and English-speaking vs. Eastern and Chinese-speaking) and agency (human vs. AI) influences the assessment of creativity, comparing humans with LLMs with a within-subject experimental design.

### 1.1. Current Research on Assessing GenAI’s Creativity

Two major approaches are present in assessing the creativity of AI models in the current research. One line of research, especially those pertaining to art creation, often adopts Turing-test-like paradigms wherein human subjects are asked to distinguish human products from AI products and/or compare them (Chamberlain et al. 2018; Gangadharbatla 2022; Samo and Highhouse 2023) or simply evaluate whether AI products satisfy humans’ needs or standards (e.g., Elgammal et al. 2017). Another line of research, especially when LLM-based models such as ChatGPT were involved, adopted the psychological approach of evaluating AI models’ creativity based on how well they perform on tasks that were designed to evaluate humans’ creativity (Guzik et al. 2023; Maltese et al. 2024). For instance, Cropley (2023) compared ChatGPT’s and human subjects’ performance on a verbal divergent thinking task—the Divergent Association Task—wherein subjects were asked to name ‘10 words that are as different from each other as possible, in all meanings and uses of the words’. They found that ChatGPT even outperformed humans in this task.

The limitation with either of the aforementioned approaches points to their human-centered nature, which sets the estimation of AI creativity to appeal to humans’ interests and in the context of human knowledge. This may indeed serve the purpose of developing AI agents to substitute humans, if desired and ever feasible (see for emerging counter evidence Yiu et al. 2023). It overshadows the possibility that AI may possess a different type or level of creativity based on its own ‘knowledge’, analogous to that children show a different kind of creativity from adults. Moreover, empirical research revealed that involving human subjects as raters to assess AI creativity can lead to biases. For instance, Magni et al. (2024) found that people generally (though not always) ascribe lower creativity to a product when they are told that it is created by an AI rather than a human (similar findings in Gangadharbatla 2022; Grassini and Koivisto 2024; Hong 2018; Horton et al. 2023; Zhou and Kawabata 2023; c.f. Chamberlain et al. 2018), which was likely caused by the fact that people consistently perceive AI to exert less effort than humans in creation. An important takeaway from these findings points to the importance of ensuring a certain level of similarity between the creator and the assessor, for only then can the assessor remove their ‘prejudice’ towards the creator and fairly judge whether an idea or a product is creative.

### 1.2. Vice Versa: Using (Gen)AI to Assess Humans’ Creativity

Another recent development on (Gen)AI and creativity is that researchers, to leverage the rating efficiency and reduce rating subjectivity, increasingly use AI models to replace human raters in assessing human creations. Cropley and Marrone (2022) trained a convolutional neural network (CNN) to automate the scoring of the Test of Creative Thinking–Drawing Production—a figure task wherein people are asked to create drawings on the basis of a few given abstract figures (e.g., a line, a dot…). They reported that the scoring accuracy of this model was comparable to human raters, with inter-rater reliability between its scoring and human raters’ scoring, measured with Kappa, ranging from 0.827 to 1 for models with different granularity. In other studies, computational models of semantic distance (i.e., to what extent verbal elements are semantically related to each other) were applied to assess people’s responses to a mathematical creativity task (Marrone et al. 2023) or to divergent thinking tasks (the Divergent Association Task in Olson et al. 2021; and the Alternative Uses Task in Beaty and Johnson 2021). Recent work demonstrates that fine-tuned large language models (LLMs) can substantially outperform traditional semantic distance methods, achieving much higher correspondence with human ratings (Organisciak et al. 2023). These studies generally reported moderate to high positive correlations between the scoring of the models and the scoring of the human raters.

Contrasting our argument, which emphasizes the importance of ensuring the similarity between the creator and the assessor, this new development actually introduced more dissimilarity between the assessor and the creator, which may also lead to biases. For instance, the studies by Beaty and Johnson (2021) and Olson et al. (2021) used models that are trained on common, non-specific crawl corpus, including billions of words to form the referencing basis upon which the semantic distance of words appearing in creative responses can be calculated as a proxy of creativity. One could argue, in this case, that the broader context of the training data does not resemble the specific context of the testing data embedded in creative tasks, hence impairing the validity of the testing results. This context mismatch was addressed in Marrone et al. (2023), where the training data actually consisted of students’ responses. Yet, as noted by the authors themselves, the included students’ responses for the training were typically rated as low to medium levels of creativity by the human judges. In this regard, the trained AI model might only be able to capture and differentiate responses that fall within the low to middle range of creativity, but not the high end of creativity. Future research is warranted to take a closer look at the potential biases while applying AI models to assess humans’ creativity.

### 1.3. Cultural (Dis)similarity Affects People’s Assessment of Creativity

Another source of support for our inquiry into the effect of creator–assessor similarity on the assessment of creativity points to past research about cultural differences in humans’ creativity. Adopting the broad framework of distinguishing Western and Eastern cultures, past research often reported that Western individuals outperform eastern individuals on creative tasks (Ivancovsky et al. 2018; Liou and Lan 2018; Niu et al. 2007; Niu and Sternberg 2001; Wong and Niu 2013; Yi et al. 2013), though some studies that used tests in a figural form (Huntsinger et al. 2011; Storme et al. 2017) or which highly rely on science-based knowledge did report contradictory findings (Niu and Sternberg 2002). While a major conjecture points to the fact that the collective nature of Eastern cultures have hindered people’s expression of creativity (‘not to show’) more than their counterparts with a more individualistic cultural background, empirical evidence has also shown that different cultures lead people to think of and assess creativity in very different ways (Niu and Kaufman 2013). Specifically, in Eastern cultures (e.g., Chinese), emphasis is placed on appropriateness and the acceptance of ideas by the social group and the broader social environment. In contrast, Western cultures (e.g., American) prioritize originality, taking a more individualistic perspective in which creativity is associated with individuals who break free from ingrained norms and customs to do something in a completely original and unforeseen way. In this way, the assessment is questionable when the assessor and the creator have different cultural backgrounds.

### 1.4. LLMs vs. Humans: Two Prominent Sources of Dissimilarities

Between LLMs and humans, there are dissimilarities along two dimensions: agency and culture. *Agentic dissimilarity* refers to the species gap between LLMs and humans, one of non-organism and another of organism existence, which may influence how one perceives and reacts to the environment. Specifically, LLMs are computer algorithms that employ neural network architectures with up to billions of parameters and are trained on massive datasets of web-based, human-generated text. Based on the seminal work of Vaswani et al. (2017), such algorithms attend to both immediate and long-range dependencies and are hence powerful in learning patterns and structures in the text. Through self-supervised learning or reinforcement learning involving human feedback, these models are then trained on predicting the next most likely reasonable and meaningful tokens in a given sequence of text (Webb et al. 2023). Thus, when given a prompt from the users that provides language cues on the context, such as the story style and core story elements, LLMs can use these cues to search for closely related text fragments and fit them into one whole piece by their learned patterns and structures. This leads to creating a story that seems to be ‘new’, not identical to their training data.

In contrast to LLMs, the human mind works in a much more complex manner. And even though decades of research have been dedicated to this area, it is far from being fully understood by science today. An often-perceived unique trait of the human mind points to that it relies on logical reasoning for gaining knowledge. Yet, empirical research has shown that humans, more often than we are aware of, are actually poor logical reasoners (e.g., Evans 2002). Moreover, it gradually becomes clear in more recent research that human learning also takes place based on the regularity of received inputs (Dai et al. 2019; Romberg and Saffran 2010), similar to that of LLMs.

Note also that emerging evidence has shown that some LLMs (e.g., GPT-4) can balance between logical computation and heuristic reasoning when handling complex tasks, optimizing for both accuracy and computational efficiency (Mukherjee and Chang 2024). To some extent, this pattern aligns with the principles of resource-rational cognition in humans and corresponds to certain cognitive models proposed in bounded rationality and dual-process theory (Mukherjee and Chang 2024). Yet, this is far from indicating that LLMs and humans process information identically. LLMs operate through pattern recognition and probabilistic computation based on data, whereas human cognition incorporates causal inference, contextual integration, and, importantly, learning based on embodied experiences. Further differences are also embedded in optimization goals, processing constraints, and adaptive capacity, all of which can lead to distinct outcomes.

Finally, the agentic dissimilarity between LLMs and humans is likely also determined by their different access to languages. LLMs are often trained on text written in a variety of languages and are supposed to be able to interact with users using any desired language. Thus, LLMs might have a broader linguistic base than humans, who are typically proficient in only one or a few languages. For the same reason, LLMs might also be more proficient in cognitive flexibility as well as in navigating diverse conceptual frameworks (Rathje et al. 2024). However, having been aligned with human feedback, regional dialects, idiomatic expressions, and unique cultural elements may be flattened or lost as the model generalizes to serve a broader range of audiences and conform to mainstream aesthetics and values dominated by certain languages (Agarwal et al. 2025; Tao et al. 2024). Collectively, LLMs might have narrower access to unique, non-mainstream varieties compared to humans, as a trade-off for their broader access to different languages and their bounded knowledge.

*Cultural dissimilarity* refers to the potential cultural gap between LLMs and humans. As reported by some studies, current LLMs respond better towards the dominant cultures as embedded in their training data (e.g., ChatGPT mainly reflects English-based cultural norms and perspectives). For instance, Tao et al. (2024) analyzed the cultural biases in large language models (LLMs) such as GPT-4 by comparing their responses with nationally representative survey data. The study found that these models exhibit cultural values resembling those of English-speaking and Protestant European countries, potentially leading to a homogenization of cultural expression. In this regard, just like humans, LLMs might also possess a dominant ‘cultural background’ inherited from the dominant data stream while being trained. Hence, there might be a cultural gap between LLMs and humans when they come from different countries and/or have different dominant language bases.

### 1.5. The Present Study

**Figure 1:**
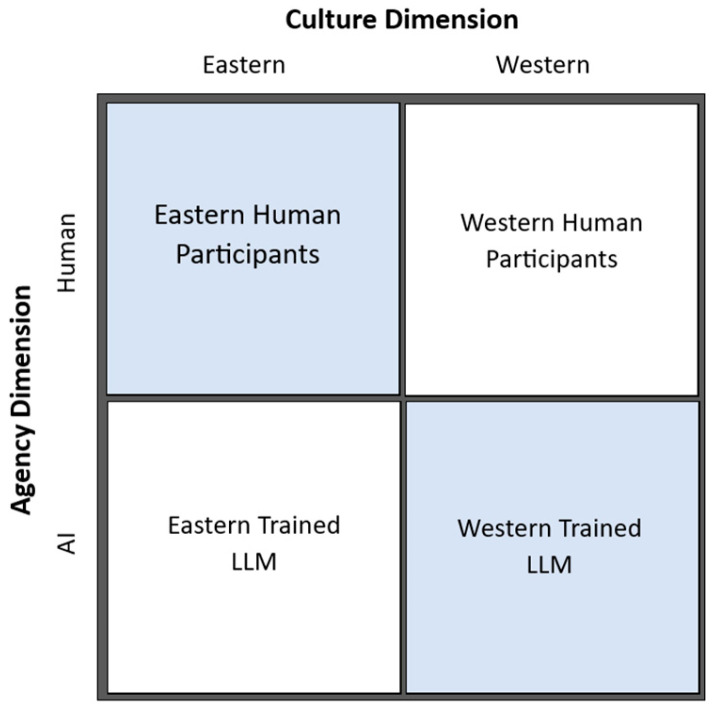
Subject types match each other by the agentic and cultural dimensions.

The overarching goal was to examine whether the creator–assessor (dis)similarity affects the assessment of creativity, alongside two dimensions: agentic similarity and cultural similarity. Four subject types were included in this study, namely LLMs trained in the West (ChatGPT 3.5 from the US), LLMs trained in the East (Kimi from China), participants from the West (university students from The Netherlands), and participants from the East (university students from China), which match each other by the dimensions of agency and culture (see Figure 1). To set up the study, first, stories were created by subjects from these groups (the creators). Then, these stories were assessed on their creativity by new subjects from these groups (the assessors), following a two (culture-match vs. culture-unmatch) by two (agency-match vs. agency-unmatch) within-subject experimental design. Three criteria were used for the assessment: overall creativity, and originality and appropriateness as two distinguishable measures that jointly defined creativity.

The overarching hypotheses were that both agentic similarity and cultural similarity have a main effect on the assessment of creativity. For the agency dimension, we did not hold specific hypotheses on the direction of the main effect. Instead, we hypothesized that there would be an overall preference towards human-created stories on the overall creativity and originality, but not on appropriateness. This was based on the fact that it is more likely that only humans would come up with something genuinely new, though likely less well-thought-out, which is not included in LLMs’ training data. Thus, our specific hypotheses were as follows: (a) when LLMs are the assessors, they would on average rate the LLM-created stories (agency-match) less original but more appropriate than human-created stories (agency-unmatch); and (b) when humans are the assessors, they would on average rate the human-created stories (agency-match) more original but less appropriate than LLM-created stories (agency-unmatch).

For the culture dimension, we further hypothesized that the assessors would rate the stories more original but less appropriate when the assessors and the creators do not match than when they do match, as a different culture might deliver something new but seemingly less appropriate. Nonetheless, the effect might also depend on the cultural background of the assessors, which according to the literature, shapes their conceptions of creativity–Westerners focus more on originality while Easterners focus more on appropriateness. Specifically, we hypothesized that Eastern assessors would rate Western creators’ stories (culture-unmatch) more original but less appropriate than Eastern creators’ stories (culture-match). This is because Western creators may emphasize more on originality but less on appropriateness in their creation, and Eastern assessors may hold a higher standard on appropriateness. In contrast, we hypothesized that Western assessors would rate stories from Eastern creators (culture-unmatch) less original but more appropriate than stories from Western creators (culture-match).

## 2. Method

### 2.1. Research Design, Subjects, and Overall Procedures

This study adopted an experimental design where assessors and creators were matched along two dimensions of culture and agency. Four types of subjects were involved in this study: (1) LLMs trained in the East, namely Kimi, an application developed by a company called ‘Moonshot’ from China, powered by its proprietary foundational model Moonshot_V1; (2) Human participants from the East, consisting of university students from mainland China, all of whom were native Chinese speakers; (3) LLMs trained in the West, namely ChatGPT (version 3.5), an application developed by OpenAI from the US; and (4) Human participants from the West, consisting of both Dutch or international university students recruited in The Netherlands, all of whom were proficient English speakers as self-reported with a Western background. This group is less homogeneous than the group of Chinese participants.

Because Chinese participants were all native Chinese speakers, and the Chinese AI were trained mainly on Chinese text, the gap between these two subject types was smaller than it was between the Western subject types. ChatGPT was predominantly trained on English text, while most Dutch participants were not native English speakers (except for one). However, despite the difference in their native languages, the Western human participants shared a Western cultural background.

The study was carried out in two stages. At the first stage, subjects were asked to write stories, and at the second stage, new subjects were recruited and asked to assess a subset of the stories randomly chosen from stories created at the first stage.

### 2.2. Stage 1: Story Creation—Subjects as Creators

#### 2.2.1. Testing Paradigm

Rather than giving a general prompt (e.g., ‘Please write a creative story’), the current study adopted a structured testing paradigm inspired by the Storyboard Task (Taylor et al. 2021) for guiding subjects to write stories. In the original Storyboard Task, subjects were presented with three images to incorporate into the beginning, middle, and end of their story. We modified this design by using three sentences of text to replace the images, one sentence corresponding to one image, in order to accommodate the fact that LLMs are not designed to directly interpret images. In addition, we introduced a control variable by locating the subjects in two different conditions: subjects were either instructed to write ‘a common, not very creative story’ (*be-uncreative*) or ‘an uncommon, very creative story’ (*be-creative*). The purpose of this design was to enable us to check whether the test paradigm works. As past research has revealed the profound influence of instructions on one’s performance on creative tasks (e.g., Forthmann et al. 2016), the expectation here was that, if the paradigm indeed works, then a random selection of stories generated under two conditions should significantly differ from each other respecting how creative they were. Having stories at both ends of the creative continuum would also introduce a certain level of diversity and help the assessors establish a more balanced reference for assessing all stories at the second stage. In addition, we instructed the participants to write the stories of 500–600 characters (for stories to be written in Chinese) or words (for stories to be written in English)^1^ out of two considerations. First, such an anchoring reference would prevent a drastic difference in the length of the stories, thus ensuring the quality of the stories is more comparable. Second, this word count would allow the stories to be sufficiently developed within a moderate duration of time to finish (namely 30–60 min), fitting the experimental set-up of this study. The exact instructions (both English and Chinese versions) for both the creative and non-creative conditions and the stories can be found in the Supplementary Materials.

#### 2.2.2. Specific Procedures per Subject Group

**Chinese Participants.** In total, 10 Chinese university students (ages ranged from 18 to 35 years, who were university students majoring in psychology; no personal information was collected due to privacy and ethical concerns) were recruited through a campus subject pool or via one of the authors’ personal connections. All participants volunteered to take part in this study without receiving financial rewards or university credits. They completed the task through the Tencent Survey platform, and each of them randomly received a survey link that directed them either to create ‘non-creative’ or ‘creative’ stories. All instructions were given in Chinese (simplified), and participants were asked to write their stories in any software they preferred and to submit the completed stories via a new survey link. Participants were advised to spend 30 to 60 min writing the story and to report the time taken (reported as ranging from 25 to 200 min). At the end of the survey, participants were asked whether they had used generative artificial intelligence during the task (none as reported). In total, 10 stories (5 creative and 5 uncreative) were collected, of which the character count ranges from 500 to 744.

**Dutch Participants.** In total, 13 Dutch university students (age above 16 years who were university students; most of them majoring in psychology; no personal information was collected due to privacy and ethical concerns) were recruited to create stories. Although the initial plan was to recruit 10 participants, 13 had signed up to take part in the study. All participants were recruited from the campus subject pool at ** (masked for review) university in The Netherlands. They completed the task via a Qualtrics survey and were rewarded with 1.0 participation point upon completing the task. All instructions were given in English, and the participants also wrote stories in English. As the same to the Chinese participants, Dutch participants were also advised to finish their story in 30–60 min and write the story using an offline writing software to prevent unexpected loss. Additional information was collected on the participants’ cultural background and their English proficiency. As shown by these data, all participants were European citizens with a Western cultural background, and while different types of English proficiency were present, most participants stated to be advanced English users. And none of the participants reported to have used generative artificial intelligence during the task. In total, 13 stories (6 creative and 7 uncreative) were collected, of which the word count ranges from 312 to 609.

**Kimi.** The moonshot-v1-8k version was used, and each conversation session was regarded as equivalent to a subject. The instructions used for prompting Kimi to generate stories were identical to those given to human participants, with one additional sentence at the beginning, ‘Pretend that you are a university student’, to match the human samples. All instructions were given in Chinese (simplified), and the stories were also generated in Chinese. Through 10 conversation sessions, 10 stories (5 creative and 5 uncreative) were collected (from 13–21 May 2024), of which the character count ranges from 526 to 602.

**ChatGPT.** The free version of ChatGPT-3.5 was used, and each conversation session was regarded as equivalent to a subject. The procedures were the same as those of obtaining stories in Kimi, except that all instructions were given in English. Through 10 conversation sessions, 10 stories (5 creative and 5 uncreative) were collected (from 13–23 May 2024), of which the word count ranges from 384 to 611.

#### 2.2.3. Story Selection & Modification

To restrict the time burden on human assessors at the second stage of the experiment, 16 stories in total were selected for evaluation. This was conducted by randomly selecting four stories (i.e., blindly drawing a piece of paper on which a number was written) from each subject group: 2 creative and 2 uncreative stories. After this, spelling errors were corrected to eliminate any potential effect of them on the assessment of creativity. Additionally, stories originally written in English were translated into Chinese and vice versa, first by ChatGPT and then scrutinized by two of the authors who are native Chinese speakers and also proficient English users (both have lived and/or worked in an English environment). To minimize potential cultural biases, culture-specific city names or character names were modified to more culturally neutral names. At the end, this gave a total of 16 stories (4 created by participants from China, 4 by Kimi, 4 by participants from the Netherlands, and 4 by ChatGPT), each having both a Chinese and an English version.

### 2.3. Stage 2: Story Assessment–Subjects as Assessors

At this stage, new subjects were recruited to assess the creativity of the selected stories collected at the first stage.

#### 2.3.1. Dimensions of Story Assessment

The stories were evaluated on the two core aspects of creativity: *originality* (=*novelty, uniqueness*, etc.) and *appropriateness* (=*effectiveness, usefulness*, etc.). As mentioned in Runco and Jaeger (2012), multiple terms have been used in the literature to refer to each of these aspects. In this research, we chose the specific terms as they fit better with the purpose of assessing the creativity of stories (e.g., rating the ‘usefulness’ of a story would sound odd). Moreover, we also included an overarching criterion of *creativity*, as a hallmark to check whether the separated rating of originality and appropriateness delivered more nuanced insights. In accordance with Bai et al. (2024), the rating was conducted on a 6-point Likert scale (1/2/3 = very/rather/a bit low and 4/5/6 = bit/rather/very high). We did not give a specific definition of the criterion of creativity, but instructed the participants to assess it based on their intuitive judgement of the stories. As for originality and appropriateness, specific definitions were given as follows (for detailed instructions, see the Supplementary Materials).

- **Originality** refers to an idea or product being unique from other ideas or products, typically with a lower probability of occurring among the general population, and most people would not think of it.
- **Appropriateness** refers to an idea or product being appropriate and valuable in the specific context wherein it is generated, typically (with some imagination) reasonable or realizable.

#### 2.3.2. Specific Procedures per Subject Group

**Chinese Participants.** In total, 30 Chinese university students (ages ranged from 18 to 35 years, who were majoring in psychology; also, no personal information collected here) were recruited through the same ways as mentioned before. All participants volunteered to take part in this study without receiving financial rewards or university credits. They completed the assessment of stories through the Tencent Survey platform, wherein the 16 stories were presented in a random order that was different for each participant. For each story, participants were first asked to rate their instinctive perception of the story’s creativity. They were then provided with definitions of ‘originality’ and ‘effectiveness’ and asked to evaluate the story on each of these criteria. Throughout the assessment, all instructions were given in Chinese (simplified).

**Dutch Participants.** In total, 41 Dutch university students (age above 16 years who were university students; most of them majoring in psychology; no personal information was collected due to privacy and ethical concerns) took part in this stage, who were recruited from the same subject pool as mentioned before. They completed the assessment of stories via a Qualtrics survey and were rewarded with 1.0 participation credit upon completing the assessment. The assessment procedures were the same as for Chinese participants, while all instructions were given in English. Data of 11 students were excluded as they did not seem to have completed the assessment seriously (e.g., finished unexpectedly faster than other participants). This led to a final sample of 30 participants whose data were included in further analysis. All participants had a European background, except for one who was Eurasian and two others who had both a European and an Asian background. Different types of English proficiency were present, but most participants stated to be advanced English learners.

**Kimi.** Again, the moonshot-v1-8k version was used, and each conversation session was regarded as equivalent to a subject. In total, 30 conversations were run, and within each session, Kimi was prompted to assess the 16 stories. The same instructions as presented to the Chinese participants were used, with an additional sentence at the beginning stating: ‘Pretend you are a university student’ to match the major characteristics of human participants. Note that here the 16 stories were given all together at once to Kimi right after the instructions. Data from 30 conversation sessions were collected (from 4–10 June 2024) for further analysis.

**ChatGPT.** Again, the free version ChatGPT-3.5 was used, and each conversation session was regarded as equivalent to a subject, and 30 conversations were run to collect data. The prompting process in ChatGPT was slightly adapted, and the instructions and the stories were presented stepwise in several messages, following the next sequence: (1) ChatGPT was prompted to act as a university student in the first message, then (2) an explanation of the assessment task was given in the second message, followed by (3) explanations of the assessing criteria in the third message, and (4) each story was presented to ChatGPT in a new message and ChatGPT was instructed to respond only with scores for the three assessing criteria (general creativity, originality, and appropriateness). Data from 30 conversation sessions were collected (from 4–6 June 2024) for further analysis.

### 2.4. Analysis Plan

Descriptive analyses were performed to obtain an overview of how different assessors differ in their ratings of stories created by different creators. Next, ICCs (two-way random) were calculated for each subject type to provide insight into how reliable the raters’ assessments were, including both the absolute and the consistency agreement. Finally, two sets of Pearson’s correlations were calculated: (a) between creativity, originality, and appropriateness, to check whether the separate rating of originality and appropriateness did bring more nuances, regarding both the raw scores (incl. every assessor’s scores of each story) and the aggregated scores per assessor type (mean of all assessors’ scores of each story per assessor type), and (b) between the assessor types on creativity, originality, and appropriateness, regarding only the aggregated scores per assessor type.

Multilevel linear regression was employed to test our hypotheses, given the repeated-measure design (i.e., measures [level 1] nested in individual participants or chat sessions [level 2]). Note that, for this part of the analysis, we only reported on the results of models including originality and appropriateness as the outcome variables for the sake of conciseness, as creativity and originality were found to be highly correlated, and relevant models yielded comparable results. In addition, the outcome variables were standardized to enable the comparison of the effects of the same predictors on both outcome variables. Prior to the formal analysis, we conducted a control analysis on whether the *story type* (creative = 1, uncreative = 0; level 1 predictor), namely whether a story was created under the be-creative or be-uncreative conditions at Stage 1, to check whether the test paradigm of story creation worked (M_control_).

Next, in the formal analysis, we first examined the main effects of *agentic and cultural similarity*, coded at the measurement level based on whether the assessor and the creator of a story match on each of the similarity dimensions (match = 0.5, unmatch = −0.5; level-1 predictor), on each of the outcome variables (M1). This helped us to understand whether the similarity between the creator and the assessor, respectively, in agency or in cultural background, did make an impact on the creator’s creativity assessment. Second, we examined the interaction effects of assessor type by the creator–assessor similarity on the outcome variables (M2), coded at the individual level (level-2 predictor). Specifically, assessors were coded on the agentic dimension (*assessor agency)* either as human (=0.5) or AI (=−0.5) subjects, and the interaction item was constructed through multiplying assessors’ agency type by creator–assessor agentic similarity (*assessor agency × ageny match*). For the cultural dimension, assessors were coded either as Chinese (=−0.5) or Western (=0.5) subjects, and the interaction item was constructed through multiplying assessors’ cultural type by creator–assessor culture similarity (*assessor culture × culture match*). Note that, in all M3 models, we did not set the slopes of level-1 predictors (i.e., *agency match* and *culture match*) random to keep the models simple. According to Snijders and Bosker (2011, p. 82), when an interaction effect is established based on substantive arguments as in the present study, it is plausible to test the cross-level interaction effects without setting the slope random, given the test power of a fixed interaction effect is considerably higher than the test power of the corresponding random slope.

We employed the R (version 4.3.3) package nlme for running the multilevel linear regression models. Compared to other software (e.g., SuperMix version 2.1) or the R package lmer4, nlme is advantageous in that it supports explicit modeling of the residual variance across groups. This is especially useful when the homoscedasticity assumption is violated, which was the case with our data (the residual decreased when the fitted value increased). This was not unexpected, as four types of subjects were included in our study, and indeed, heteroscedastic models allowing different variances across subject types showed much better model fits than homoscedastic models. No other assumptions were violated. We adopted *p* < 0.05 to determine the significance of effects.

## 3. Results

### 3.1. Descriptive Statistics

**Figure 2:**
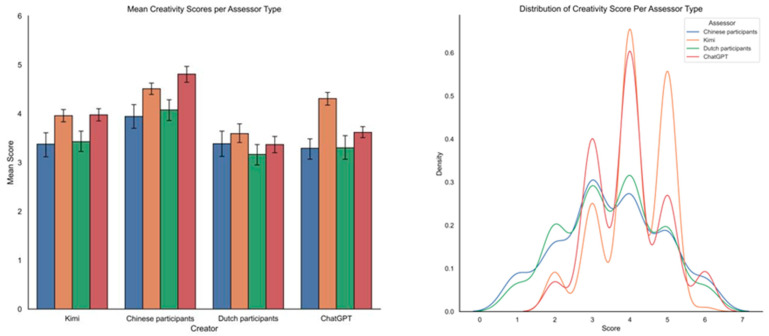

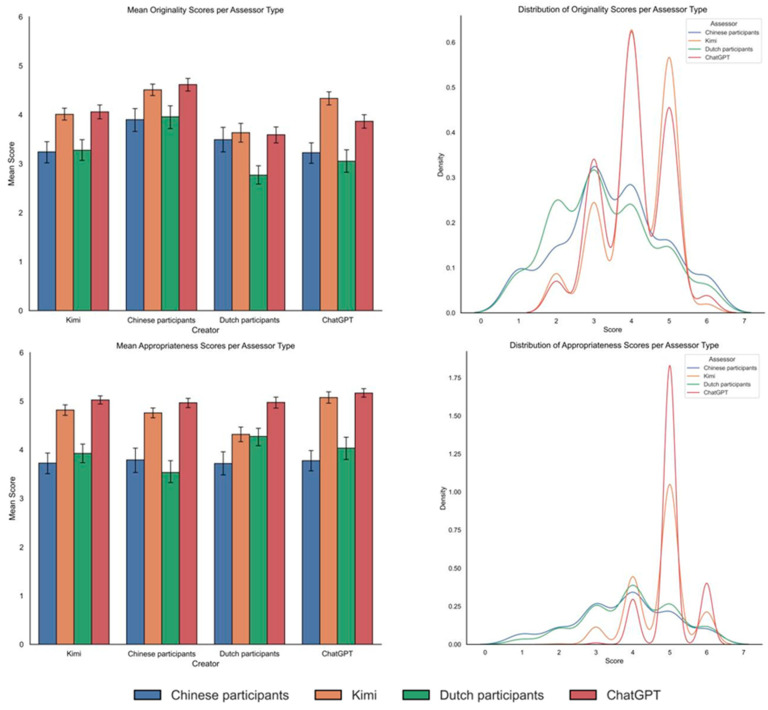
Mean with 95% confidence interval (**left panel**) and distribution (**right panel**) of creativity, originality, and appropriateness scores.

Table 1 presents the mean (*M*) and standard deviation (*SD*) of the scores given on the three assessed dimensions by different types of assessors, and Figure 2 provides a visualization of the mean scores and the distribution of scores across assessor types. There were some observable tendencies. First, (a) stories written by Chinese participants seemed to be scored the highest on the overall creativity and originality from all different assessor types, though this was not the case for scores on appropriateness, and (b) stories written by Dutch participants seemed to be scored, on average, the lowest on the overall creativity, except in assessments from Chinese participants.

Second, as shown in the right panel of Figure 2, the scores given by Kimi and ChatGPT were less spread out, i.e., less varied than those from human assessors, particularly regarding appropriateness. Indeed, also in Table 1, the standard deviations of Kimi and ChatGPT (bold and underlined) were noticeably smaller than that of either Chinese or Dutch participants (hence, heteroscedastic models were needed later on). Notably, both Kimi and ChatGPT never assigned a score of 1 in their assessment, whereas human assessors did in some cases. Collectively, these results suggest that scores by AI assessors had lower discriminant validity than scores by human assessors.

### 3.2. ICCs and Correlations

Table 2 presents the ICCs for each subject type. An exceptional pattern was that ICCs for Kimi were very high, even for single measures, which were observed to be much lower for other assessor types. ChatGPT, however, showed a more similar pattern in ICCs as human assessors, all indicating that single measures are much less reliable than average measures. Another noticeable difference was that human assessors (especially Chinese participants) showed higher reliability in assessing creativity and originality, whereas Kimi and ChatGPT showed comparable reliability in all criteria.

The correlation results showed that, based on the raw scores, creativity is highly correlated with originality (*r* = 0.835, *p* < 0.0001), while its correlation with appropriateness is significantly lower (*r* = 0.392, *p* < 0.0001). Additionally, the correlation between originality and appropriateness is 0.393 (p < 0.0001). Table 3 presents the correlations calculated based on aggregated scores per assessor type. The correlations between different scores (creativity, originality, and appropriateness) revealed a contrast between human and AI assessors: for both Kimi and ChatGPT, creativity and originality were highly positively correlated with appropriateness, while for human assessors, these correlations were very low (for Chinese participants) or even negative (for Dutch participants). This indicates that the distinction between originality and appropriateness as two different aspects of creativity was not presented in AI assessors, whereas it was presented in human assessors.

The correlations between different assessor types (Chinese participants, Kimi, Dutch participants, and ChatGPT) revealed that, on creativity and originality, ChatGPT’s scores were not correlated to any of the other assessors’ scores, whereas the other assessors’ scores were all moderately to highly correlated. On appropriateness, however, none of the assessors’ scores were shown to be correlated with any of the others.

### 3.3. Multilevel Linear Regression

The results of the multilevel regression analyses are presented in Table 4. The results of the control analysis showed that compared to the intercept-only model (M0), adding the main effect of story type improved model fit for both originality and appropriateness (significant changes in model deviance). The effects of story type were always positive and significant, though larger for originality than for appropriateness. This indicates that the stories created under creative conditions were much higher in originality and moderately higher in appropriateness than stories created under uncreative conditions.

In the formal analysis, first, compared to the intercept-only model (M0), adding the main effects of agentic match and cultural match (M1) improved model fits for both originality and appropriateness (significantly reduced model deviance compared to M0). Second, adding the main effects of assessor types and the interactions between assessor–creator similarities and assessor types (M2) further improved the model fits (significantly reduced model deviance compared to M1).

#### 3.3.1. Main and Interaction Effects of Agentic Similarity

The main effect of agentic similarity (match = 0.5 and unmatch = −0.5) was positive for both originality and appropriateness. This indicates that assessors, if not considering who the assessors were, gave higher originality and appropriateness scores to stories from creators who matched with them than to stories from creators who did not match with them in agency. Moreover, this influence is stronger for appropriateness than for originality when only considering the main effects of similarities (M1), but this trend flipped when also the main effects of assessor types and their interactions with similarities were considered.

**Figure 3:**
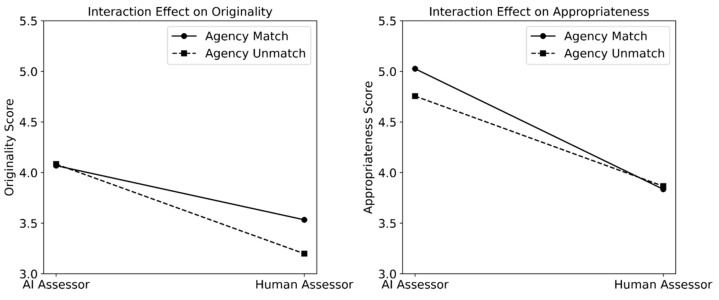
Visualization of the interaction effects between creator–assessor agentic similarity and assessor agency type, separately for originality (**left**) and appropriateness (**right**).

The interaction effect between agentic similarity and assessors’ agency type (AI vs. human) was positive for originality but negative for appropriateness, with comparable effect size. We visualized the interaction effect for each of the measures in Figure 3, setting all predictors related to the cultural dimension to zero. As shown in the figures, AI assessors gave comparable originality scores to both AI-created stories (match) and human-created stories (unmatch), whereas human assessors gave higher originality scores to human-created stories (match) than AI-created stories (unmatch). As for appropriateness, AI assessors gave higher scores to AI-created stories (match) than to human-created stories (unmatch), while human assessors gave comparable scores to both human-created stories (match) and AI-created stories (unmatch).

#### 3.3.2. Main and Interaction Effects of Cultural Similarity

The main effect of cultural similarity (match = 0.5 and unmatch = −0.5) was negative for originality but positive for appropriateness. This indicates that assessors, if not considering who they were, gave lower originality but higher appropriateness scores to stories from creators who matched with them than to stories from creators who did not match with them in culture. Moreover, this influence is stronger for originality than for appropriateness, consistent across models.

**Figure 4:**
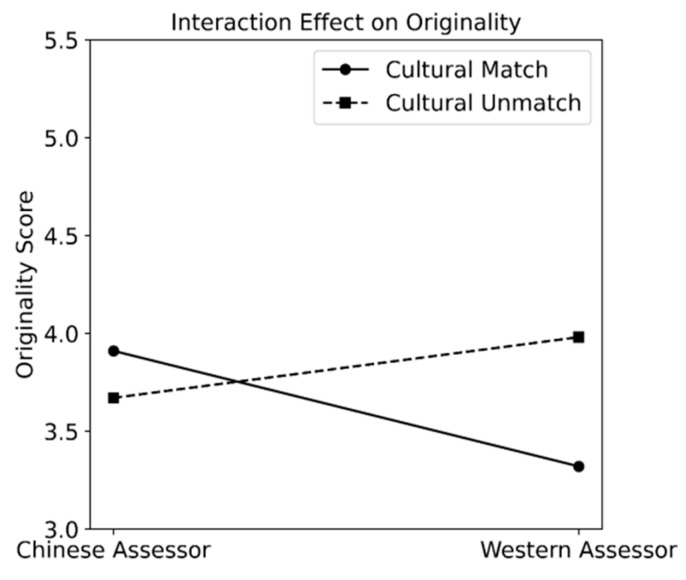
Visualization of the interaction effects between creator–assessor cultural similarity and assessor culture type for originality.

The interaction effect between cultural similarity and the assessors’ culture type (Chinese or Western) was negative for originality. For appropriateness, the interaction effect was not significant. We visualized the interaction effect for originality in Figure 4, setting all predictors related to the agency dimension to zero. As shown, Chinese assessors gave higher originality scores to Chinese creators’ stories (match) than to Western creators’ stories (unmatch). In contrast, Western assessors gave lower originality scores to Western creators’ stories (match) than to Chinese creators’ stories (unmatch). For both cases, the differences were rather observable.

## 4. Discussion

Inspired by Arendt’s (2013) notion of plurality on human beings—while no two people are interchangeable, all human beings belong to the same species and are sufficiently alike to understand and to assess another’s actions (Tömmel and d’Entreves 2024)—we set out in the present study to question the robustness of employing humans in evaluating AI models’ creativity. Through an experiment using a within-subject design, the specific goal of this study was to examine the effects of creator–assessor (dis)similarities on the assessment of creativity. We involved four types of subjects as both the creators and the assessors, including LLMs trained in the West (ChatGPT 3.5 from the US), LLMs trained in the East (Kimi from China), participants from the West (university students from The Netherlands), and participants from the East (university students from China), which can be considered to match each other by the agentic dimension (AI vs. humans) and by the cultural dimension (Chinese vs. Western backgrounds). We first collected and selected 16 stories created by these subjects (per subject type 4 stories: 2 uncreative and 2 creative). Then, these stories were assessed on their overall creativity, originality, and appropriateness by new subjects from these groups. Thus, for each of the measures, the assessors might match the creators on either the agentic or the cultural dimension, or both, or none. In a nutshell, our main findings are that both agentic and cultural similarities between the creators and the assessors affected the assessment of creativity, and these effects differed based on who the assessors were. A general implication of these findings points to that creativity judgments are not made in isolation with the assessors themselves; instead, they are shaped by the relational and contextual ground shared between the creator and the assessor.

### 4.1. Effects of Agentic Similarity on Assessing Creativity

For the agentic dimension, the assessors on average gave higher ratings on both originality and appropriateness when the assessors and the creators matched than when they did not match. However, when taking into account who the assessors were, more nuances emerged. For originality, the effect of similarity mainly came from human assessors. Human assessors gave observably higher originality scores to human- than AI-created stories (match > unmatch), while AI assessors gave comparable originality scores to both human- and AI-created stories. For appropriateness, the effect of similarity mainly came from AI assessors. AI assessors generally rated AI-created stories as more appropriate than human-created stories (match > unmatch), while human assessors gave comparable appropriateness scores to both AI- or human-created stories.

These findings confirmed our hypotheses that the similarity between the assessor and the creator matters for the assessment of creativity. In addition, the finding that human assessors exhibited a stronger preference for originality in human- over AI-generated ones aligns with our hypothesis, in that only humans would come up with something genuinely new, which is not included in LLMs’ training data. This might be especially true given that we have not used the most advanced LLM models (e.g., ChatGPT Plus version). Moreover, there might also be another reason. While we have masked the creators of the stories during the assessment, human assessors might have (un)consciously recognized AI-generated stories and thus, become biased while rating these stories as past research has suggested (Hattori et al. 2024; Magni et al. 2024). Though no data were systematically collected to address this, several Chinese assessors have approached and asked the experimenter whether the stories were generated by AI tools after their participation.

Yet, an interesting contrast is that AI assessors showed no preference for originality towards human- or AI-created stories, as against our hypothesis. This might point to either that human-created stories do not genuinely differ from AI-generated stories on originality or that AI models do not perform well in assessing and distinguishing the originality of stories. In fact, the latter explanation might be more likely. As shown in the descriptive statistics, compared to human assessors’ ratings, AI assessors’ scores had less variety in scores, and they seldom gave scores at the lower bound of the scales (especially regarding appropriateness). These findings prompted a deeper reflection on the probabilistic and pattern-based nature of LLMs, which optimize for coherence rather than subjective salience. Unlike human raters, AI models lack embodied experience, contextual flexibility, and intuitive surprise—elements that might be vital for recognizing originality.

Finally, the finding that AI assessors showed a clear preference for appropriateness towards AI- than human-created stories was also consistent with our hypothesis. Nevertheless, human assessors did not show a preference for appropriateness towards AI- than human-created stories. This might again be explained by the probabilistic and pattern-based nature of LLMs, which primarily apply systematic criteria such as writing clarity and/or the syntactic or linguistic features to judge the appropriateness of stories. In contrast, human evaluators—drawing on multifaceted cognitive processes and personal context—may not adhere as rigidly to predefined norms when judging appropriateness. In fact, in accordance with the newest theories of creativity (Corazza 2021; Glăveanu 2013; Sawyer 2021), assessors (or audience as named in many creative domains such as performance art) also form an important part of the creation. That said, while judging a creation, the assessors are not passive observers; instead, they also use their own creativity to make sense of the creation. Following this account, the finding that AI assessors clearly rated AI-created stories higher on appropriateness than human-created stories might rightly point to their lack of creativity. Future research is recommended to also investigate the creativity of AI models through specifically asking them to rate the creative outcomes’ appropriateness and compare it to humans’ ratings.

### 4.2. Effects of Cultural Similarity on Assessing Creativity

For the cultural dimension, the assessors gave lower scores on originality when the assessors and the creators matched than when they did not match. However, when taking into account who the assessors were, it was shown that the effect of creator–assessor cultural similarity on originality was quite oppositional for Chinese and for Western assessors. Specifically, Chinese assessors gave higher originality scores to Chinese creators’ stories than to Western creators’ stories (match > unmatch). In contrast, Western assessors gave lower originality scores to Western creators’ stories than Chinese creators’ stories (match < unmatch). For appropriateness, the assessors on average gave higher appropriateness scores to stories when they matched with the creators than when not matched, even when taking into account who the assessors were.

While confirming our general hypothesis that cultural similarity matters for the assessment of creativity, other specific findings were not all as hypothesized. First, there was an overall preference for originality towards Chinese creators’ stories than Western creators’ stories, while we have hypothesized the opposite, given that Western cultures often place more emphasis on the originality aspect of creativity and offer more space for original thoughts (Niu and Kaufman 2013). One potential explanation for this contradictory finding is related to a limitation of the current research, linked to the linguistic features of Chinese and English. When we asked the subjects to write the stories, an instruction to limit the stories to 500–600 words (in English) or characters (in English) was given for all subject types, to keep the testing time to a reasonable amount yet sufficient for writing a story. However, after having translated the stories to make bilingual versions, we found that the Chinese version of the stories was rather consistently shorter than the English version of the stories. This showed that, with the same word limit, subjects could have expressed more in Chinese than in English, which might have left more space for developing more creative, original stories.

Second, the finding that the assessors overall gave higher appropriateness scores to stories from creators with the same cultural background than to stories from creators with another cultural background was as hypothesized. Nonetheless, the absence of the interplay between assessor–creator cultural similarity and assessors’ culture type was not as expected. Given that Chinese culture emphasizes the appropriateness aspect of creativity more than Western cultures (Niu and Kaufman 2013), we have expected stories from Chinese creators to show more appropriateness than stories from Western creators. This should have been especially given the aforementioned limitation on the word/character limit as set, which could have offered more space for Chinese creators to elaborate on their stories than it was for Western creators.

Yet, it is worth noting that our hypotheses on the cultural dimension were mainly built based on past research that used structured creative thinking tasks (Ivancovsky et al. 2018; Liou and Lan 2018; Niu et al. 2007; Niu and Sternberg 2001; Wong and Niu 2013; c.f., Huntsinger et al. 2011; Storme et al. 2017; Niu and Sternberg 2002), wherein creativity is embedded in short responses and not much context dependent. Creativity as embedded in stories, however, is likely situated in culturally sensitive contexts, which were overlooked in formulating our hypotheses. In this account, the assessors might have had trouble following the contexts as described in the stories from creators who do not share the same cultural background, and thus, have difficulties in perceiving the stories as fully appropriate. This again points to the importance of the relational and contextual ground shared between the creator and the assessor, which goes beyond whether a story is fully developed within the assessor’s own, isolated context.

### 4.3. Limitations, Implications, and Future Research

The current study has several major limitations. First, we have not used the most advanced LLM models in the current study due to a lack of financial aid and the explorative nature. The newest LLM released by OpenAI, ChatGPT-4, is trained on significantly more parameters and has shown much better performance on different cognitive tasks than ChatGPT-3.5. Moreover, the recently released DeepSeek-V3, developed by the Chinese company DeepSeek-AI, has demonstrated remarkable performance improvements despite being trained with fewer parameters and less data. Specifically, DeepSeek-V3 is an open-source model that optimizes inference efficiency, achieving performance comparable to leading closed-source models like GPT-4o (DeepSeek-AI et al. 2024). Likewise, DeepSeek-R1, designed for reasoning tasks using reinforcement learning, has outperformed several existing models in mathematics, programming, and complex reasoning benchmarks (DeepSeek-AI et al. 2024). Future research should explore larger-scale experiments on these advanced models, not only to validate our findings but also to leverage the greater transparency of open-source models for deeper investigation.

Second, as aforementioned, with the same word/character limit, subjects seemingly have expressed more in Chinese than in English, and this might have biased our findings on the cultural dimension. Future research should take this into account when adopting the same testing paradigm by, for instance, giving different word limits in Chinese and in English, which may require inputs from linguistic experts or more rigorous pilot research. Alternatively, this issue can also be indirectly addressed by adopting some measures to account for the linguistic features of created stories, such as the perceived richness of stories as control variables. This might also help reveal the potential differences in writing clarity between AI- and human-created stories and enable examining the bounded impact thereof on the assessment of creativity. Next, on the cultural dimension, another limitation was that we assumed Chinese and Western LLMs only differ in their cultural backgrounds, not taking into account that the models’ architecture might be significantly different. Yet, given that little is known about how LLM architecture affects its performance, it is also hard to gauge the impact of this limitation on our findings.

Third, a structural limitation of using LLMs as creativity assessors lies in their tendency toward homogenization. Although LLMs can generate outputs that appear original, recent studies have shown that similar prompts often lead to overlapping ideas (Agarwal et al. 2025; Bommasani et al. 2022). This likely results from optimization algorithms favoring safe and generalizable responses over highly divergent or unconventional ones. Consequently, the overall creative space may lack diversity and depth, limiting the model’s ability to detect rare or boundary-pushing ideas. Future research should critically examine this bias to avoid reinforcing normative thinking when integrating LLMs into evaluative frameworks. Meanwhile, refining model selection and cross-linguistic design remains essential for more inclusive and interpretable assessments.

Finally, there are other limitations that are also worth mentioning. For instance, different subject types seem to differ from each other in anchoring their scores. In particular, our results showed that AI assessors’ scores had smaller ranges and SDs (i.e., focused on the few scoring points in the middle) compared to human assessors’ scores. Moreover, distinguishing originality and appropriateness as two aspects of creativity were seemingly only present in human assessors (low or negative correlations) but not in AI assessors (high, positive correlations). Furthermore, potential biases might also arise from the specific human samples recruited. For instance, Dutch participants did not fully match ChatGPT as English is still their second or third language.

Collectively, the present study presents an explorative, first step in examining the validity of using humans to assess AI models’ creativity (or vice versa). While caution is warranted, our findings provided preliminary evidence to highlight the importance of the relational and contextual ground shared between the creator and the assessor for the assessment of creativity. On this ground, future research is advised to incorporate both AI models and humans in the process of assessing creativity, to make use of the complementary strengths: (a) humans excel in capturing contextual subtlety, cultural nuance, and subjective resonance; and (b) AI models offer speed, consistency, and scalability. For instance, especially in large-scale evaluation, AI models could serve as efficient co-raters or first-pass filters, while humans could serve as final judges. Importantly, this also urges future design of creativity assessment systems to integrate AI in ways that are transparent, culturally sensitive, and methodologically grounded, allowing humans to strategically leverage rather than passively rely on AI assessments.

## 5. Conclusions

The present study questioned the robustness of employing humans in evaluating AI models’ creativity and provided first empirical evidence to confirm that creator–assessor (dis)similarities along the agentic (human vs. AI) and the cultural (English- vs. Chinese-speaking) dimensions matter for the assessment of creativity. For the agentic dimension, human assessors showed a pronounced preference for originality towards human-created stories than AI-generated stories, whereas AI assessors showed a similar but neglectable preference; in contrast, AI assessors showed a pronounced preference for appropriateness towards AI-generated stories than human-created stories, whereas human assessors showed a similar but neglectable preference. For the cultural dimension, both Chinese and Western assessors showed a pronounced preference for originality towards Chinese creators’ stories than Western creators’ stories; as for appropriateness, the assessors, regardless of their cultural backgrounds, always preferred stories from creators from the same cultural backgrounds. These findings suggest that we should be cautious in claiming whether AI models are creative or not based solely on humans’ assessment.

## Figures and Tables

**Table 1:** Descriptive statistics of creativity rating scores (*n* = 120, 30 per subject type).

		Creativity		Originality		Appropriateness	
Assessor	Creator	*M*	*SD*	*M*	*SD*	*M*	*SD*
Chinese participants	**All creator types**	3.50	**1.34**	3.46	**1.34**	3.75	**1.30**
	Chinese participants	3.94	1.34	3.90	1.39	3.79	1.38
	Kimi	3.38	1.32	3.24	1.24	3.73	1.24
	Dutch participants	3.38	1.43	3.49	1.44	3.72	1.40
	ChatGPT	3.29	1.18	3.23	1.16	3.78	1.18
Kimi	**All creator types**	4.09	**0.87**	4.12	**0.89**	4.74	**0.75**
	Chinese participants	4.51	0.65	4.51	0.68	4.76	0.59
	Kimi	3.96	0.69	4.01	0.72	4.82	0.59
	Dutch participants	3.59	1.07	3.63	1.09	4.32	0.90
	ChatGPT	4.31	0.74	4.33	0.75	5.08	0.68
Dutch participants	**All creator types**	3.49	**1.27**	3.26	**1.31**	3.94	**1.21**
	Chinese participants	4.08	1.18	3.96	1.28	3.53	1.31
	Kimi	3.43	1.14	3.28	1.25	3.93	1.07
	Dutch participants	3.17	1.13	2.77	1.07	4.28	1.00
	ChatGPT	3.30	1.42	3.05	1.35	4.03	1.32
ChatGPT	**All creator types**	3.94	**0.96**	4.03	**0.90**	5.03	**0.54**
	Chinese participants	4.81	0.84	4.62	0.72	4.97	0.56
	Kimi	3.98	0.73	4.06	0.81	5.03	0.46
	Dutch participants	3.37	0.89	3.59	0.94	4.98	0.61
	ChatGPT	3.62	0.68	3.87	0.78	5.17	0.49

**Table 2:** ICCs (two-way random) between assessors for each subject type.

Assessor	ICC Type		ICC [95% CI]		
			Creativity	Originality	Appropriateness
Chinese participants	Absolute agreement	Single measures	0.14 [0.07, 0.30]	0.15 [0.08, 0.31]	0.02 [0.00, 0.06]
		Average measures	0.83 [0.69, 0.93]	0.84 [0.71, 0.93]	0.33 [0.01, 0.66]
	Consistency	Single measures	0.24 [0.13, 0.45]	0.23 [0.12, 0.43]	0.03 [0.00, 0.11]
		Average measures	0.91 [0.82, 0.96]	0.90 [0.81, 0.96]	0.48 [0.03, 0.79]
Kimi	Absolute agreement	Single measures	**0.82 [0.71, 0.92]**	**0.78 [0.65, 0.90]**	**0.58 [0.42, 0.77]**
		Average measures	0.99 [0.99, 1.0]	0.99 [0.98, 1.0]	0.98 [0.96, 0.99]
	Consistency	Single measures	**0.82 [0.71, 0.92]**	**0.81 [0.70, 0.91]**	**0.65 [0.49, 0.82]**
		Average measures	0.99 [0.99, 1.0]	0.99 [0.99, 1.0]	0.98 [0.97, 0.99]
Dutch participants	Absolute agreement	Single measures	0.24 [0.13, 0.44]	0.31 [0.18, 0.52]	0.11 [0.05, 0.25]
		Average measures	0.90 [0.82, 0.96]	0.93 [0.87, 0.97]	0.78 [0.61, 0.91]
	Consistency	Single measures	0.29 [0.17, 0.51]	0.38 [0.24, 0.61]	0.14 [0.07, 0.31]
		Average measures	0.93 [0.87, 0.97]	0.95 [0.91, 0.98]	0.83 [0.68, 0.93]
ChatGPT	Absolute agreement	Single measures	0.13 [0.06, 0.28]	0.09 [0.04, 0.21]	0.09 [0.04, 0.22]
		Average measures	0.81 [0.65, 0.92]	0.74 [0.52, 0.89]	0.75 [0.54, 0.89]
	Consistency	Single measures	0.12 [0.06, 0.28]	0.09 [0.04, 0.22]	0.10 [0.04, 0.24].
		Average measures	0.81 [0.64, 0.92]	0.75 [0.53, 0.90]	0.77 [0.56, 0.90]

**Table 3:** Correlations (aggregated scores per assessor type): (a) between creativity, originality, and appropriateness; (b) between different assessor types.

(a)	Creativity	Originality	Appropriateness		Creativity	Originality	Appropriateness
	Chinese participants (above diagonal)/ Kimi (below diagonal)				Dutch participants (above diagonal)/ ChatGPT (below diagonal)		
**Creativity**		0.97 **	0.20			0.98 **	−0.86 **
**Originality**	1.00 **		0.18		0.85 **		−0.92 **
**Appropriateness**	0.87 ***	0.87 **			0.83 **	0.89 **	
**(b)**	**Chinese participants**	**Kimi**	**Dutch participants**	**ChatGPT**	**Kimi**	**Dutch participants**	**ChatGPT**
	Creativity (above diagonal)/Originality (below diagonal)				Appropriateness		
**Chinese participants**		0.81 **	0.74 **	−0.06	0.09	0.03	−0.15
**Kimi**	0.75 **		0.68 **	−0.02		−0.26	0.21
**Dutch participants**	0.65 **	0.66 **		−0.02			−0.08
**ChatGPT**	−0.07	0.25	0.19				

** *p <* 0.01. *** *p* < 0.001.

**Table 4:** Multilevel logistic regression analyses (n = 120, including 30 participants or sessions per subject type, and 1920 units at the measurement level).

	Originality				Appropriateness			
Models	M0	M_control_	M1	M2	M0	M_control_	M1	M2
Predictors	Coefficient (s.e.)							
Intercept	0.01 (0.05)	−0.37 (0.05) ***	0.01 (0.05)	1.08 (0.18) ***	0.02 (0.06)	−0.12 (0.06) ^+^	0.02 (0.06)	1.05 (0.19) ***
Story type		0.77 (0.03) ***				0.27 (0.03) ***		
Agentic match			0.09 (0.04) *	0.13 (0.04) ***			0.14 (0.03) ***	0.09 (0.03) *
Cultural match			−0.17 (0.04) ***	−0.17 (0.04) ***			0.10 (0.03) ***	0.09 (0.03) **
Assessor agency				−0.60 (0.08) ***				−0.92 (0.09) ***
Assessor culture				−0.12 (0.08)				0.22 (0.09) *
Agentic match *×* assessor agency				0.31 (0.08) ***				−0.23 (0.07) ***
Cultural match *×* assessor culture				−0.75 (0.07) ***				0.07 (0.06)
**Random part**	**Variance (s.e.)**							
σ^2^_subject_	0.75	0.57	0.77	0.74	0.64	0.58	0.62	0.61
σ^2^_error_	0.50	0.50	0.50	0.39	0.66	0.66	0.66	0.45
**Deviance** (*df*)	5037.02 (6)	4587.50 (7)	5011.86 (8)	4846.33 (12)	4371.72 (6)	4286.81 (7)	4337.26 (8)	4245.93 (12)
**AIC**	5049.02	4601.50	5027.86	4870.33	4383.72	4300.81	4353.26	4269.93
**BIC**	5082.38	4640.42	5072.34	4937.05	4417.08	4339.73	4397.74	4336.65
**Change in deviance** (Δ*df*)		449.52 (1) ***	9.16 (2) *	165.53 (4) ***		84.91 (1) ***	34.46 (2) ***	125.79 (4) ***

*Note.* M0 = intercept-only models. M_control_ = control models. M1 = main effects model. M2 = interaction effects model. Deviance = −2 * log likelihood. *^+^ p* < 0.10. * *p* < 0.05. ** *p <* 0.01. *** *p* < 0.001.

## Data Availability

The original contributions presented in this study are included in the article/Supplementary Materials. Further inquiries can be directed to the corresponding authors.

## Note

1 In Chinese, the basic meaningful linguistic unit is character, and a word can consist of a single character (e.g., the single character of “我” equals to “I” in English) or two or more characters (e.g., “人工智能” equals to “artificial intelligence” in English).

## Supplementary Materials

The following supporting information can be downloaded at: https://www.mdpi.com/article/10.3390/jintelligence13070080/s1. The exact instructions (both English and Chinese versions) for both the creative and non-creative conditions and the stories.
