# Supplementary material for: The Effects of (Dis)similarities Between the Creator and the Assessor on Assessing Creativity: A Comparison of Humans and LLMs
Source: J Intell. 2025 Jul 3;13(7):80. doi: 10.3390/jintelligence13070080 (PMC12295035; doi:10.3390/jintelligence13070080)
Supplement: Supplementary file 1 [file jintelligence-13-00080-s001.zip › Supplementary Folder/Additional information.pdf]

This supplementary folder includes all the anonymized data that was collected during the first and second stage of the experiment. In 'Stage 1 - Story Collection' one finds two subsequent folders where all the originally collected stories can be found, and the 16 randomly selected stories, as well as a document showing the instructions that were given to subjects. In 'Stage 2 - Story Assessment' an excel file with the ratings can be found, as well as a document showing the instructions. Beneath can be found some additional information regarding the timeline for data collection (helpful for knowing the versions of the AI's), the character/word counts of the stories, and information on how the cohorts in the assessment dataset are encoded.

Timeline Data Collection

Stage 1 - story collection:	
Eastern humans:	2024/05/13 - 2024/05/21
Eastern AI:	2024/05/13 - 2024/05/21
Western humans:	2024/05/13 - 2024/05/23
Western AI:	2024/05/13 - 2024/05/23
Stage 1 - story translation:	2024/05/26 - 2024/05/31
Stage 1 - picking the 16 stories:	2024/05/31
Stage 1 - modification by two bilingual authors:	2024/06/01 - 2024/06/04
Stage 2 - story assessment:	
Eastern humans*:	2024/06/04 - 2024/10/31
Eastern AI:	2024/06/04 - 2024/06/10
Western humans*:	2024/06/04 - 2024/10/20
Western AI:	2024/06/04 - 2024/06/06

*Because the original project had been subject to other deadlines in the summer of 2024, and because we could not reach the satisfied amount of 30 human participants back then, the time period for collecting human assessment data became longer. However, this will likely not have influenced the outcomes, because the stories used were still the same, and because data concerning AI assessments was all collected in the same period.

Character and Word Count of Originally Collected Stories

Original Eastern human stories character count:
1 = 569
2 = 518
3 = 500
4 = 565
5 = 512
6 = 541
7 = 594
8 = 541
9 = 744
10 = 504

Original Kimi stories character count:
1 = 602
2 = 597
3 = 535
4 = 594
5 = 577
6 = 533
7 = 544
8 = 526
9 = 599
10 = 562

Original Western human stories word count:
1 = 312
2 = 406
3 = 485
4 = 478
5 = 509
6 = 599
7 = 552
8 = 563
9 = 533
10 = 586
11 = 521
12 = 467
13 = 609

Original ChatGPT stories word count:
1 = 609
2 = 476
3 = 384
4 = 588
5 = 603
6 = 422
7 = 611
8 = 602
9 = 582
10 = 451

Cohort encoding
In the Assessment Dataset, cohorts are encoded as follows:
1 = Eastern human participant
2 = Eastern AI - Kimi
3 = Western human participant
4 = Western AI - ChatGPT
