# Supplementary material for: The Effects of (Dis)similarities Between the Creator and the Assessor on Assessing Creativity: A Comparison of Humans and LLMs
Source: J Intell. 2025 Jul 3;13(7):80. doi: 10.3390/jintelligence13070080 (PMC12295035; doi:10.3390/jintelligence13070080)
Supplement: Supplementary file 1 [file jintelligence-13-00080-s001.zip › Supplementary Folder/Stage 1 - Story Collection/Originally Collected Stories/Chinese AI - Kimi/Story 1 - Creative.pdf]

**Chinese original version**

在一座繁华的大城市中，一条熙熙攘攘的街道上，人群如潮水般涌动。街道两旁，霓虹灯闪烁，高楼大厦直插云霄。在这样一个充满活力的地方，有一个小巷，安静而神秘。

小巷的尽头，有一家不起眼的小店，名为“味觉之旅”。这家店的主人是一个年轻的女子，名叫莉莉。她拥有一种特殊的能力——能够创造出让人回味无穷的美食。每当夜幕降临，人们纷纷来到这里，寻找那一份独特的美味。

莉莉的招牌菜是一道名为“海洋之心”的料理。这道菜的灵感来自于她小时候在海边度过的时光。每当她闭上眼睛，就能听到海浪拍打岸边的声音，感受到海风轻拂脸颊的温柔。

“海洋之心”的制作过程非常独特。莉莉会选用最新鲜的海鲜，搭配上特制的酱汁，再点缀以海洋植物，让整道菜充满了海洋的气息。每当顾客品尝这道菜时，他们仿佛能感受到海浪的涌动，听到海鸥的叫声，仿佛置身于一个宁静的海边小镇。

随着时间的推移，“味觉之旅”的名声越来越响。然而，莉莉并没有因此而骄傲自满，她始终保持着谦逊和热情，用心为每一位顾客服务。

故事的结尾，是在莉莉的小店举办的一场名为 “海洋的馈赠”的晚宴。在这个特别的夜晚，莉莉邀请了所有曾经光顾过“味觉之旅”的顾客，共同庆祝她的美食之旅。晚宴上，“海洋之心”让所有人再次感受到了海洋的魔力。

当夜幕降临，星光点点，海风轻拂，人们围坐在小店的庭院里，享受着美食，分享着彼此的故事。在这个繁华的大城市中，莉莉用她的美食和热情，创造了一个属于每个人的海洋天堂。

**English translation**

In a bustling metropolis, a busy street teems with a crowd that surges like the tide. Along the street, neon lights flicker, and skyscrapers soar into the clouds. In such a vibrant place, there is a quiet and mysterious alley.

At the end of the alley, there is an inconspicuous little shop called "Journey of Taste". The owner of this shop is a young woman named Lily. She possesses a special ability—to create delicacies that leave a lasting impression on the palate. As night falls, people flock to this place in search of that unique flavor.

Lily's signature dish is a dish called "Heart of the Ocean". The inspiration for this dish comes from her childhood days spent by the seaside. Every time she closes her eyes, she can hear the sound of the waves crashing against the shore and feel the gentle caress of the sea breeze on her cheeks.

The process of making "Heart of the Ocean" is very unique. Lily selects the freshest seafood, pairs it with a specially made sauce, and adorns it with marine plants, filling the entire dish with the essence of the ocean. When customers taste this dish, they seem to feel the surge of the waves and hear the cries of seagulls, as if they were in a tranquil seaside town.

Over time, the reputation of "Journey of Taste" has grown louder and louder. However, Lily has not become arrogant or complacent because of this; she always maintains her humility and enthusiasm, serving every customer with her heart.

The end of the story is a dinner party called "Gifts of the Ocean" held at Lily's shop. On this special night, Lily invited all the customers who have ever visited "Journey of Taste" to celebrate her culinary journey together. At the dinner, "Heart of the Ocean" once again made everyone feel the magic of the ocean.

As night falls, with the stars twinkling and the sea breeze gently blowing, people sit around the courtyard of the shop, enjoying the food and sharing their stories. In this bustling metropolis, Lily has created an ocean paradise for everyone with her delicious food and warm hospitality.
