# Supplementary material for: The Effects of (Dis)similarities Between the Creator and the Assessor on Assessing Creativity: A Comparison of Humans and LLMs
Source: J Intell. 2025 Jul 3;13(7):80. doi: 10.3390/jintelligence13070080 (PMC12295035; doi:10.3390/jintelligence13070080)
Supplement: Supplementary file 1 [file jintelligence-13-00080-s001.zip › Supplementary Folder/Stage 1 - Story Collection/Originally Collected Stories/Chinese AI - Kimi/Story 10 - Non-creative.pdf]

Chinese original version

在一座繁华的大城市中，街道上总是熙熙攘攘，人们匆匆忙忙地穿梭于高楼大厦之间。城市的中心，有一家小餐馆，它坐落在一条繁忙的街道旁，尽管门面不起眼，但这里却有着城市中最好的美食。

这家餐馆的老板是一个中年男子，名叫李明。他年轻时曾在世界各地旅行，品尝了各种美食，最终决定将这些美味带回自己的家乡。餐馆的菜单上，有一道招牌菜——“世界融合炒饭”，它结合了亚洲和欧洲的风味，每一口都是对味觉的极致挑战。

一天，李明在忙碌中接到了一个电话，是他的老朋友，一位海洋生物学家。朋友告诉他，他即将启程前往一个遥远的海岛进行研究，希望李明能为他准备一些食物，以备在海岛上的长期研究。

李明立刻开始准备，他精心挑选了各种食材，制作了多份“世界融合炒饭”。他知道，这些食物不仅能满足朋友的味蕾，还能让他在远离家乡的海岛上感受到一丝家的温暖。

几周后，李明收到了朋友从海岛寄来的信。信中，朋友描述了他在海岛上的生活，以及他如何与岛上的居民分享了李明的炒饭。信的最后，朋友提到了海岛的美丽景色，以及海洋的壮丽和宁静。

李明被朋友的信深深触动，他决定在餐馆中增加一道新菜——“海洋之味”。这道菜以海鲜为主，融合了海洋的清新与炒饭的丰富口感。每当夜幕降临，城市的灯光映照在繁忙的街道上，人们在李明的餐馆中品尝着“海洋之味”，仿佛能感受到远方海岛的海风和海浪的声音。

English translation

In a bustling metropolis, the streets are always teeming with people, who hurry to and fro among the skyscrapers. At the heart of the city, there is a small restaurant situated next to a busy street. Despite its unassuming facade, it boasts the finest cuisine in the city.

The owner of the restaurant is a middle-aged man named Li Ming. In his youth, he traveled the world, savoring a variety of delicacies, and ultimately decided to bring these delicious flavors back to his hometown. On the restaurant's menu, there is a signature dish – "Global Fusion Fried Rice," which combines the flavors of Asia and Europe, offering an ultimate challenge to the taste buds with every bite.

One day, amidst the hustle and bustle, Li Ming received a phone call from his old friend, a marine biologist. His friend told him that he was about to embark on a research trip to a remote island and hoped Li Ming could prepare some food for his long-term research there.

Li Ming immediately began preparations. He carefully selected a variety of ingredients and made multiple servings of "Global Fusion Fried Rice." He knew that these dishes would not only satisfy his friend's palate but also bring a touch of homely warmth to him on the distant island.

A few weeks later, Li Ming received a letter from his friend on the island. In the letter, his friend described his life on the island and how he shared Li Ming's fried rice with the island's inhabitants. At the end of the letter, his friend mentioned the beautiful scenery of the island, as well as the grandeur and tranquility of the ocean.

Deeply moved by his friend's letter, Li Ming decided to add a new dish to his restaurant – "Taste of the Ocean." This dish, primarily seafood-based, blends the freshness of the ocean with the rich taste of fried rice. As night falls and the city lights reflect on the busy streets, people in Li Ming's restaurant taste the "Taste of the Ocean," as if they could feel the sea breeze and the sound of the waves from the distant island.
