# Supplementary material for: The Effects of (Dis)similarities Between the Creator and the Assessor on Assessing Creativity: A Comparison of Humans and LLMs
Source: J Intell. 2025 Jul 3;13(7):80. doi: 10.3390/jintelligence13070080 (PMC12295035; doi:10.3390/jintelligence13070080)
Supplement: Supplementary file 1 [file jintelligence-13-00080-s001.zip › Supplementary Folder/Stage 1 - Story Collection/Originally Collected Stories/Chinese AI - Kimi/Story 2 - Creative.pdf]

Chinese original version

在一座繁华的大城市中，街道总是熙熙攘攘。这条街道上，有一家不起眼的小餐馆，它的门面并不显眼，但里面却藏着一个秘密：这里的美食能让人忘记一切烦恼。

故事的主角是一位名叫艾米的年轻女孩，她是一名大学生，同时也是这家餐馆的常客。艾米总是被餐馆里的食物所吸引，特别是一道名为“海洋之梦”的神秘菜肴。这道菜的食材来自遥远的海洋，据说能够唤起人们对大海的无限遐想。

一天，艾米在餐馆里品尝着“海洋之梦”，突然间，她感到一阵眩晕。当她再次睁开眼睛时，发现自己竟然置身于一个奇妙的海底世界。周围的海水清澈见底，五彩斑斓的鱼儿在她身边游来游去，她能够感受到海水的温柔抚摸。

艾米惊讶地发现，她能够在水中自由呼吸，就像一条真正的美人鱼。她开始探索这个奇妙的世界，发现了一片片珊瑚礁，还有隐藏在海底的古老沉船。在沉船中，她找到了一本古老的日记，记录着一个关于海洋的传说。

传说中，海洋之神会将那些真心热爱海洋的人带到这个神秘的世界，让他们体验海洋的奇妙和美丽。艾米意识到，她之所以能够来到这里，是因为她对海洋的热爱和对美食的追求。

在海底世界度过了一段难忘的时光后被一股神秘的力量带回了现实。当她再次坐在餐馆里，她发现自己对海洋有了更深的理解和感情。她决定，要用知识和热情去保护这片美丽的海洋。

艾米成为了一名海洋保护者，激励着更多的人去关注和保护海洋。而那家小餐馆，也因为“海洋之梦”，成为了能够唤起人们对海洋无限遐想的地方。

English translation

In a bustling metropolis, the streets are always bustling with activity. On one such street, there is an inconspicuous small restaurant whose storefront is not eye-catching, but it hides a secret inside: the delicious food here can make people forget all their worries.

The protagonist of the story is a young girl named Amy, who is a college student and also a regular customer of the restaurant. Amy is always attracted by the food in the restaurant, especially a mysterious dish called "Ocean Dream." The ingredients of this dish come from the distant sea, and it is said to evoke people's boundless imagination about the ocean.

One day, Amy was tasting "Ocean Dream" in the restaurant when she suddenly felt dizzy. When she opened her eyes again, she found herself in a wonderful underwater world. The surrounding water was crystal clear, and colorful fish swam around her, and she could feel the gentle touch of the seawater.

Amy was surprised to find that she could breathe freely in the water, just like a real mermaid. She began to explore this wonderful world, discovering coral reefs and ancient sunken ships hidden on the seabed. In the sunken ship, she found an ancient diary, recording a legend about the ocean.

According to the legend, the sea god will bring those who truly love the ocean to this mysterious world, allowing them to experience the wonder and beauty of the ocean. Amy realized that she was able to come here because of her love for the ocean and her pursuit of delicious food.

After spending an unforgettable time in the underwater world, she was brought back to reality by a mysterious force. When she sat in the restaurant again, she found that she had a deeper understanding and affection for the ocean. She decided to use knowledge and passion to protect this beautiful ocean.

Amy became an ocean protector, inspiring more people to pay attention to and protect the ocean. And that small restaurant, also because of "Ocean Dream," became a place that can evoke people's boundless imagination about the ocean.
