# Supplementary material for: The Effects of (Dis)similarities Between the Creator and the Assessor on Assessing Creativity: A Comparison of Humans and LLMs
Source: J Intell. 2025 Jul 3;13(7):80. doi: 10.3390/jintelligence13070080 (PMC12295035; doi:10.3390/jintelligence13070080)
Supplement: Supplementary file 1 [file jintelligence-13-00080-s001.zip › Supplementary Folder/Stage 1 - Story Collection/Originally Collected Stories/Chinese AI - Kimi/Story 3 - Creative.pdf]

Chinese original version

在繁忙的都市中，艾米丽的小餐馆隐藏在一条安静的小巷里，与周围的喧嚣形成鲜明对比。这家餐馆的特别之处在于，艾米丽能够通过食物传递情感。

一天，艾米丽发现了一种来自遥远海洋的稀有鱼类，她决定用它制作一道特别的菜肴。夜幕降临，顾客们开始品尝这道独特的美食，感受着食物带来的温暖和安慰。

一位独自坐在角落的老人引起了艾米丽的注意。他的眼神中透露出深深的忧郁。艾米丽亲自为他服务，并轻声告诉他，这道菜能够唤起人们心中最真挚的情感。

老人品尝了一口鱼肉，眼中突然闪过一丝光芒。他开始讲述自己年轻时的故事，那是关于他和海洋的一段不解之缘。他曾是一名勇敢的船长，驾驶着自己的船只，探索过无数未知的海域。但随着时间的流逝，他失去了方向，也失去了对海洋的热爱。

艾米丽静静地听着老人的故事，她的心中充满了敬意和感动。她知道，她的食物不仅仅是满足味蕾的享受，更是一种情感的传递和治愈。在这个繁忙的大城市中，她的小餐馆成为了一个温暖的避风港，让人们在这里找到归属感和情感的慰藉。故事的结尾，老人站起身，向艾米丽鞠躬感谢，然后走出餐馆，仿佛重新踏上了探索海洋的旅程。艾米丽站在门口，望着远方的海洋，心中充满了对未来的期待和希望。她相信，她的美食和情感的力量，将会继续在这个大城市中传递下去，温暖更多的人。

English translation

In the bustling city, Emily's small restaurant is tucked away in a quiet alley, contrasting sharply with the surrounding noise. The uniqueness of this restaurant lies in Emily's ability to convey emotions through food.

One day, Emily discovered a rare fish from a distant ocean and decided to make a special dish with it. As night fell, customers began to taste this unique delicacy, feeling the warmth and comfort brought by the food.

An old man sitting alone in the corner caught Emily's attention. His eyes revealed deep melancholy. Emily personally served him and whispered that the dish could evoke the most sincere emotions in people's hearts.

The old man took a bite of the fish, and suddenly a glimmer appeared in his eyes. He began to tell his own story from his youth, which was about his indissoluble bond with the sea. He had been a brave captain, sailing his own ship and exploring countless unknown waters. But as time passed, he lost his direction and his love for the sea.

Emily listened quietly to the old man's story, her heart full of respect and emotion. She knew that her food was not only a pleasure for the taste buds but also a transmission and healing of

emotions. In this busy metropolis, her small restaurant had become a warm haven where people could find a sense of belonging and emotional comfort. At the end of the story, the old man stood up, bowed to Emily in gratitude, and then walked out of the restaurant as if embarking on a new journey to explore the sea. Emily stood at the door, looking at the distant sea, her heart full of expectations and hope for the future. She believed that her delicious food and the power of emotions would continue to be passed on in this big city, warming more people.
