# Supplementary material for: The Effects of (Dis)similarities Between the Creator and the Assessor on Assessing Creativity: A Comparison of Humans and LLMs
Source: J Intell. 2025 Jul 3;13(7):80. doi: 10.3390/jintelligence13070080 (PMC12295035; doi:10.3390/jintelligence13070080)
Supplement: Supplementary file 1 [file jintelligence-13-00080-s001.zip › Supplementary Folder/Stage 1 - Story Collection/Originally Collected Stories/Chinese AI - Kimi/Story 4 - Creative.pdf]

Chinese original version

在一座繁华都市的繁忙街道上，艾米，一位年轻女孩，每天穿梭在高楼大厦间，心中却怀揣着一个不为人知的梦想——探索海洋深处的奥秘。

一天，艾米在公司附近的小餐馆品尝到了一道海鲜烩饭，这道美味唤起了她对童年海边记忆的怀念。那一刻，她决定追寻自己的梦想。

艾米开始利用业余时间学习海洋学，她阅读了大量的书籍，参加了各种讲座和研讨会。她对海洋生物的多样性和海洋生态系统的复杂性产生了浓厚的兴趣。随着时间的推移，她的知识越来越丰富，她的技能也越来越娴熟。

终于，艾米得到了一个机会，她被选中参加一个深海探险队，去探索一个未知的海底世界。在准备出发的前夕，艾米再次来到了那家小餐馆，点了那道让她魂牵梦绕的海鲜烩饭。她知道，这可能是她在一段时间内最后一次品尝到陆地上的食物。

探险队出发了，艾米和她的队友们乘坐着先进的潜水艇，缓缓地潜入了深邃的海洋。随着深度的增加，光线逐渐消失，周围变得一片漆黑。但是，当潜水艇的灯光打开时，一个五彩斑斓的海底世界展现在他们面前。珊瑚礁、海草、各种奇异的海洋生物，这一切都让艾米感到无比的震撼和兴奋。

在这次探险中，艾米和队友们发现了一些前所未有的海洋生物，记录下了这些生物的特征，并采集了样本。这次探险不仅丰富了人类对海洋的认识，也让艾米的梦想得以实现。

探险结束后，艾米站在海边，望着无边的海洋，心中充满了感激和敬畏。她知道，海洋的奥秘远远超出了她的想象，而她的梦想，才刚刚开始。

English translation

In the bustling streets of a thriving metropolis, Amy, a young girl, navigates daily through the skyscrapers, yet she harbors an unknown dream in her heart—to explore the mysteries of the deep ocean.

One day, Amy tasted a seafood paella at a small restaurant near her company, and the delicious dish awakened her nostalgia for childhood memories by the sea. At that moment, she decided to pursue her dream.

Amy began to study oceanography in her spare time. She read numerous books and attended various lectures and seminars. She developed a keen interest in the diversity of marine life and the complexity of marine ecosystems. Over time, her knowledge grew more extensive, and her skills became more proficient.

Finally, Amy was given an opportunity; she was selected to join a deep-sea exploration team to discover an unknown underwater world. On the eve of their departure, Amy returned to the small restaurant and ordered the seafood paella that had haunted her dreams. She knew it might be the last time she would taste food from the land for a while.

The exploration team set off, and Amy and her teammates boarded an advanced submarine, slowly descending into the profound ocean. As they descended, light gradually faded, and the surroundings turned pitch black. However, when the submarine's lights were turned on, a colorful underwater world was revealed before them. Coral reefs, sea grass, and various strange marine creatures all left Amy feeling extremely shocked and excited.

During this expedition, Amy and her teammates discovered some previously unknown marine creatures, recorded their characteristics, and collected samples. The expedition not only enriched human understanding of the ocean but also fulfilled Amy's dream.

After the expedition, Amy stood by the sea, gazing at the boundless ocean, her heart full of gratitude and awe. She knew that the mysteries of the ocean far exceeded her imagination, and her dream had just begun.
