# Supplementary material for: The Effects of (Dis)similarities Between the Creator and the Assessor on Assessing Creativity: A Comparison of Humans and LLMs
Source: J Intell. 2025 Jul 3;13(7):80. doi: 10.3390/jintelligence13070080 (PMC12295035; doi:10.3390/jintelligence13070080)
Supplement: Supplementary file 1 [file jintelligence-13-00080-s001.zip › Supplementary Folder/Stage 1 - Story Collection/Originally Collected Stories/Chinese AI - Kimi/Story 5 - Creative.pdf]

Chinese original version

在一座繁华的都市中，街道上熙熙攘攘，行人匆匆，车辆川流不息。在这喧嚣之中，有一家不起眼的小店。这家店的主人是一位年迈的老人，总是穿着一件褪色的围裙，脸上带着慈祥的微笑。

老人的小店以一种祖传的独特的美食闻名：用古老配方制作的糕点。这种糕点不仅味道美妙，而且据说能够唤起人们内心深处最温暖的记忆。

随着时间的流逝，老人的小店逐渐成为了城市中的一个传说。人们从四面八方赶来，只为一尝那传说中的美味。然而，老人并不急于扩大生意，他总是说："真正的美食，不在于数量，而在于那份心意。"

在一个风和日丽的下午。一位年轻的艺术家走进了这家小店。艺术家品尝了糕点后，内心涌起了一股暖流，他的灵感如同潮水般涌现。他决定以这家小店和老人为灵感，创作一幅画作。

数月后，这幅画作在城市中引起了轰动。它不仅捕捉了小店的温馨氛围，还描绘了老人制作糕点时的专注和对美食的热爱。画作的展出吸引了更多人来到这家小店，人们开始理解，真正的幸福和满足，往往隐藏在生活的简单之处。

故事的结尾，老人决定将小店搬到海边。他相信，海洋的广阔和深邃能够给人们带来更多的灵感和平静。小店的新址位于一片宁静的海滩上，每当夕阳西下，金色的阳光洒在海面上，小店的灯光在海风中摇曳生辉。人们在这里品尝着糕点，聆听着海浪的声音，感受着生活的美好。老人微笑着，他知道，他的小店和糕点，已经成为了这座城市中一个永恒的传说。

English translation

In a bustling city, the streets are teeming with people, pedestrians hurry by, and traffic flows incessantly. Amidst the hustle and bustle, there is an inconspicuous little shop. The owner of this shop is an elderly person, always wearing a faded apron and with a kind smile on their face.

The old man's shop is famous for a unique, family-inherited delicacy: pastries made from an ancient recipe. These pastries are not only delicious but are said to evoke the warmest memories deep within people's hearts.

As time goes by, the old man's shop gradually becomes a legend in the city. People come from all directions just to taste the legendary delicacy. However, the old man is not eager to expand his business; he always says, "True delicacies are not about quantity, but about the intention behind them."

On a sunny afternoon, a young artist walked into the shop. After tasting the pastries, a warm current surged in the artist's heart, and his inspiration flowed like a tide. He decided to take the shop and the old man as inspiration for a painting.

Several months later, the painting caused a sensation in the city. It not only captured the cozy atmosphere of the shop but also depicted the old man's concentration and love for the delicacies when making the pastries. The exhibition of the painting attracted more people to the shop, and people began to understand that true happiness and satisfaction often hide in the simplicity of life.

At the end of the story, the old man decided to move the shop to the seaside. He believed that the vastness and depth of the ocean could bring more inspiration and tranquility to people. The new location of the shop is on a peaceful beach. As the sun sets, golden sunlight shines on the sea, and the light of the shop flickers in the sea breeze. People taste the pastries here, listen to the sound of the waves, and feel the beauty of life. The old man smiles, knowing that his shop and pastries have become an eternal legend in this city.
