# Supplementary material for: The Effects of (Dis)similarities Between the Creator and the Assessor on Assessing Creativity: A Comparison of Humans and LLMs
Source: J Intell. 2025 Jul 3;13(7):80. doi: 10.3390/jintelligence13070080 (PMC12295035; doi:10.3390/jintelligence13070080)
Supplement: Supplementary file 1 [file jintelligence-13-00080-s001.zip › Supplementary Folder/Stage 1 - Story Collection/Originally Collected Stories/Chinese AI - Kimi/Story 6 - Non-creative.pdf]

Chinese original version

在繁华的大城市中，街道总是熙熙攘攘，人来人往。我，一名普通的大学生，就生活在这个充满活力的城市里。每天，我穿梭在高楼大厦之间，感受着城市的脉动。街道两旁，霓虹灯闪烁，广告牌上的明星笑容灿烂，仿佛在告诉每一个路过的人，这里充满了无限可能。

一天，我像往常一样，从图书馆走出来，准备回宿舍。突然，一阵诱人的香味吸引了我。我顺着香味走去，发现是一家新开的小吃店。门口的招牌上写着“家乡味道”，我忍不住走进去，点了一份招牌菜——红烧肉。食物上桌的那一刻，我仿佛回到了童年，那熟悉的味道让我感到无比温暖和满足。那一刻，我忘记了学业的压力，忘记了城市的喧嚣，只有食物带来的简单快乐。

随着时间的流逝，大学生活即将结束。毕业典礼那天，我站在海边，望着无边的海洋，心中充满了感慨。海浪轻轻拍打着岸边，海风带着咸咸的味道，我闭上眼睛，深呼吸，感受着大自然的洗礼。海洋，这个广阔的世界，就像我即将踏入的社会，充满未知和挑战。

我想起了那家小吃店的红烧肉，那是我在这座城市中最温暖的回忆。也许，未来的某一天，我还会回到这里，再次品尝那份熟悉的味道。但此刻，我站在海边，心中充满了对未来的憧憬和希望。海洋，就像我即将启航的新旅程，虽然未知，但我相信，只要勇敢地迈出第一步，就能找到属于自己的方向。

English translation

In the bustling metropolis, the streets are always bustling with people coming and going. I, an ordinary college student, live in this vibrant city. Every day, I move through the skyscrapers, feeling the pulse of the city. On both sides of the street, neon lights flicker, and the stars on the billboards smile brightly, as if telling every passerby that there is infinite possibility here.

One day, as usual, I walked out of the library, preparing to return to the dormitory. Suddenly, an enticing aroma attracted me. I followed the scent and found a newly opened snack shop. The signboard at the entrance read "Home Town Flavor". I couldn't resist going in and ordered a signature dish - braised pork belly. The moment the food was served, I felt as if I had returned to my childhood. The familiar taste made me feel incredibly warm and satisfied. At that

moment, I forgot the pressure of my studies and the hustle and bustle of the city, only enjoying the simple happiness brought by the food.

As time passed, college life was about to end. On the day of the graduation ceremony, I stood by the sea, looking at the boundless ocean, full of emotion. The waves gently hit the shore, and the sea breeze carried a salty taste. I closed my eyes, took a deep breath, and felt the baptism of nature. The ocean, this vast world, is like the society I am about to step into, full of unknowns and challenges.

I thought of the braised pork belly from that snack shop, which is the warmest memory I have in this city. Perhaps, one day in the future, I will come back here and taste that familiar flavor again. But at this moment, I stand by the sea, my heart full of longing and hope for the future. The ocean is like a new journey I am about to embark on, although unknown, but I believe that as long as I bravely take the first step, I can find my own direction.
