# Supplementary material for: The Effects of (Dis)similarities Between the Creator and the Assessor on Assessing Creativity: A Comparison of Humans and LLMs
Source: J Intell. 2025 Jul 3;13(7):80. doi: 10.3390/jintelligence13070080 (PMC12295035; doi:10.3390/jintelligence13070080)
Supplement: Supplementary file 1 [file jintelligence-13-00080-s001.zip › Supplementary Folder/Stage 1 - Story Collection/Originally Collected Stories/Chinese AI - Kimi/Story 7 - Non-creative.pdf]

Chinese original version

在一个阳光明媚的早晨，他像往常一样穿梭在这座大城市的繁忙街道上，人潮涌动，车水马龙。高楼大厦间，他仿佛只是一粒微不足道的尘埃，但今天，他的心情却格外的轻松愉快。他是一名大学学生，今天没有课，他计划去图书馆度过一个宁静的下午。

走在街道上，他被一家小餐馆的香味所吸引。透过橱窗，他看到了一盘盘色香味俱佳的佳肴，他的肚子不由自主地发出了抗议。他决定走进去，点了一份招牌菜——香辣烤鱼。鱼肉外焦里嫩，辣味恰到好处，每一口都是满足。这家餐馆虽小，但食物却让人回味无穷，他心想，这大概是他今天最大的惊喜了。

饱餐一顿后，他继续前往图书馆。在那里，他沉浸在知识的海洋中，时间仿佛在书页间悄悄流逝。直到夕阳西下，他才意识到一天即将结束。他收拾好书包，走出图书馆，准备回家。

走在回家的路上，他经过了一片开阔的公园，那里有一片人工湖，湖水在夕阳的映照下波光粼粼，宛如一片小小的海洋。他坐在湖边的长椅上，看着湖面上的倒影，心中涌起一股莫名的感动。他想，尽管这座城市喧嚣而忙碌，但它也有它宁静而美丽的一面，就像这片湖，就像那家小餐馆的美味佳肴。

夜幕降临，他站起身，继续他的归途。今天，他在这个大城市中找到了属于自己的小确幸，也许这就是生活的魅力所在——在平凡中发现不平凡，就像海洋一样，表面平静，却蕴藏着无限可能。

English translation

On a sunny morning, he traversed the bustling streets of the big city as usual, amidst the surging crowds and the ceaseless flow of traffic. Amidst the towering skyscrapers, he seemed like an insignificant speck of dust, but today, his mood was particularly relaxed and joyful. He was a university student, with no classes today, and he planned to spend a peaceful afternoon in the library.

Walking on the street, he was drawn by the aroma wafting from a small restaurant. Through the window, he saw plates of delectable dishes that were appealing in color, aroma, and taste, and his stomach involuntarily protested. He decided to go in and ordered the signature dish—spicy roasted fish. The fish was crispy on the outside and tender on the inside, with just the right amount of spiciness, and every bite was satisfying. Though the restaurant was small, the

food was so delicious that it left a lasting impression. He thought to himself that this was probably the biggest surprise of his day.

After a satisfying meal, he continued on his way to the library. There, he immersed himself in the ocean of knowledge, with time slipping quietly between the pages of the books. It wasn't until the sun began to set that he realized the day was coming to an end. He packed up his bag, left the library, and prepared to go home.

On his way home, he passed through an open park with an artificial lake. The lake shimmered under the setting sun, resembling a small sea. He sat on a bench by the lake, watching the reflections on the water, and felt an inexplicable emotion welling up inside him. He thought that despite the city's noise and busyness, it also had its tranquil and beautiful side, just like this lake, just like the delicious food at the small restaurant.

As night fell, he stood up and continued his journey home. Today, he found his own small happiness in this big city. Perhaps this is the charm of life—to discover the extraordinary in the ordinary, like the ocean, calm on the surface but containing infinite possibilities.
