# Supplementary material for: The Effects of (Dis)similarities Between the Creator and the Assessor on Assessing Creativity: A Comparison of Humans and LLMs
Source: J Intell. 2025 Jul 3;13(7):80. doi: 10.3390/jintelligence13070080 (PMC12295035; doi:10.3390/jintelligence13070080)
Supplement: Supplementary file 1 [file jintelligence-13-00080-s001.zip › Supplementary Folder/Stage 1 - Story Collection/Originally Collected Stories/Chinese AI - Kimi/Story 8 - Non-creative.pdf]

Chinese original version

繁忙的大城市中，一条狭窄小巷里，李阿姨经营着一家小餐馆，与周围的喧嚣形成鲜明对比。餐馆虽不起眼，但李阿姨总是以真诚的笑容迎接每一位顾客，她的拿手好菜红烧肉，简单却充满家的味道。

小林，一个外地来的年轻人，偶然走进了这家餐馆。他刚到这座城市，对快节奏的生活感到不适应。在品尝了李阿姨的红烧肉后，他感到了久违的温暖和满足。李阿姨告诉他，尽管城市生活忙碌，但只要心中有爱，就能找到宁静。

随着时间的推移，小林逐渐适应了这座城市的生活，并且成为了这家餐馆的常客。他在这里结识了许多朋友，也在这里找到了自己的爱情。每当他感到疲惫或是迷茫时，他就会来到这里，品尝李阿姨的红烧肉，感受那份简单而纯粹的幸福。

多年后，小林已经是一名成功的律师，但他仍然会时不时地回到这家餐馆。这一次，他带着自己的家人来到这里，庆祝他儿子的毕业。李阿姨的餐馆依然如故，简单而温馨。小林的儿子也被这里的红烧肉深深吸引，他问李阿姨这道菜的秘诀是什么。李阿姨笑着说：“秘诀就是用心。”

离开餐馆后，小林一家人沿着海边散步。海风轻拂，海浪拍打着岸边，小林看着波光粼粼的海面，心中充满感激。他感谢这座城市，感谢那家小餐馆和李阿姨，让他在忙碌中找到了温暖和宁静，就像海洋，无论何时，都能给人带来平静和力量。

English translation

In the bustling metropolis, tucked away in a narrow alley, Aunt Li runs a small restaurant, a stark contrast to the surrounding hustle and bustle. The restaurant may not be eye-catching, but Aunt Li always greets every customer with a sincere smile. Her specialty, braised pork belly, is simple yet full of the taste of home.

Xiao Lin, a young man from out of town, accidentally walked into this restaurant. He had just arrived in the city and was not used to the fast-paced life. After tasting Aunt Li's braised pork belly, he felt a long-lost warmth and satisfaction. Aunt Li told him that although city life is busy, as long as there is love in the heart, one can find peace.

As time passed, Xiao Lin gradually adapted to the life of the city and became a regular customer at the restaurant. He made many friends here and found his own love. Whenever he

felt tired or confused, he would come here to taste Aunt Li's braised pork belly and feel that simple and pure happiness.

Years later, Xiao Lin has become a successful lawyer, but he still returns to the restaurant from time to time. This time, he brought his family here to celebrate his son's graduation. Aunt Li's restaurant remains the same, simple and warm. Xiao Lin's son was also deeply attracted by the braised pork belly here, and he asked Aunt Li what the secret of the dish was. Aunt Li smiled and said, "The secret is to cook with heart."

After leaving the restaurant, Xiao Lin's family took a walk along the sea. The sea breeze was gentle, and the waves hit the shore. Xiao Lin looked at the sparkling sea surface, full of gratitude. He thanked the city, the small restaurant and Aunt Li, for allowing him to find warmth and peace in the busy life, just like the ocean, which can bring calmness and strength to people at any time.
