# Supplementary material for: The Effects of (Dis)similarities Between the Creator and the Assessor on Assessing Creativity: A Comparison of Humans and LLMs
Source: J Intell. 2025 Jul 3;13(7):80. doi: 10.3390/jintelligence13070080 (PMC12295035; doi:10.3390/jintelligence13070080)
Supplement: Supplementary file 1 [file jintelligence-13-00080-s001.zip › Supplementary Folder/Stage 1 - Story Collection/Originally Collected Stories/Chinese AI - Kimi/Story 9 - Non-creative.pdf]

Chinese original version

在一个繁华的大城市中，街道上人来人往，车辆川流不息。在这样一个充满活力的地方，有一个名叫李明的年轻人，他是一名普通的大学生，每天穿梭于校园和图书馆之间，过着看似平凡却又充满挑战的生活。

李明有一个梦想，那就是成为一名厨师，创造出能够触动人心的美食。尽管学业繁重，但他总是挤出时间来研究各种食谱。他的宿舍里总是弥漫着各种食材的香味，同学们也常常被他所做的美食所吸引。

一天，李明在图书馆的角落里发现了一本古老的食谱，上面记载着一种失传已久的美食。他决定尝试着复原这道佳肴。经过无数次的尝试和失败，他终于在一次偶然的机会中，成功地制作出了这道令人难以忘怀的美食。这道美食不仅味道独特，而且外观也极为精致。

消息很快在校园里传开，李明的美食吸引了越来越多的人前来品尝。他意识到，这不仅仅是一道菜，更是他实现梦想的开始。他开始在校园里举办小型的美食节，邀请同学们来品尝他的创作，并且得到了极高的评价。

随着时间的推移，李明的名声越来越大，他的美食节也逐渐成为了校园文化的一部分。毕业之际，他收到了一家知名餐厅的邀请，希望他能成为他们的主厨。

在毕业典礼的那天，李明站在海边，望着无垠的大海，心中充满了对未来的憧憬。他知道，自己的旅程才刚刚开始，就像那广阔的海洋，充满了无限的可能。他决定接受邀请，带着自己的梦想和对美食的热爱，踏上了新的旅程。而那本古老的食谱，也成了他永远的宝藏，陪伴他在美食的道路上不断探索和前行。

English translation

In a bustling metropolis, the streets are teeming with people and traffic flows incessantly. In such a vibrant place, there is a young man named Li Ming, an ordinary college student who commutes daily between campus and the library, leading a seemingly ordinary yet challenging life.

Li Ming has a dream, which is to become a chef and create dishes that can touch people's hearts. Despite the heavy academic workload, he always finds time to study various recipes. His dormitory is always filled with the aroma of different ingredients, and his classmates are often attracted by the delicious food he makes.

One day, Li Ming found an ancient cookbook in the corner of the library, which recorded a long-lost dish. He decided to try to restore this delicacy. After countless attempts and failures, he finally succeeded in making this unforgettable dish by chance. This dish is not only unique in taste but also exquisite in appearance.

The news quickly spread throughout the campus, and Li Ming's food attracted more and more people to taste it. He realized that this was not just a dish, but also the beginning of his dream. He began to hold small food festivals on campus, inviting classmates to taste his creations, and received very high praise.

As time went by, Li Ming's reputation grew larger and larger, and his food festival gradually became a part of campus culture. Upon graduation, he received an invitation from a well-known restaurant, hoping that he could become their head chef.

On the day of the graduation ceremony, Li Ming stood by the sea, looking at the boundless ocean, full of longing for the future. He knew that his journey had just begun, like the vast ocean, full of infinite possibilities. He decided to accept the invitation, with his dreams and love for food, embarking on a new journey. And that ancient cookbook has also become his eternal treasure, accompanying him to continue exploring and moving forward on the road of food.
