# Supplementary material for: The Effects of (Dis)similarities Between the Creator and the Assessor on Assessing Creativity: A Comparison of Humans and LLMs
Source: J Intell. 2025 Jul 3;13(7):80. doi: 10.3390/jintelligence13070080 (PMC12295035; doi:10.3390/jintelligence13070080)
Supplement: Supplementary file 1 [file jintelligence-13-00080-s001.zip › Supplementary Folder/Stage 1 - Story Collection/Originally Collected Stories/Chinese Human Participants/Story 1 Creative.pdf]

Chinese original version

上海市中心永远不缺人气，街道两旁商场和写字楼林立，行人们步履匆匆，仿佛每一分钟都不愿浪费。

一天傍晚，一个叫李佳的996打工人走进了这家书店。她是一名广告公司的设计师，忙碌的工作让她身心俱疲。她随意地浏览着书架上的书籍，突然被一本封面上画着精致手绘插图的书吸引住了。这本书叫做《品尝所见之物》，书中有大量的插图记录了世界各地的美食。李佳翻开书页，细细阅读起来。

随着她的阅读，她仿佛渐渐地能闻到书中美食图片散发出的香味，并且越来越明显、越来越浓烈。李佳抱着困惑但极度好奇的心情抱着书走进了书店的小角落，将鼻子凑近书中的图片。“真的是图片有味道！”李佳心想。大胆的她将手指戳向其中一张法国可颂的图片。神奇的事情发生了，本该是纸张的触感被松软且带着少许黄油的面包触感替代，李佳直接从书本的插图中捏出了一个完整的可颂。

大胆的她对着散发出黄油香气，有着金黄酥皮的可颂大啃一口，并感叹道这竟然比上海大部分烘焙店的手艺都要好！接下来的一小时内李佳尝遍了书中所有漂亮的料理，尤其是海鲜类食物，因为在海滨城市长大的她最爱的就是那一口粉丝蒸扇贝。在书店闭店前，李佳拿着书准备去结账，却一不小心跟另外一位顾客撞了一下，手中拿着的东西都跌落在地。等李佳捡起地上的东西后却发现《品尝所见之物》消失了，可她吃撑了的胃却是真真切切的。也许这是老天给打工人的一份小礼物吧。

English translation

The heart of Shanghai is forever bustling with activity. Lining the streets are shopping malls and office buildings, and passersby hurry along as if they are unwilling to waste a single minute.

One evening, a 996 worker named Li Jia walked into a bookstore. She is a designer at an advertising company, and her busy job leaves her physically and mentally exhausted. She casually browsed the books on the shelves and was suddenly attracted by one with delicate hand-drawn illustrations on the cover. The book was called "Tasting What You See," which

contained a plethora of illustrations documenting delicacies from around the world. Li Jia opened the pages and began to read attentively.

As she read, she seemed to gradually be able to smell the fragrance of the food in the images, becoming more and more distinct and intense. With a puzzled but extremely curious mood, Li Jia held the book and walked into a small corner of the bookstore, bringing her nose close to the images in the book. "The pictures really do have a smell!" Li Jia thought. Boldly, she poked her finger at an image of a French croissant. A magical thing happened: what should have been the touch of paper was replaced by the soft and slightly buttery texture of bread, and Li Jia directly pinched out a complete croissant from the book's illustration.

Boldly, she took a big bite of the croissant, which exuded a buttery aroma and had golden flaky skin, and exclaimed that it was actually better than most bakeries in Shanghai! In the following hour, Li Jia tasted all the beautiful dishes in the book, especially seafood, because she grew up in a coastal city and her favorite was the steamed scallops with vermicelli. Before the bookstore closed, Li Jia was preparing to pay for the book when she accidentally bumped into another customer, and everything in her hand fell to the ground. After Li Jia picked up the things on the ground, she found that "Tasting What You See" had disappeared, but her bloated stomach was real. Perhaps this was a small gift from the heavens for the working class.
