# Supplementary material for: The Effects of (Dis)similarities Between the Creator and the Assessor on Assessing Creativity: A Comparison of Humans and LLMs
Source: J Intell. 2025 Jul 3;13(7):80. doi: 10.3390/jintelligence13070080 (PMC12295035; doi:10.3390/jintelligence13070080)
Supplement: Supplementary file 1 [file jintelligence-13-00080-s001.zip › Supplementary Folder/Stage 1 - Story Collection/Originally Collected Stories/Chinese Human Participants/Story 10 Non-Creative.pdf]

Chinese original version

在这个喧嚣的大都市，我每天都要忍受着拥挤和喧嚣，努力地在这座城市中生存。我的名字叫老王，是一名普通的中年男性，每天都在这座城市的喧嚣中寻找自己的出路。清晨，我从狭窄的小巷走出来，踏上了拥挤的街道。汽车的喇叭声、行人的嘈杂声、商店的招牌灯光，构成了这个城市特有的节奏。人来人往，我只是其中一个匆匆而过的身影，毫无特点，不会成为任何故事的主角。中午，我来到了一家小小的餐馆，点了一份家常菜。那是一家普普通通的小餐馆，装修简陋，却总是能散发出一股诱人的香味。我喜欢这里的食物，简单而美味，总能让我在疲惫的工作之余找到一丝慰藉。每当我品尝着那些熟悉的味道，都仿佛能感受到家的温暖。傍晚，我来到了海边，站在海滩上，我看着波涛汹涌的大海，心情不由得平静了许多。海风拂过脸庞，带来一丝清凉。我闭上眼睛，静静地聆听着海浪拍打在礁石上的声音，沉浸在海的宁静之中。回到家里，我躺在床上，回想着今天发生的种种。虽然生活平淡，但却充满了温馨和希望。在这个大都市里，我或许只是一个普通的中年男性，但我有我的家庭、我的工作，还有那些平凡而珍贵的细节，让我觉得生活是如此美好。在这个繁忙的城市中，我找到了自己的小确幸，也找到了生活的意义。

English translation

In this bustling metropolis, I have to endure the congestion and noise every day, striving to survive in this city. My name is Lao Wang, an ordinary middle-aged man, looking for my way out amidst the hustle and bustle of this city every day. In the early morning, I step out from the narrow alleys and onto the crowded streets. The honking of cars, the noise of pedestrians, and the neon lights of shop signs form the unique rhythm of this city. Among the comings and goings, I am just one of the hurried figures, unremarkable and not the protagonist of any story. At noon, I came to a small restaurant and ordered a home-cooked meal. It's an ordinary little restaurant with simple decoration, but it always exudes an enticing aroma. I like the food here, simple and delicious, always able to find a bit of comfort for me after the tiring work. Every time I taste those familiar flavors, it feels like I can feel the warmth of home. In the evening, I came to the seaside, standing on the beach, I watched the surging waves of the sea, and my mood couldn't help but calm down. The sea breeze brushed across my face, bringing a bit of coolness. I closed my eyes, quietly listening to the sound of the waves hitting the rocks, immersed in the tranquility of the sea. Back home, I lay on the bed, recalling the events of

today. Although life is plain, it is full of warmth and hope. In this metropolis, I may just be an ordinary middle-aged man, but I have my family, my job, and those ordinary yet precious details, which make me feel that life is so beautiful. In this busy city, I have found my own small happiness and the meaning of life.
