# Supplementary material for: The Effects of (Dis)similarities Between the Creator and the Assessor on Assessing Creativity: A Comparison of Humans and LLMs
Source: J Intell. 2025 Jul 3;13(7):80. doi: 10.3390/jintelligence13070080 (PMC12295035; doi:10.3390/jintelligence13070080)
Supplement: Supplementary file 1 [file jintelligence-13-00080-s001.zip › Supplementary Folder/Stage 1 - Story Collection/Originally Collected Stories/Chinese Human Participants/Story 2 Creative.pdf]

Chinese original version

《城市与海的邂逅》

在大城市繁忙街道的车水马龙中，灯光闪烁，行人匆匆。啵啵挤在熙熙攘攘的人群里，耳边是各种嘈杂的声音，汽车的喇叭声、商店的音乐声和人们的交谈声交织在一起。她刚刚结束了一场重要的会议，身心俱疲。

走着走着，啵啵被一股浓郁的香味吸引，她顺着香味拐进了一条小巷，发现了一家不起眼但热闹非凡的小吃店。店里的各种美食让她垂涎欲滴，她点了一份特色的海鲜煎饼和一碗热气腾腾的汤羹。当她咬下第一口海鲜煎饼时，那美妙的滋味在口中散开，鲜嫩的海鲜与酥脆的饼皮完美结合，让她的味蕾沉浸在幸福之中。

享用完好吃的食物后，啵啵感觉自己重新充满了能量。她信步走着，不知不觉来到了城市的边缘，眼前豁然开朗，一片无垠的海洋出现在她的面前。她静静地站在沙滩上，海风轻柔地抚摸着她的脸庞。海浪一波又一波地拍打着海岸，发出阵阵声响。那广阔的海洋仿佛有着一种神奇的魔力，让她的内心变得无比平静。她看着远处的海平面，思绪也渐渐飘远。那些在大城市中积累的压力和烦恼，在这一刻都被海洋的浩瀚所吞没。她明白了，生活不仅仅只有城市的繁忙与喧嚣，还有这宁静而美丽的海洋等待着她去感受和领悟。在这片海洋面前，她觉得自己是如此渺小，而世界又是如此广阔，充满着无限的可能和希望。

English translation

"Encounter of the City and the Sea"

In the bustling streets of the metropolis, traffic flows like a river, with the flickering lights and the hurried footsteps of passersby. Amidst the throng of people, BoBo is squeezed in, her ears filled with a cacophony of sounds—the honking of cars, the music from shops, and the chatter of people all intermingling. She has just finished an important meeting and is both physically and mentally exhausted.

As she walks, BoBo is drawn by a rich aroma. She follows her nose and turns into a small alley, discovering a modest yet bustling snack shop. The array of delicacies inside makes her mouth

water, and she orders a plate of the shop's signature seafood pancake and a bowl of steaming soup. When she takes the first bite of the seafood pancake, the delightful flavors burst in her mouth—the tender seafood perfectly complemented by the crispy crust, immersing her taste buds in bliss.

After savoring the delicious meal, BoBo feels reenergized. She strolls along, and before she knows it, she finds herself at the edge of the city. Suddenly, her view opens up to reveal the vast expanse of the ocean. She stands quietly on the beach, with the gentle sea breeze caressing her face. Waves crash against the shore one after another, creating a rhythmic sound. The vast sea seems to possess a magical power, bringing an immense tranquility to her heart. As she gazes at the distant horizon, her thoughts begin to wander. The pressures and worries accumulated in the city are, at this moment, swallowed by the vastness of the ocean. She realizes that life is not only about the hustle and bustle of the city but also about the tranquility and beauty of the sea, waiting for her to experience and understand. In the presence of this ocean, she feels so small, yet the world is so vast, filled with endless possibilities and hope.
