# Supplementary material for: The Effects of (Dis)similarities Between the Creator and the Assessor on Assessing Creativity: A Comparison of Humans and LLMs
Source: J Intell. 2025 Jul 3;13(7):80. doi: 10.3390/jintelligence13070080 (PMC12295035; doi:10.3390/jintelligence13070080)
Supplement: Supplementary file 1 [file jintelligence-13-00080-s001.zip › Supplementary Folder/Stage 1 - Story Collection/Originally Collected Stories/Chinese Human Participants/Story 3 Creative.pdf]

Chinese original version

在北京这个大都市的繁忙街道上，林小羽每天早晨都在川流不息的人群中匆匆赶往学校上课。有一天，她路过一个新开的早餐店，被一股浓郁的香味吸引，停下来买了一块摊主推荐的海苔肉松饼。饼子外酥里嫩，海苔和肉松的味道完美融合，让她惊艳过后一试难忘。从那天起，她每天都会去买这饼子，并与摊主成了朋友。摊主是一位上了年纪的老人，他告诉小羽，这种饼子是他多年实验的成果，希望能带给人们更多的美食快乐。小羽被老人的执着和热情感动，决定利用暑假把这种美味介绍给更多人。她和朋友们筹集资金，在几个大城市开了分店，海苔肉松饼迅速受到欢迎。为了庆祝成功，小羽和朋友们决定去海边度假。当他们站在海岸边，感受着海风和浪花时，小羽突发奇想：“为什么不在海边开一家餐馆，结合海洋元素，创造更多独特的美食呢？”朋友们纷纷表示赞同。他们开始了新的旅程，在海边开了一家以海洋为主题的餐馆。除了老人的海苔肉松饼，餐馆还推出了许多海洋风味的创新菜肴，如海鲜炒饭、海带凉菜等。餐馆一开业便吸引了大量顾客，成为当地的热门打卡点。站在海边，望着无尽的海洋，小羽心中充满了成就感。她知道，这片大海不仅是他们美食旅程的新起点，也是他们梦想继续扬帆远航的地方。

English translation

In the busy streets of the metropolis of Beijing, Lin Xiaoyu rushes through the endless streams of people every morning to get to school for her classes. One day, as she passed by a newly opened breakfast shop, she was drawn in by a rich aroma and stopped to buy a piece of the seaweed and pork floss cake recommended by the stall owner. The cake was crispy on the outside and tender on the inside, with the flavors of seaweed and pork floss perfectly blended, leaving her with an unforgettable experience after the first taste. From that day on, she made it a daily routine to buy the cake and became friends with the stall owner, an elderly man who told Xiaoyu that the cake was the result of many years of experimentation, hoping to bring more culinary joy to people. Touched by the old man's dedication and passion, Xiaoyu decided to introduce this delicious treat to more people during the summer vacation. She and her friends raised funds and opened branches in several major cities, and the seaweed and pork floss cake quickly gained popularity.

To celebrate their success, Xiaoyu and her friends decided to go on a beach vacation. As they stood by the coast, feeling the sea breeze and the waves, Xiaoyu suddenly had an idea: "Why

not open a restaurant by the sea, combining marine elements to create more unique dishes?" Her friends all agreed. They embarked on a new journey and opened a sea-themed restaurant by the sea. In addition to the old man's seaweed and pork floss cake, the restaurant also introduced many innovative dishes with a marine flavor, such as seafood fried rice and kelp cold dishes. The restaurant attracted a large number of customers as soon as it opened and became a popular spot in the area.

Standing by the sea, looking at the endless ocean, Xiaoyu was filled with a sense of achievement. She knew that this vast sea was not only the starting point of their culinary journey but also the place where their dreams would continue to set sail.
