# Supplementary material for: The Effects of (Dis)similarities Between the Creator and the Assessor on Assessing Creativity: A Comparison of Humans and LLMs
Source: J Intell. 2025 Jul 3;13(7):80. doi: 10.3390/jintelligence13070080 (PMC12295035; doi:10.3390/jintelligence13070080)
Supplement: Supplementary file 1 [file jintelligence-13-00080-s001.zip › Supplementary Folder/Stage 1 - Story Collection/Originally Collected Stories/Chinese Human Participants/Story 4 Creative.pdf]

Chinese original version

作为一名从小就非常喜欢日本动漫作品的资深宅男，小斌一直的梦想就是去到日本感受被奇妙二次元气息环绕的感觉。所以高中毕业后的小斌花了一个月时间疯狂的打工，一天做10份不同的工作，攒下了一笔可观的旅游资金，踏上了前往日本的航班。到达后小斌丝毫不休息，急忙赶去了新宿那所谓世界上最繁华的十字路口，该地区的附近有非常多适合二次元宅男打卡的商店。

站在十字路口中央的小斌一时间忘记了自己还身处在车流量繁忙的马路上，只听砰的一声，再次睁开眼，小斌发现自己竟然从都市的场景跳跃到茫茫的大海中的一艘船上。没等小斌仔细思考到底发生了什么他意识到这艘船正是他最喜欢的动漫海贼王里面男主路飞和小伙伴们开的那艘。而故事的主角路飞站在船头看向小斌并说：欢迎你的加入，新的伙伴啊！

就这样，在接下来的一个星期里小斌跟着路飞一起冒险一起品尝只有在动漫里才会出现的稀奇古怪的美食，包括那比脸还要大的排骨。某一天的任务路飞找到了一颗恶魔果实，并对小斌说你吃下这颗果实吧，它可以实现你最想实现的一个愿望。可是你不能做选择果实会自动找到你潜意识里最渴望得到的东西，并且实现它。

小斌满怀期待的吞下了这颗恶魔果实，闭上眼再次醒来发现自己回到了新宿的十字路口。原来，小斌内心最渴望的并不是得到什么超能力，也并不是留在异世界的海洋上继续那天马行空的冒险，而是回到他所真正属于的世界。

English translation

As a seasoned homebody who has been fond of Japanese anime since childhood, Xiao Bin has always dreamed of visiting Japan to experience the magical atmosphere of the two-dimensional world. Therefore, after graduating from high school, Xiao Bin spent a month working frantically, holding down 10 different jobs a day, saving up a substantial amount of travel funds, and boarding a flight to Japan. Upon arrival, Xiao Bin did not rest at all and hurried to the Shinjuku intersection, known as the most bustling in the world, where there are many stores suitable for otaku to check in.

Standing in the middle of the intersection, Xiao Bin momentarily forgot that he was still on a busy road. Suddenly, with a bang, he opened his eyes again to find himself on a ship in the vast ocean, having jumped from the urban scene. Before Xiao Bin had time to think about what had happened, he realized that this was the same ship from his favorite anime, One Piece, where the protagonist Luffy and his crew sailed. Luffy, standing at the bow of the ship, looked at Xiao Bin and said: "Welcome aboard, new partner!"

In the following week, Xiao Bin embarked on adventures with Luffy, tasting bizarre and exotic foods that only appeared in anime, including a huge slab of ribs that was bigger than one's face. One day, during a mission, Luffy found a Devil Fruit and told Xiao Bin to eat it, saying it could fulfill his greatest wish. However, Xiao Bin could not choose; the fruit would automatically find what he subconsciously desired the most and grant it.

Filled with anticipation, Xiao Bin swallowed the Devil Fruit, closed his eyes, and when he woke up again, he found himself back at the Shinjuku intersection. It turned out that what Xiao Bin truly desired the most was not superpowers, nor was it to continue the fantastical adventures on the ocean of another world, but to return to the world where he truly belonged.
