# Supplementary material for: The Effects of (Dis)similarities Between the Creator and the Assessor on Assessing Creativity: A Comparison of Humans and LLMs
Source: J Intell. 2025 Jul 3;13(7):80. doi: 10.3390/jintelligence13070080 (PMC12295035; doi:10.3390/jintelligence13070080)
Supplement: Supplementary file 1 [file jintelligence-13-00080-s001.zip › Supplementary Folder/Stage 1 - Story Collection/Originally Collected Stories/Chinese Human Participants/Story 5 Creative.pdf]

Chinese original version

很多人都面临着同样的选择，留在宽敞人少的农村呢，还是去往繁华拥挤的城市呢？也许有人会说大家都是成年人了，不能都选吗？可现实很残酷，谁都不是哆啦A梦。无论多么想念乡村的清新的空气和锅气十足的农家菜，为了更高的收入和更光明的未来，美美还是选择在北京发展，即使这意味着她需要忍受拥挤堵塞的交通和排队一个小时才能吃到的连锁店的预制菜。

也不知是不是因为美美对农家菜的渴望到达了一定的阀值，这一天当她推开家门的时候，他发现本该是客厅的地方，变成了一片田野。田野远处正在劳作的人可不就是美美的父亲吗？大步奔向父亲，美美和父亲都很吃惊为何会突然相见。

作为吃货的美美把神奇的门一事忘在脑后，先将自己对醉鹅的渴望表达了出来。父亲立马抓住自家饲养的大额交给了母亲。在用柴火烧出的大火，大锅烹饪翻炒之后，香气扑鼻的醉鹅做好了。饥肠辘辘的美美一个人就吃完了一整只鹅，这时她才想起连接着自己家等的上门还在开着呢！万一进贼了可怎么办！于是她狂奔着回到了门的地方。

再次拉开门把手出现在眼前的却是沙滩和汪洋大海，和远处客满为患的海鲜烧烤店。经过门的那一刹那，美美肚子里的大鹅仿佛瞬间消化完了。“还回什么家呢？继续吃吧。”就这样美美开始了一段神奇的美食之旅。

English translation

Many people are faced with the same choice: to stay in the spacious and sparsely populated countryside, or to head to the bustling and crowded city? Some might say, "We are all adults now, can't we have both?" But reality is cruel, and no one is Doraemon. No matter how much one misses the fresh air of the countryside and the hearty home-cooked meals, for the sake of a higher income and a brighter future, Mei Mei still chose to develop in Beijing, even if it means enduring congested traffic and waiting in line for an hour just to eat the pre-made dishes from chain restaurants.

Perhaps Mei Mei's longing for country dishes had reached a certain threshold. One day, when she pushed open the door of her home, she found that what should have been the living room

had turned into a field. Isn't that her father working in the distance? Rushing towards her father, both Mei Mei and her father were surprised to see each other so suddenly.

As a foodie, Mei Mei forgot about the magical door and first expressed her desire for roast goose. Her father immediately handed over a large goose from their own farm to her mother. After cooking it in a big pot over a fire made with firewood, the fragrant roast goose was ready. Mei Mei, who was ravenous, ate an entire goose by herself. Only then did she remember that the door connecting to her home was still open! What if a thief had come in! So she ran back to the place where the door was.

Once again, when she pulled the door handle, what appeared in front of her was the beach and the vast ocean, and in the distance, a seafood barbecue restaurant crowded with customers. At the moment of passing through the door, it seemed as if the goose Mei Mei had eaten in her stomach had been digested instantly. "Why go home? Keep eating." In this way, Mei Mei began a magical journey of delicious food.
