# Supplementary material for: The Effects of (Dis)similarities Between the Creator and the Assessor on Assessing Creativity: A Comparison of Humans and LLMs
Source: J Intell. 2025 Jul 3;13(7):80. doi: 10.3390/jintelligence13070080 (PMC12295035; doi:10.3390/jintelligence13070080)
Supplement: Supplementary file 1 [file jintelligence-13-00080-s001.zip › Supplementary Folder/Stage 1 - Story Collection/Originally Collected Stories/Chinese Human Participants/Story 6 Non-Creative.pdf]

Chinese original version

大连从一大清早就开始车水马龙，和老马的家乡小县城完全不一样。学生和社畜们仿佛有用不完的精力，每天在不同的地点间穿梭着。沿海城市的清晨时常被雾气弥漫着，空气中飞舞的水雾使老马感到困倦。早起的人们的活力并不能驱散这些顽固的雾气，必须要等太阳和海风联手合作才能使能见度恢复。

老马在市中心的一所中学门口摆摊卖蚵仔煎，每天都要起老早准备食材。虽然一份赚不了多少利润，但是薄利多销，赚点外快支撑着老马自己一个人的退休生活还是不错的。老马更看重的其实是学生们带来的活力，每天看着这些孩子们蹦蹦跳跳欢声笑语，仿佛自己也年轻了几岁。

随着时间的推移，孩子们从最初对蚵仔煎爱不释手到渐渐腻歪，不那么时常光顾老马的小吃摊了，反而转去旁边陈妈的炸酱面店吃饭。看着推车上金黄酥鲜的蚵仔煎，老马陷入了沉思，难道他做出的食物跟他一样也会被岁月淘汰吗？

老马叫住一名往常的熟客学生询问，对方说：这么几个月了吃的好腻啊，再好吃也不想多吃了，总得换换口味吧。。。叔叔你就不能创新一点吗？

老马心想确实啊，大连有这么好的海鲜资源，广阔的海洋每天都给人们送来各种新鲜的食材，为什么不试着创造些新的美食呢？于是，老马开始尝试将生蚝替换成其他品种的小海鲜，并逐渐赢回了学生们的胃。他意识到年龄永远不是能将人打败的因素，一成不变才是。

English translation

Dalian has been bustling with traffic from early morning, which is completely different from the small county town where Old Ma is from. Students and office workers seem to have endless energy, shuttling between different places every day. The early morning of the coastal city is often shrouded in fog, and the water mist floating in the air makes Old Ma feel sleepy. The vitality of the early risers cannot disperse the stubborn fog; it has to wait for the sun and the sea breeze to work together to restore visibility.

Old Ma sets up a stall at the entrance of a middle school in the city center to sell oyster omelettes. He has to get up very early every day to prepare the ingredients. Although he doesn't make much profit from each serving, the small profit and large volume make it a good way to support his own retirement life. What Old Ma values more is the vitality brought by the students. Watching these children jump around and laugh every day, he feels as if he has become several years younger.

As time goes by, the children went from being infatuated with the oyster omelettes to gradually getting tired of them and not visiting Old Ma's snack stall as often, instead turning to Chen's mother's noodle shop next door for meals. Looking at the golden and crispy oyster omelettes on the cart, Old Ma fell into deep thought, could it be that the food he made would be eliminated by the years just like him?

Old Ma stopped a regular student customer and asked, and the other party said: It's been several months, I'm so tired of eating it, even if it's delicious, I don't want to eat it anymore, I have to change the taste... Uncle, can't you be more innovative?

Old Ma thought to himself, Dalian has such good seafood resources, and the vast sea sends people all kinds of fresh ingredients every day, why not try to create some new delicacies? So, Old Ma began to try replacing oysters with other varieties of small seafood and gradually won back the students' stomachs. He realized that age is never a factor that can defeat a person, it is the unchanging that is.
