# Supplementary material for: The Effects of (Dis)similarities Between the Creator and the Assessor on Assessing Creativity: A Comparison of Humans and LLMs
Source: J Intell. 2025 Jul 3;13(7):80. doi: 10.3390/jintelligence13070080 (PMC12295035; doi:10.3390/jintelligence13070080)
Supplement: Supplementary file 1 [file jintelligence-13-00080-s001.zip › Supplementary Folder/Stage 1 - Story Collection/Originally Collected Stories/Chinese Human Participants/Story 7 Non-Creative.pdf]

Chinese original version

作为中国超一线城市之一的广州市一直以来都以拥挤出名，特别是上下班时期的地铁里。行色匆匆的人们仿佛一个个生活失去了色彩的黑白照片，日复一日重复着相同的行迹。

阿佳大学毕业后选择留在这个春天时墙壁也会哭泣的城市，即使她时不时也会想念北方分明的四季和妈妈蒸的热腾腾的包子。阿佳在家乡读高中时被霸凌的回忆仿佛路边老槐树错综复杂的根系，紧紧地缠绕住她的灵魂，使她无法迈出踏上返乡之旅的脚步。

他人时常说，食在广东，可是无论怎样的美食都无法超越妈妈的厨艺。又结束了一天无意义般重复着的工作，加完班的阿佳走出灯光昏黄的老旧写字楼，肚子已经饿到麻木。为了这三千块钱一个月的工资真的值得吗？这点钱在超一线城市实在是无法支撑过多的花销，所以阿佳入职以来三个月了一直都吃公司食堂的菜或者泡面。“可是今天是母亲节啊。。。好想念妈妈和她包的皮薄馅儿多的三鲜粉丝包子啊”阿佳心想。

阿佳拖着疲惫的步伐走在仍旧热闹的老城区街头，不想这么快就回到公司那闷热的让人喘不过气的六人间宿舍里。夜晚的老城区充满着烟火气，神游的阿佳被一股莫名熟悉的香味勾回了神。只见街边一个小商贩的推车上摆了三笼热腾腾的包子，而且竟然一块钱一个！一口气买下十个包子的阿佳咬上包子的第一口就知道这一定是老乡才能做出的家乡的手艺。虽然便宜但是内馅儿充斥着海的鲜味，实在是太美味了。

吃饱喝足的阿佳感觉自己的灵魂受到了安抚，连明天要早八上班都仿佛不那么痛苦了。

English translation

As one of China's top-tier cities, Guangzhou has always been known for its congestion, especially during rush hours on the subway. People in a hurry seem like black and white photos of life that has lost its color, repeating the same paths day after day.

After graduating from university, A Jia chose to stay in this city where even the walls "cry" in spring, even though she occasionally misses the distinct four seasons of the north and the steaming buns her mother used to steam. The memories of being bullied in high school in her

hometown are like the intricate root system of an old locust tree on the side of the road, tightly entwined around her soul, preventing her from taking the step to return home.

People often say, "Eat in Guangdong," but no matter how delicious the food is, it cannot surpass her mother's cooking. After another meaningless day of repetitive work and overtime, A Jia walked out of the old office building with dim lights, her stomach numb from hunger. Is it really worth it for a monthly salary of three thousand yuan? This amount of money really cannot support too many expenses in a top-tier city, so A Jia has been eating company canteen food or instant noodles since she started working three months ago. "But today is Mother's Day... I really miss my mother and the thin-skinned, rich-filled Sanxian vermicelli buns she makes," A Jia thought.

A Jia dragged her tired steps along the still bustling streets of the old town, not wanting to return so quickly to the stuffy, breathless six-person dormitory of the company. The night in the old town was full of the smell of smoke and fire, and A Jia, lost in thought, was drawn back by a strangely familiar fragrance. There was a small vendor's cart on the street with three steaming baskets of buns, and surprisingly, they were only one yuan each! A Jia, who bought ten buns at once, knew with the first bite of the bun that it must be the hometown craftsmanship that only a fellow villager could make. Although cheap, the filling is full of the freshness of the sea, which is too delicious.

After eating and drinking her fill, A Jia felt her soul was soothed, and even the fact that she had to go to work early at eight tomorrow morning seemed less painful.
