# Supplementary material for: The Effects of (Dis)similarities Between the Creator and the Assessor on Assessing Creativity: A Comparison of Humans and LLMs
Source: J Intell. 2025 Jul 3;13(7):80. doi: 10.3390/jintelligence13070080 (PMC12295035; doi:10.3390/jintelligence13070080)
Supplement: Supplementary file 1 [file jintelligence-13-00080-s001.zip › Supplementary Folder/Stage 1 - Story Collection/Originally Collected Stories/Chinese Human Participants/Story 8 Non-Creative.pdf]

Chinese original version

在一座繁华的内陆城市里，街道上总是熙熙攘攘，有一个小巷子，它远离了主街道的喧嚣，安静而温馨。

故事的主人公是一个年轻人，他在这里经营着一家顺德菜餐厅。餐厅的门面并不显眼，但每当饭点时刻，这里总是人声鼎沸。每一道菜都透露出年轻人对食材的深刻理解和对传统烹饪技艺的精湛掌握。他做的顺德的鱼生，以其独特的切割技艺和鲜美口感，让人回味无穷。而双皮奶的嫩滑与伦教糕的软糯，更是让人赞不绝口。

一天中午，餐馆里来了一位特别的客人，他是一位海洋生物学家。他刚从海边的研究站回来，风尘仆仆，但他的眼神里闪烁着对海洋的热爱和对工作的执着。海洋生物学家给年轻人讲述了海洋的奥秘和美丽，年轻人升起了对亲眼看一看美丽海洋的渴望。

为了实现自己去海边旅行的计划，年轻人格外用心的经营自己的餐厅，终于实现了梦想，踏上了旅途。站在海边，眺望着眼前的海洋。海风轻拂，海浪拍打着岸边，年轻人在海边漫步，感受着大自然的宁静与壮阔。海天一色，阳光洒在波光粼粼的海面上，像是撒下了一把把金色的珍珠。海鸥在空中翱翔，自由自在，让人心生羡慕。闭上眼睛，深呼吸，年轻人感受着海的气息，心中的烦恼和压力仿佛都被海浪带走了。此刻，他只想静静地享受这份宁静，让心灵得到净化和放松。海边的美景让人心旷神怡，仿佛与世隔绝，只愿时间在这一刻停留。

English translation

In a bustling inland city, the streets are always bustling with activity. There is a small alley that is away from the hustle and bustle of the main streets, quiet and warm.

The protagonist of the story is a young man who runs a Shunde cuisine restaurant here. The facade of the restaurant is not eye-catching, but every meal time, it is always full of noise. Each dish reveals the young man's deep understanding of the ingredients and his superb mastery of traditional cooking skills. His Shunde raw fish, with its unique cutting skills and delicious taste, is unforgettable. And the tenderness of the double skin milk and the soft glutinous rice cake are even more praiseworthy.

One noon, a special guest came to the restaurant. He is a marine biologist. He just came back from the research station by the sea, and he is covered with dust, but his eyes are shining with love for the sea and persistence for work. The marine biologist told the young man about the

mysteries and beauty of the ocean, and the young man's desire to see the beautiful ocean with his own eyes.

In order to realize his plan to travel to the seaside, the young man managed his restaurant with extra care and finally realized his dream and set out on the journey. Standing by the sea and looking at the ocean in front of him. The sea breeze is blowing, and the waves are hitting the shore. The young man is walking on the beach, feeling the tranquility and magnificence of nature. The sea and the sky are the same color, and the sunlight is sprinkled on the sparkling sea, like scattering a handful of golden pearls. Seagulls are soaring in the sky, free and easy, making people envious. Close your eyes, take a deep breath, and the young man feels the breath of the sea, and the troubles and pressures in his heart seem to be taken away by the waves. At this moment, he just wants to quietly enjoy this tranquility and let his soul be purified and relaxed. The beautiful scenery by the sea is refreshing, as if it is isolated from the world, and only wishes that time would stop at this moment.
