# Supplementary material for: The Effects of (Dis)similarities Between the Creator and the Assessor on Assessing Creativity: A Comparison of Humans and LLMs
Source: J Intell. 2025 Jul 3;13(7):80. doi: 10.3390/jintelligence13070080 (PMC12295035; doi:10.3390/jintelligence13070080)
Supplement: Supplementary file 1 [file jintelligence-13-00080-s001.zip › Supplementary Folder/Stage 1 - Story Collection/Originally Collected Stories/Chinese Human Participants/Story 9 Non-Creative.pdf]

Chinese original version

在一个平凡的清晨，第一缕阳光照进紫蔷的房间，闹钟也恰好叮铃铃地响起来，她闭着眼挥舞着手试图关停闹钟，试了几次没成功后就疲惫地睁开眼。像往常一样，刷牙漱口，然后出门去买早餐。街边的店铺大多已经开着门，路上已经有不少车辆，再过一会可能就要开始堵塞了。紫蔷熟练地在早餐店点了一个肠粉，电视正在回放昨日的新闻。新闻主持说，本地S市最大的食品企业顾氏集团正在举办一场厨王争霸赛，奖金高达100万元。

紫蔷听到这条新闻，突然眉头一皱，这或许是个接近顾氏的好机会。她根据比赛指引，报了名，初赛时间是周五，她还有三天的准备时间。紫蔷虽然没有专业学过厨艺，但是从小对烹饪很有兴趣，所以有空也会钻研。经过三天的临时抱佛脚，不知不觉就到了初赛，比赛题目是辣非辣。大家看到这个题目都一头雾水，紫蔷脑袋灵光一现，突然有了主意，她把辣椒里面的籽全部抠出，然后过水三遍后放在一边；把白巧克力融化后，用竹签点一点，放在食用纸上冷却固化，固化后的白巧克力点就像辣椒里面的籽；然后小心翼翼地放进辣椒。当评委看到紫蔷碟子上的辣椒，都面露不屑仿佛接下去就要淘汰她，吃进第一口的时候，他们惊讶了，质感是辣椒，但是味道却是仿佛初恋的甜蜜，不想到不起眼的她呈现出如此特别的甜品。

紫蔷顺利晋级，然后得到机会去参观顾氏公司。在会议室里，她看到坐在正中央掌管整个集团顾氏最年轻的继承人顾得淼。她目不转睛看着顾得淼的时候，顾得淼也注意到她，她赶忙把视线转走。会议结束后，她离开公司，准备回家。经过海滩的时候，她突然听到有人喊救命。紫蔷赶忙下车，顺着声音游向海里。经过九牛二虎之力，她把人救出，这个人正是顾德淼的弟弟顾得旺。原来他游着游着突然腿抽筋使不上去，为了感谢她，他邀请紫蔷去家里吃饭。晚上，紫蔷再次见到顾得淼，两人视线交汇，彷佛早就认识。

English translation

On an ordinary morning, the first rays of sunlight streamed into Zi Qiang's room, and the alarm clock also rang just in time, "ding ding ding". She closed her eyes and waved her hand, trying to turn off the alarm clock. After several unsuccessful attempts, she opened her eyes wearily. As usual, she brushed her teeth and rinsed her mouth, then went out to buy breakfast. Most of

the shops on the street were already open, and there were already quite a few vehicles on the road, which might start to get congested in a while. Zi Qiang skillfully ordered a rice noodle roll at the breakfast shop, and the TV was replaying yesterday's news. The news anchor said that the largest food company in the local city S, the Gu Group, is holding a cooking competition with a prize of up to 1 million yuan.

Upon hearing this news, Zi Qiang suddenly frowned, thinking this might be a good opportunity to get close to the Gu Group. She followed the competition instructions and registered, with the preliminary competition scheduled for Friday, leaving her three days to prepare. Although Zi Qiang had not professionally studied cooking, she had always been interested in it since childhood and would study it in her spare time. After three days of last-minute preparation, the preliminary competition arrived. The competition topic was "Spicy but not spicy." Everyone was puzzled by this topic, but Zi Qiang had a sudden inspiration. She removed all the seeds from the chili peppers and rinsed them three times, setting them aside. She melted the white chocolate and, with a bamboo skewer, dotted a little on edible paper to cool and solidify. The solidified white chocolate dots were like the seeds inside the chili peppers. Then she carefully placed them inside the chili peppers. When the judges saw the chili peppers on Zi Qiang's plate, they looked disdainful as if they were about to eliminate her, but they were surprised when they took the first bite. The texture was that of chili peppers, but the taste was as sweet as first love, and they did not expect such an unremarkable person to present such a unique dessert.

Zi Qiang advanced smoothly and then had the opportunity to visit the Gu Group. In the meeting room, she saw Gu Demiao, the youngest heir of the Gu Group, sitting in the center and controlling the entire group. When she stared at Gu Demiao, he also noticed her, and she quickly averted her gaze. After the meeting ended, she left the company and prepared to go home. When passing by the beach, she suddenly heard someone shouting for help. Zi Qiang quickly got out of the car and swam towards the sea following the sound. After great effort, she rescued the person, who turned out to be Gu Demiao's brother, Gu Dewang. It turned out that he had a leg cramp while swimming and couldn't move. To thank her, he invited Zi Qiang to have dinner at home. In the evening, Zi Qiang saw Gu Demiao again, and their eyes met as if they had known each other for a long time.
