# Supplementary material for: The Effects of (Dis)similarities Between the Creator and the Assessor on Assessing Creativity: A Comparison of Humans and LLMs
Source: J Intell. 2025 Jul 3;13(7):80. doi: 10.3390/jintelligence13070080 (PMC12295035; doi:10.3390/jintelligence13070080)
Supplement: Supplementary file 1 [file jintelligence-13-00080-s001.zip › Supplementary Folder/Stage 1 - Story Collection/Originally Collected Stories/Western AI - ChatGPT/Story 1 - Creative.pdf]

English original version

On the bustling streets of New York City, where the ceaseless symphony of honking cabs, chattering pedestrians, and distant sirens played a familiar tune, lived a peculiar character named Elara. She wasn’t just another face in the crowd; Elara had a penchant for discovering the extraordinary hidden within the ordinary.

One rainy afternoon, as the city roared with the urgency of a thousand hurried footsteps, Elara found herself in an unusual predicament. Her umbrella had given up against the onslaught of the rain, leaving her drenched and cold. Seeking refuge, she dashed into a small, quaint bookstore nestled between a towering office building and a bustling café. The bookstore, aptly named "Hidden Realms," was a labyrinth of towering shelves, each crammed with stories waiting to be told.

Elara's eyes scanned the room, eventually settling on an old, dusty tome with a golden cover that shimmered despite the dim lighting. The title read, "The Alchemist’s Map." Intrigued, she flipped it open and discovered it wasn't just a book, but a gateway to an adventure. Inside was a map that promised to lead its bearer to the mythical "Feast of Realms," a legendary gathering rumored to serve the most delectable and magical dishes in existence.

Driven by curiosity and a growling stomach, Elara decided to follow the map’s cryptic directions. Her journey led her through the winding alleys of the city, each turn taking her further from the familiar cacophony of the streets and deeper into a world only a few had seen.

Halfway through her journey, as twilight painted the sky in hues of orange and pink, Elara stumbled upon a hidden courtyard. There, she encountered a rustic inn with a sign that read, "The Hearthstone Inn - Where Legends Dine." The innkeeper, an elderly man with twinkling eyes and a knowing smile, welcomed her in. The inn's interior was warm and inviting, with the scent of roasted meats and freshly baked bread wafting through the air.

Elara was seated at a grand oak table, and soon, a feast unlike any other was laid out before her. There was a savory stew that seemed to change flavors with each bite, golden loaves of bread that never seemed to diminish, and a crystal goblet filled with a drink that sparkled like the night sky. Every mouthful was a revelation, a symphony of tastes that danced on her tongue and left her yearning for more. This was the legendary "Feast of Realms" she had read about, a meal that was said to be unforgettable and yet indescribable.

Rejuvenated and deeply satisfied, Elara continued her journey, the map now leading her out of the city and towards the coast. The air grew saltier, and the distant sound of waves crashing against the shore grew louder. As she reached the end of her path, she stood before a vast, serene sea, its waters glistening under the moonlight. The map had led her to a secluded beach, where the sands sparkled as if kissed by a thousand stars.

Elara walked towards the water, feeling the cool waves lap at her feet. In her hand, the map transformed into a small, golden shell, glowing softly. She realized that her adventure wasn't just about the destination, but the journey and the experiences she had along the way. Standing there, with the vast expanse of the sea before her, Elara felt a sense of peace and fulfillment. She had discovered a hidden realm not just in the world, but within herself.

As the night embraced the world, Elara sat by the shore, watching the moon’s reflection dance on the waves, and she knew that her story was just beginning.

Chinese translation

在纽约市熙熙攘攘的街头，汽车喇叭声、行人喧哗声与远处警笛声交织成一曲熟悉的交响乐。在这座永不停歇的城市中，住着一位与众不同的人物，名叫伊拉拉。她并不是人群中普普通通的面孔——伊拉拉总能在平凡中发现不平凡的存在。

一个雨后的午后，城市在成千上万匆忙脚步的催促下喧嚣不已，伊拉拉却陷入了一场意外的窘境。她的伞在滂沱大雨的攻击下败下阵来，使她全身湿透、寒意袭人。为了寻找避雨之地，她冲进了一家藏身于高楼与咖啡馆之间的小而雅致的书店。这家名为“隐秘境界”的书店，宛如迷宫般堆满了高高的书架，每一层都塞满了等待被诉说的故事。

伊拉拉的目光在屋内四处扫视，最终停留在一本旧旧的、封面闪耀着金光的书籍上。尽管灯光昏暗，封面仍熠熠生辉。书名是《炼金术士的地图》。她好奇地翻开，发现这不仅仅是一本书，而是一扇通往冒险的大门。书中藏着一张地图，据说可以引领持有者前往传说中的“诸界盛宴”——一个据说汇集世间最美味、最具魔力料理的传奇盛会。

在好奇心与饥肠辘辘的驱使下，伊拉拉决定照着地图上神秘的指引前进。她穿梭于城市的曲折小巷，逐渐远离街头熟悉的喧嚣，走入只有极少数人曾涉足的奇异世界。

旅途过半时，暮色将天空染成橙粉交错的色调，伊拉拉无意中发现了一个隐藏的庭院。那里坐落着一家古朴的客栈，门前的招牌写着：“炉石客栈——传奇聚餐之所。”店主是一位眼神闪烁、笑容神秘的老人，热情地迎她入内。客栈内部温暖而舒适，空气中弥漫着烤肉和新鲜面包的香气。

伊拉拉坐在一张巨大的橡木餐桌前，随即一场前所未有的盛宴呈现在她眼前。有一道咸香浓郁的炖菜，每一口的味道都略有不同；有闪着金光的面包，仿佛永远吃不完；还有一只水晶酒杯，杯中饮品如星空般闪烁。每一口都是一种启示，是味觉上的交响乐，在她舌尖跳跃，令人回味无穷。这正是她在书中读到的“诸界盛宴”，传说中令人永生难忘、却难以言喻的盛餐。

在美食与温暖中恢复元气、满足至极的伊拉拉继续踏上旅程。此时地图将她引出城市，朝着海岸的方向前行。空气变得更加咸湿，远方传来海浪拍打岸边的声音。终于，她抵达了旅途的终点——一片广阔宁静的海滩，沙粒在月光下闪烁，仿佛被千万颗星星亲吻过。

伊拉拉走向海边，感受冰凉的海水拍打着双脚。此时，她手中的地图渐渐化为一枚微光流转的金色贝壳。她顿悟，这趟旅程的意义不仅仅在于抵达终点，而在于沿途的经历与成长。站在浩瀚大海前，伊拉拉心中涌起一股宁静与圆满的感受。她发现了一个隐秘的境界，不只是藏在世界某处，更藏在她自身深处。

夜幕缓缓降临，伊拉拉坐在海边，凝望着月光在水面上的舞动反射，心中明白——她的故事，才刚刚开始。
