# Supplementary material for: The Effects of (Dis)similarities Between the Creator and the Assessor on Assessing Creativity: A Comparison of Humans and LLMs
Source: J Intell. 2025 Jul 3;13(7):80. doi: 10.3390/jintelligence13070080 (PMC12295035; doi:10.3390/jintelligence13070080)
Supplement: Supplementary file 1 [file jintelligence-13-00080-s001.zip › Supplementary Folder/Stage 1 - Story Collection/Originally Collected Stories/Western AI - ChatGPT/Story 10 - Non-creative.pdf]

English original version

On a bustling Friday afternoon, the streets of New York City were teeming with life. Cars honked impatiently, and pedestrians hurriedly crossed avenues, each person wrapped in their own thoughts and destinations. Among the crowd was Sarah, a university student, weaving her way through the masses with practiced ease. She had just finished her last class for the week and was eagerly anticipating a relaxing weekend.

After navigating through the crowded streets, Sarah finally arrived at her favorite café, a cozy little place nestled between two towering skyscrapers. She ordered her usual caramel macchiato and found a seat by the window, where she could watch the city's constant motion. She pulled out her laptop and began to type, hoping to get a head start on her assignments.

Hours flew by, and Sarah's stomach began to rumble. She realized it was well past lunchtime and decided to treat herself to a proper meal. She remembered a quaint Italian restaurant a few blocks away that she had been meaning to try. With a final sip of her coffee, she packed up and headed out.

The restaurant, Bella Napoli, was a charming spot with red-checkered tablecloths and the mouth-watering aroma of freshly baked bread and herbs. Sarah ordered the lasagna, a house specialty, and when it arrived, she knew she had made the right choice. The layers of pasta, cheese, and rich tomato sauce melted in her mouth, each bite better than the last. She savored the meal, feeling content and satisfied.

With her hunger appeased and a smile on her face, Sarah decided to take a walk along the waterfront. The city’s riverwalk was a favorite spot of hers, offering a brief escape from the urban chaos. As she strolled along the path, the sound of the water lapping gently against the shore was soothing, and she felt the week's stress begin to ebb away.

Sarah found a bench facing the sea, where she sat down and let her mind wander. The view was stunning; the sun was beginning to set, casting a golden hue over the water. Boats sailed lazily by, and seagulls called out to one another as they flew overhead. It was moments like these that made her appreciate the city's unique blend of energy and tranquility.

As the evening grew cooler, Sarah felt a sense of calm wash over her. She took a deep breath of the salty sea air and knew that, despite the busy streets and the constant rush, there was always a peaceful place to be found in the city. She stayed a while longer, watching the sun dip below the horizon, before finally heading back home, ready to embrace whatever the weekend had in store.

Chinese translation

在一个繁忙的星期五下午，纽约市的街道上充满了生机。汽车喇叭声不绝于耳，行人匆匆穿越街道，每个人都沉浸在自己的思绪和目的地之中。在人群中，有一位名叫莎拉的大学生，她熟练地在人群中穿行。她刚刚结束了一周的最后一节课，期待着一个轻松的周末。
经过拥挤的街道，莎拉终于来到了她最喜欢的咖啡馆，一个藏在两座高楼之间的温馨小地方。她点了一杯常喝的焦糖玛奇朵，找了个靠窗的座位，观看着城市的熙熙攘攘。她拿出笔记本电脑，开始打字，希望能提前完成一些作业。

时间飞逝，莎拉的肚子开始咕咕叫。她意识到已经过了午餐时间，决定犒劳自己一顿美餐。她记得几条街外有一家她一直想试试的意大利餐馆。最后喝了一口咖啡，她收拾好东西出发了。

那家餐馆，叫做贝拉那不勒斯，是一个带有红色格子桌布的迷人地方，散发着新鲜烘焙面包和香草的诱人香气。莎拉点了千层面，这道招牌菜果然没有让她失望。面条、奶酪和浓郁的番茄酱层层叠叠，入口即化，每一口都比前一口更美味。她享受着这顿饭，感到满足和开心。

饱餐一顿后，莎拉决定沿着海滨散步。城市的河滨步道是她最喜欢的地方之一，可以短暂逃离城市的喧嚣。当她沿着小路漫步时，水轻轻拍打岸边的声音令人心旷神怡，她感到一周的压力开始消散。

莎拉找到一个面朝大海的长椅，坐下来让思绪自由飘荡。景色非常迷人，夕阳开始落下，为水面投射出金色的光芒。船只悠闲地驶过，海鸥在头顶盘旋，相互鸣叫。在这样的时刻，她感到特别欣慰，即使城市如此忙碌，总有一个宁静的地方可以找到。

随着夜晚变得凉爽，莎拉感到一阵平静的感觉。她深深吸了一口咸咸的海风，知道尽管街道繁忙、匆忙不断，城市中总有一个宁静的地方可以找到。她在那儿多坐了一会儿，看着太阳沉入地平线，最后才回家，准备好迎接周末的一切。
