# Supplementary material for: The Effects of (Dis)similarities Between the Creator and the Assessor on Assessing Creativity: A Comparison of Humans and LLMs
Source: J Intell. 2025 Jul 3;13(7):80. doi: 10.3390/jintelligence13070080 (PMC12295035; doi:10.3390/jintelligence13070080)
Supplement: Supplementary file 1 [file jintelligence-13-00080-s001.zip › Supplementary Folder/Stage 1 - Story Collection/Originally Collected Stories/Western AI - ChatGPT/Story 2 - Creative.pdf]

English original version

In the heart of New York City, where skyscrapers kissed the sky and taxis honked in a never-ending symphony, there lived a young woman named Eleanor. She navigated the bustling streets with the grace of someone who had long since learned to dance to the chaotic rhythm of the city. Every morning, she would walk past the corner of 5th Avenue and 42nd Street, where a street musician played his saxophone, the melody weaving through the cacophony of traffic and hurried footsteps.

Eleanor worked as a graphic designer for a prestigious advertising firm. Her days were filled with colors and shapes, creativity pouring from her fingertips as she crafted visuals that would capture the attention of passersby. Despite the demands of her job, she never lost her love for the small wonders of the city. She found joy in the little things: the aroma of freshly brewed coffee from the cart on the corner, the vibrant murals that adorned brick walls, and the diverse tapestry of people who called New York home.

One afternoon, after a particularly grueling meeting, Eleanor decided to treat herself to lunch at her favorite hidden gem—a tiny bistro tucked away in a narrow alley. The restaurant was known for its delectable fusion cuisine, blending flavors from around the world into unforgettable dishes. She ordered a plate of truffle-infused gnocchi, the smell alone promising a culinary adventure. The first bite was heavenly, the flavors dancing on her tongue, each ingredient playing its part in a perfect harmony of taste. She savored every mouthful, the meal a delightful respite from the intensity of her workday.

As she left the bistro, her phone buzzed with a message from her best friend, Tom. He had just returned from a year-long expedition to Antarctica and wanted to catch up over the weekend. They decided to meet at Coney Island, a place that held countless memories of their childhood. Eleanor felt a wave of nostalgia as she thought about their countless summers spent on the boardwalk, laughing and playing games, the scent of salty air and cotton candy mingling in the breeze.

That Saturday, Eleanor and Tom strolled along the beach, the Atlantic Ocean stretching out before them, its vastness a reminder of how small their worries really were. They reminisced about their adventures, the sun dipping below the horizon, painting the sky in hues of orange and pink. As they sat on the sand, listening to the gentle lapping of the waves, Eleanor realized that amidst the chaos of the city, it was these moments of connection and tranquility that truly mattered.

And so, under the watchful gaze of the stars, Eleanor and Tom made a pact to cherish the simple pleasures, to find beauty in the ordinary, and to always make time for the sea, where the endless possibilities of life met the boundless horizon.

Chinese translation

在纽约市的中心，摩天大楼触及天空，出租车喇叭声不绝于耳，这里住着一个名叫艾莉诺的年轻女子。她像一个学会了与城市混乱节奏共舞的人一样，优雅地穿行于繁忙的街道。每天早晨，她都会经过第五大道和第四十二街的拐角处，那里有一位街头音乐家演奏萨克斯风，旋律在交通和匆忙的脚步声中穿梭。

艾莉诺是一家著名广告公司的平面设计师。她的日子充满了色彩和形状，创意从她的指尖倾泻而出，打造出吸引路人目光的视觉效果。尽管工作繁忙，她从未失去对城市小奇迹的热爱。她在小事中找到了快乐：街角咖啡车新煮咖啡的香气、装饰砖墙的生动壁画以及称纽约为家的多元人群。

一天中午，在一次特别艰难的会议后，艾莉诺决定去她最喜欢的隐秘餐馆享受午餐——这是一家隐藏在狭窄巷子里的小酒馆。这家餐厅以其令人难忘的美食融合菜肴而闻名，将世界各地的风味融为一体。她点了一盘松露意大利饺子，仅凭气味就能承诺一次美食冒险。第一口简直是天堂，味道在她的舌尖上跳跃，每一种成分在完美的味觉和谐中扮演着自己的角色。她细细品味每一口，这顿饭成为了她紧张工作日中的美好休憩。

离开小酒馆时，她的手机响起了最好的朋友汤姆的消息。他刚从为期一年的南极探险归来，想在周末叙旧。他们决定在科尼岛见面，这个地方承载着他们童年的无数回忆。艾莉诺回想着他们在木板路上度过的无数夏天，欢笑和游戏的时光，咸咸的空气和棉花糖的香气在微风中交织。

那个星期六，艾莉诺和汤姆漫步在海滩上，大西洋在他们面前延展开来，其广阔提醒着他们的烦恼是多么微不足道。他们回忆着过去的冒险，太阳渐渐沉入地平线，将天空染成橙色和粉色。当他们坐在沙滩上，聆听海浪轻拍的声音时，艾莉诺意识到，在城市的混乱中，真正重要的是这些连接和宁静的时刻。

于是，在星星的注视下，艾莉诺和汤姆约定珍惜简单的乐趣，在平凡中发现美，并永远为大海留出时间，因为生命的无限可能在那无边的地平线上相遇。
