# Supplementary material for: The Effects of (Dis)similarities Between the Creator and the Assessor on Assessing Creativity: A Comparison of Humans and LLMs
Source: J Intell. 2025 Jul 3;13(7):80. doi: 10.3390/jintelligence13070080 (PMC12295035; doi:10.3390/jintelligence13070080)
Supplement: Supplementary file 1 [file jintelligence-13-00080-s001.zip › Supplementary Folder/Stage 1 - Story Collection/Originally Collected Stories/Western AI - ChatGPT/Story 3 - Creative.pdf]

English original version

In the heart of a bustling metropolis, where skyscrapers kissed the clouds and neon signs painted the night sky with a kaleidoscope of colors, there stood a busy street. This street, known as Delmar Avenue, was a cacophony of sounds and sights. Pedestrians hurried along the sidewalks, their footsteps merging with the symphony of honking cars and rumbling buses. Vendors hawked their wares from makeshift stalls, enticing passersby with promises of exotic treasures and bargain prices.

Amidst the chaos of Delmar Avenue, there was a quaint little restaurant tucked away in a corner, its windows fogged with the warmth of laughter and conversation. This was "The Spice Emporium," renowned for its delectable cuisine that tantalized the taste buds and warmed the soul. Inside, the aroma of spices hung heavy in the air, mingling with the sizzle of pans and the bubbling of pots.

At a corner table sat Mia, a weary traveler seeking refuge from the frenetic energy of the city. She sipped on a steaming cup of chai, its spicy warmth seeping into her bones and chasing away the chill of the autumn evening. Across from her, a plate of fragrant biryani awaited, its colorful array of spices beckoning her to indulge.

As Mia savored each mouthful of the aromatic rice dish, she couldn't help but lose herself in its rich flavors and complex textures. It was as if each grain carried a story of its own, whispering tales of distant lands and ancient traditions.

With her hunger sated and her spirits lifted, Mia emerged from the sanctuary of "The Spice Emporium" and made her way towards the waterfront. The sea stretched out before her, its vast expanse shimmering in the moonlight. The gentle lapping of waves against the shore was a soothing melody, a stark contrast to the hustle and bustle of the city she had left behind.

As she stood on the edge of the pier, gazing out into the endless horizon, Mia felt a sense of peace wash over her. The sea had a way of putting things into perspective, reminding her of the vastness of the world and her place within it. With a contented sigh, she closed her eyes and let the rhythmic sound of the waves carry her away, lost in the timeless embrace of the ocean.

Chinese translation

在繁华的大都市中心，摩天大楼拥抱着云彩，霓虹灯用五彩斑斓的色彩点缀着夜空，有一条繁忙的街道。这条街道名为德尔马尔大街，是声音和景象的交织。行人匆匆忙忙地走在人行道上，他们的脚步声与汽车的喇叭声和巴士的轰鸣声融为一体。摊贩们在临时摊位上兜售着商品，用异国情调的宝藏和物美价廉的承诺诱惑着路人。

在德尔马尔大街的混乱中，有一家藏在角落里的古雅小餐馆，它的窗户被笑声和交谈的温暖弄得雾蒙蒙的。这就是“香料宝库”，以其令人垂涎欲滴的美食而闻名，这些美食挑逗着味蕾，温暖了灵魂。里面，香料的香气在空气中弥漫着，与锅碗瓢盆的烧烤声和沸腾声交织在一起。

在一张角落桌子上坐着米娅，一位疲惫的旅行者，她寻求着摆脱城市的狂热能量的避难所。她啜饮着一杯冒着热气的印度奶茶，辛辣的温度渗透进她的骨头，驱散了秋日夜晚的寒意。在她对面，摆着一盘香气扑鼻的印度香料炖饭，五颜六色的香料诱惑着她放纵自己。

当米娅品尝着每一口香气扑鼻的米饭时，她不禁沉浸在其浓郁的风味和复杂的口感中。仿佛每一粒米都承载着自己的故事，低声诉说着遥远的土地和古老的传统。

当她的饥饿得到满足，精神也得到振奋时，米娅走出了“香料宝库”的庇护所，朝着海滨走去。大海在她面前展开，其广阔的辽阔在月光下闪烁。海浪轻轻拍打着岸边，是一曲悠扬的旋律，与她刚刚离开的城市的喧嚣和繁忙形成了鲜明的对比。

当她站在码头的边缘，凝视着无尽的地平线时，米娅感到一股平静感袭上心头。大海有一种让事物变得清晰的方式，提醒着她世界的广阔以及自己在其中的位置。她满意地叹了口气，闭上了眼睛，让海浪的节奏将她带走，在海洋的永恒拥抱中迷失了自我。
