# Supplementary material for: The Effects of (Dis)similarities Between the Creator and the Assessor on Assessing Creativity: A Comparison of Humans and LLMs
Source: J Intell. 2025 Jul 3;13(7):80. doi: 10.3390/jintelligence13070080 (PMC12295035; doi:10.3390/jintelligence13070080)
Supplement: Supplementary file 1 [file jintelligence-13-00080-s001.zip › Supplementary Folder/Stage 1 - Story Collection/Originally Collected Stories/Western AI - ChatGPT/Story 4 - Creative.pdf]

English original version

The bustling street of New York City was a symphony of honking horns, distant sirens, and the chatter of a thousand conversations. Among the throngs of people, Amy stood at the corner of Fifth Avenue, her eyes scanning the sea of faces, searching for someone. She checked her watch for the umpteenth time, feeling a knot of anxiety tighten in her stomach. She was supposed to meet her brother, Jack, but he was nowhere to be seen.

As she waited, her mind drifted back to the last conversation they'd had a week ago. Jack had called her, his voice brimming with excitement about a mysterious project he was working on. He had always been the adventurous one, diving headfirst into new ventures without a second thought. Amy, the more cautious sibling, couldn't help but worry.

Finally, she spotted him weaving through the crowd, a wide grin on his face. "Amy!" he called out, waving. She sighed in relief and waved back. Jack reached her, slightly out of breath but beaming. "Sorry I'm late. You won't believe what I've been up to."

They walked together, and Jack led her to a small, tucked-away bistro he had discovered. The tantalizing aroma of freshly baked bread and sizzling meats greeted them as they entered. They found a cozy table by the window, and Jack ordered their meal—a feast of roasted chicken, garlic mashed potatoes, and a side of buttery asparagus. As they ate, Jack recounted his latest adventure.

"I've been working on a project with a marine biologist friend," he explained between bites. "We're developing a new type of underwater drone that can map the ocean floor in unprecedented detail. It's going to revolutionize our understanding of marine life and ecosystems."

Amy listened, captivated by his enthusiasm. The meal was delicious, each bite a comforting reminder of home and family. But underneath her enjoyment, a flicker of concern remained. Jack's ventures were often unpredictable, and she worried about his safety.

After their meal, Jack suggested a walk. They strolled through Central Park, the evening sun casting long shadows across the grass. Jack continued to talk about his project, his eyes lighting up with passion. "We're planning an expedition to the Mariana Trench next month. It's the deepest part of the ocean, and we're hoping to discover new species down there."

Amy stopped in her tracks, her heart pounding. "The Mariana Trench? Jack, that's incredibly dangerous!"

Jack turned to her, his expression earnest. "I know, Amy. But think about what we could learn. The risks are worth it."

She shook her head, fear and admiration battling within her. "Just promise me you'll be careful."

He nodded, his gaze steady. "I promise."

A month later, Amy stood on the deck of a research vessel, the salty sea breeze ruffling her hair. She had decided to join Jack on his expedition, unable to shake her concern. As the ship sailed

towards the open ocean, she marveled at the vast, endless expanse of blue stretching out before them. It was both beautiful and intimidating.

Jack joined her at the railing, his excitement palpable. "Ready to dive into the unknown?"

Amy smiled, her worry tempered by his unwavering confidence. "Ready as I'll ever be."

Together, they embarked on a journey into the depths of the sea, driven by curiosity and the unbreakable bond of family. As they descended into the dark, mysterious waters of the Mariana Trench, Amy couldn't help but feel a sense of awe and anticipation. The sea, with all its secrets, awaited them.

Chinese translation

纽约市熙熙攘攘的街头仿佛一场交响乐，汽车喇叭声、远处的警笛声以及无数对话的嘈杂声交织其间。在人潮涌动的人群中，艾米站在第五大道的街角，目光在人海中搜寻，焦急地寻找某个人。她又一次看了看手表，焦虑的情绪在胸口越缠越紧。她本该和哥哥杰克见面，可他却迟迟未现身。

等待中，她的思绪飘回了一周前他们的最后一次通话。当时，杰克打电话来，声音中满是激动，兴奋地谈起一个神秘的项目。他一向是那个敢于冒险的人，总是毫不犹豫地投身于各种新奇的尝试。而艾米，这个较为谨慎的妹妹，总忍不住为他担心。

终于，她在人群中看见他穿梭而来，脸上挂着灿烂的笑容。“艾米！”他边挥手边喊。她松了口气，也挥手回应。杰克跑到她面前，略显气喘，但满脸喜悦。“对不起我迟到了，你绝对想不到我最近在做什么。”

他们一起走着，杰克带她来到一家他新发现的小而隐秘的餐馆。一进门，迎面而来的就是新鲜烘焙面包和滋滋作响的肉类香气。他们找了一个靠窗的温馨小桌，杰克点了菜——烤鸡、蒜香土豆泥和黄油芦笋，一顿丰盛的晚餐。不一会儿，杰克开始讲述他最近的冒险。

“我最近在和一个海洋生物学家朋友合作，”他边吃边说，“我们正在研发一种新型水下无人机，可以以前所未有的精度绘制海底地图。这将彻底改变我们对海洋生态系统的认知。”

艾米听得入神，被他满腔的热情感染。餐点美味，每一口都带来家的温暖与熟悉。但在这份美好背后，她的心中依然泛着一丝担忧。杰克的冒险总是充满变数，她始终无法完全放心。

饭后，杰克提议散步。他们漫步在中央公园中，夕阳将草地上的影子拉得很长。杰克继续兴奋地谈着他的项目，眼神里闪耀着光芒：“我们下个月打算前往马里亚纳海沟，那是海洋最深的地方。我们希望在那里发现新的物种。”

艾米停下脚步，心跳加快。“马里亚纳海沟？杰克，那太危险了！”

杰克转向她，神情认真：“我知道，艾米。但想一想我们能学到的东西。这些风险，是值得的。”

她摇头，内心在恐惧与钦佩之间挣扎。“你一定要答应我，小心一点。”

他点了点头，目光坚定。“我答应你。”

一个月后，艾米站在一艘研究船的甲板上，海风吹动她的发丝。她最终决定加入杰克的远征，放不下内心的担忧。望着无边无际、蔚蓝浩瀚的大海，她既感到震撼，又略带畏惧。

杰克走到她身旁，兴奋之情溢于言表。“准备好潜入未知世界了吗？”

艾米笑了，杰克那份坚定的自信驱散了她的一部分担忧。“我尽可能准备好了。”

他们一同踏上了这段深入海洋深处的旅程，被好奇心驱动，也因家人的深厚情感紧紧相连。当他们缓缓潜入马里亚纳海沟那幽深神秘的水域时，艾米心中充满敬畏与期待。这片大海，连同它无数未被揭开的秘密，正静静地等着他们去发现。
