# Supplementary material for: The Effects of (Dis)similarities Between the Creator and the Assessor on Assessing Creativity: A Comparison of Humans and LLMs
Source: J Intell. 2025 Jul 3;13(7):80. doi: 10.3390/jintelligence13070080 (PMC12295035; doi:10.3390/jintelligence13070080)
Supplement: Supplementary file 1 [file jintelligence-13-00080-s001.zip › Supplementary Folder/Stage 1 - Story Collection/Originally Collected Stories/Western AI - ChatGPT/Story 5 - Creative.pdf]

English original version

The city never slept, not on Eighth Avenue. The constant hum of honking cars, the rhythmic clacking of footsteps on concrete, and the occasional outburst of street performers' melodies created an unending symphony of urban life. Amidst this ceaseless cacophony, a young woman named Lila maneuvered through the throngs of people. She clutched her sketchbook tightly to her chest, her mind a whirlpool of colors and ideas, desperately seeking a quiet corner in this bustling metropolis.

Lila had moved to the city with dreams of becoming an artist, but inspiration seemed as elusive as silence in the heart of the urban jungle. She wandered aimlessly, her eyes catching snippets of potential in the mundane—a flower vendor's cart, a child chasing pigeons, a busker strumming a soulful tune on his guitar. Yet, none of these moments felt complete, none compelling enough to be immortalized in her art.

Exhausted, Lila found herself drawn to a small, unassuming café tucked away between towering skyscrapers. The sign above the door read "Elysian Bites." She entered, the aroma of freshly brewed coffee and baked bread enveloping her like a warm embrace. The hustle and bustle outside felt worlds away, replaced by the soothing murmur of quiet conversations and the clinking of cutlery.

She ordered a meal—pan-seared salmon with a side of roasted vegetables—and settled into a corner booth. As she waited, she flipped open her sketchbook, idly doodling the café's interior. The meal arrived, and the first bite was a revelation. The salmon was perfectly cooked, its rich, buttery flavor dancing on her palate, while the vegetables offered a delightful contrast with their crisp, earthy tones. For the first time in weeks, Lila felt a spark of contentment.

Inspired, she began sketching in earnest, the lines flowing effortlessly across the page. The café transformed into a vivid landscape under her deft hand, each patron a character in her unfolding story. She lost herself in the act of creation, her earlier frustration dissipating like mist under the morning sun.

Hours passed, and the once-crowded café began to thin out. Lila looked up from her work, her eyes alight with the joy of artistic fulfillment. She left a generous tip for the waiter, who had watched her with a knowing smile, and stepped back into the city's embrace, her mind buzzing with newfound clarity.

As the sun dipped below the horizon, Lila felt an irresistible urge to visit the place that had always brought her peace—the sea. She took a cab to the edge of the city, where the concrete gave way to sand, and the relentless noise was replaced by the soothing sound of waves crashing against the shore.

The beach was almost deserted, save for a few solitary figures walking along the water's edge. Lila found a quiet spot and sat down, the cool sand a welcome contrast to the day's hectic energy. She opened her sketchbook once more, capturing the serene beauty of the sea at twilight. The sky was a canvas of oranges and purples, the waves a symphony of blues and whites.

As she sketched, Lila felt a profound sense of connection to the world around her. The city's chaos and the sea's tranquility were two sides of the same coin, each essential to her journey

as an artist. She realized that inspiration could be found in the most unexpected places—a bustling street, a delicious meal, or the timeless dance of the ocean.

With the sound of the waves whispering in her ears, Lila knew she had found her muse. The city had given her its stories, and now, she was ready to tell them.

Chinese translation

城市从不沉睡，尤其是在第八大道上。汽车喇叭声持续不断，脚步声在水泥地上有节奏地敲响，偶尔夹杂着街头艺人旋律的爆发，一同奏出一曲不休不止的都市交响乐。在这片喧嚣之中，一位名叫莉拉的年轻女子在人群中穿梭前行。她紧紧抱着自己的素描本，脑海中翻涌着色彩与灵感的漩涡，拼命在这座喧闹的大都市中寻找一处安静的角落。

莉拉怀揣着成为艺术家的梦想来到这座城市，然而灵感却仿佛比城市中央的宁静更难觅得。她漫无目的地走着，目光不断捕捉着日常中的片段——鲜花摊前斑斓的色彩、追逐鸽子的小孩、街头艺人拨动吉他弦时流露出的情感。但这些画面始终不完整，不足以成画。

精疲力竭之际，莉拉被一间隐藏在摩天大楼之间的小咖啡馆吸引。门上的招牌写着“极乐一味（Elysian Bites）”。她走了进去，迎面而来的咖啡香与新鲜面包香气如同温柔的怀抱将她包围。外面的喧嚣仿佛已远在天边，取而代之的是细语低语与餐具轻响交织出的宁静氛围。

她点了一份香煎三文鱼配烤时蔬，找了个角落的卡座坐下。在等待餐点时，她随意地翻开素描本，开始涂画咖啡馆的内部。饭菜端上来的时候，第一口便令她震撼。三文鱼外焦里嫩，入口即化，黄油的浓香在味蕾上轻盈起舞；烤蔬菜则脆嫩可口，带来质朴的大地气息。这是她数周以来第一次感受到真正的满足。

灵感随之而来，莉拉开始专注地作画，笔尖飞舞，线条自如地铺展开来。咖啡馆在她的笔下悄然变幻成一个充满生命的画面，每位食客都成了她故事中的角色。她沉浸于创作中，之前那股压抑与烦躁仿佛晨雾般悄然散去。

不知过了多久，原本热闹的咖啡馆也逐渐安静下来。莉拉从创作中抬起头，眼中闪耀着创作带来的喜悦与满足。她给服务员留下一笔丰厚的小费，那位早已察觉她状态转变的服务生，只是回以会心一笑。随后，她重新踏入城市的怀抱，脑海中灵感泉涌，清明而坚定。

太阳慢慢落下地平线，莉拉忽然涌起一股强烈的渴望，想要去那个总能带给她平静的地方——海边。她叫了一辆出租车，驶向城市边缘。那里的钢筋水泥让位于细软沙滩，喧嚣的城市噪音也被海浪轻拍岸边的声音所取代。

海滩上几乎没有人，只有零星几个身影在水边散步。莉拉找到一处僻静之地坐下，冰凉的沙子为她带来一天忙碌后的慰藉。她再次打开素描本，描绘起这片暮色中的海景。天空仿佛一块渐变的画布，铺展着橙色与紫色的余晖；而海浪则像交响乐般翻涌着湛蓝与洁白。

在笔尖舞动之间，莉拉感受到了一种深刻的连接——与这个世界，也与自己。城市的混乱与大海的宁静，就像硬币的两面，缺一不可，共同构成了她艺术旅程的一部分。她终于明白，灵感往往藏在最意想不到的角落——喧闹的街头、一顿美味的餐点，或是海浪与晚霞交汇的瞬间。

在海浪轻声低语的陪伴下，莉拉知道，她终于找到了自己的缪斯。城市给予了她无数故事，而她，也准备好将它们一一讲述。
