# Supplementary material for: The Effects of (Dis)similarities Between the Creator and the Assessor on Assessing Creativity: A Comparison of Humans and LLMs
Source: J Intell. 2025 Jul 3;13(7):80. doi: 10.3390/jintelligence13070080 (PMC12295035; doi:10.3390/jintelligence13070080)
Supplement: Supplementary file 1 [file jintelligence-13-00080-s001.zip › Supplementary Folder/Stage 1 - Story Collection/Originally Collected Stories/Western AI - ChatGPT/Story 6 - Non-creative.pdf]

English original version

On a typical Monday morning, John found himself navigating through the bustling streets of New York City. The sidewalks were crowded with people rushing to work, the air filled with the sounds of honking cars and distant sirens. As he weaved through the throngs of pedestrians, John couldn't help but feel a mix of excitement and anxiety. It was the start of another hectic week at the university, and he had a lot on his plate.

John was a junior majoring in business administration, and his schedule was packed with classes, group projects, and part-time work at a local café. Today, however, he was particularly looking forward to his lunch break. He had recently discovered a small, family-owned restaurant near campus that served the most delicious homemade pasta. The thought of that tasty meal kept him motivated as he attended his morning lectures.

By noon, John's stomach was growling. He hurried over to the restaurant, eager to indulge in his favorite dish – spaghetti carbonara. As he entered the cozy eatery, the warm aroma of garlic and herbs instantly put him at ease. He greeted the owner, Mrs. Rossi, who welcomed him with a friendly smile and quickly brought out his order. The first bite was heavenly – the perfect blend of creamy sauce, crispy pancetta, and al dente pasta. It was moments like these that made the daily grind worthwhile.

Feeling satisfied and recharged, John returned to campus for his afternoon classes. The hours seemed to fly by as he immersed himself in discussions about marketing strategies and financial analysis. Before he knew it, the day was drawing to a close. His last class ended, and John decided to take a walk by the waterfront to unwind.

The sun was setting as he reached the Hudson River. The sky was painted in hues of orange and pink, reflecting off the calm waters. John found a quiet spot to sit and watched the boats gently bobbing in the harbor. The sea breeze was cool and refreshing, a stark contrast to the busy streets he had navigated earlier. He took a deep breath, savoring the tranquility and the distant sound of waves lapping against the shore.

As he sat there, John realized how much he appreciated these simple moments of peace amidst his chaotic city life. The sea had a way of soothing his mind, reminding him to take things one step at a time. With renewed clarity, he stood up, ready to face the rest of the week with a calm and focused spirit.

Chinese translation

在一个典型的星期一早晨，约翰发现自己在纽约市繁忙的街道上穿行。人行道上挤满了赶着上班的人，空气中充斥着汽车喇叭声和远处的警报声。当他在人群中穿梭时，约翰不禁感到既兴奋又焦虑。这是大学里另一个忙碌的一周的开始，他有很多事情要处理。

约翰是商学专业的三年级学生，他的日程表排满了课程、小组项目和在当地一家咖啡馆的兼职工作。然而，今天他特别期待午休时间。他最近发现了一家靠近校园的家庭餐馆，那里的自制意大利面非常美味。想到那美味的午餐，他在早上的讲座中保持了动力。

到了中午，约翰的肚子开始咕咕叫了。他匆匆赶到餐馆，急切地想享用他最喜欢的菜肴**——培根蛋面。当他走**进这家温馨的小餐馆时，大蒜和香草的温暖香气立刻让他放松下来。他向老板

罗西太太打招呼，她友好地微笑着迎接他，很快就端上了他的订单。第一口简直是天堂**——奶油**酱、脆培根和刚刚煮熟的意大利面的完美融合。正是这样的时刻，让日常的辛劳变得值得。

吃得心满意足后，约翰回到校园上下午的课。时间在讨论市场策略和财务分析时似乎飞快流逝。不知不觉中，天色已晚。他的最后一节课结束后，约翰决定去海滨散步放松一下。

当他到达哈德逊河时，太阳正在落山。天空被染成了橙色和粉色，倒映在平静的水面上。约翰找到一个安静的地方坐下，看着港口里轻轻摇晃的船只。海风凉爽清新，与他早些时候穿行的繁忙街道形成了鲜明对比。他深吸一口气，享受着宁静和远处拍打岸边的海浪声。

坐在那里，约翰意识到他是多么珍惜在这个喧嚣城市生活中的简单宁静时刻。大海有一种安抚他心灵的方式，提醒他一步一步地去应对生活中的挑战。重新获得了清晰的思路后，他站起来，准备以平静和专注的精神迎接接下来的这一周。
