# Supplementary material for: The Effects of (Dis)similarities Between the Creator and the Assessor on Assessing Creativity: A Comparison of Humans and LLMs
Source: J Intell. 2025 Jul 3;13(7):80. doi: 10.3390/jintelligence13070080 (PMC12295035; doi:10.3390/jintelligence13070080)
Supplement: Supplementary file 1 [file jintelligence-13-00080-s001.zip › Supplementary Folder/Stage 1 - Story Collection/Originally Collected Stories/Western AI - ChatGPT/Story 7 - Non-creative.pdf]

English original version

On a bright Saturday morning in New York City, the streets buzzed with the usual hustle and bustle. The air was filled with a symphony of car horns, chatter, and the rhythmic clatter of footsteps against the pavement. The busy street outside Amelia's apartment was no exception. She stood at her window, sipping her coffee, watching the vibrant city wake up. Her plans for the day included meeting her old college friends for brunch, a tradition they upheld every month despite their hectic schedules.

As she made her way through the crowded sidewalks, Amelia thought about how much she cherished these moments. The city, with its constant motion, was where she felt most alive. She navigated through the sea of people, the familiar sights and sounds bringing a sense of comfort.

Amelia arrived at the quaint café where her friends were waiting. The aroma of freshly brewed coffee and baked goods filled the air as she stepped inside. They greeted her with warm smiles and hugs. The café, nestled in a quieter corner of the city, was their little oasis away from the chaos outside.

They settled into their favorite booth and quickly fell into their usual rhythm of conversation, laughter, and catching up on each other's lives. When the waiter arrived, they eagerly placed their orders, excited for the meal they had been anticipating all week. Amelia opted for the café's famous avocado toast with poached eggs, while her friends chose an assortment of dishes ranging from fluffy pancakes to savory omelets.

The food arrived, and the table fell silent for a moment as everyone savored the first bite. Amelia closed her eyes, enjoying the perfect blend of creamy avocado, runny egg yolk, and crispy toast. It was these simple pleasures that made life in the city so enjoyable. The meal was delicious, and they spent the next few hours eating, reminiscing, and planning future get-togethers.

After brunch, they decided to take a walk along the nearby High Line, an elevated park that offered a serene escape from the urban frenzy. As they strolled, they continued to chat, taking in the greenery and the art installations scattered along the path. The conversation eventually turned to their plans for the upcoming summer.

"I'm thinking of taking a trip to the coast," Amelia mentioned. "There's a small town by the sea that I've heard great things about. I could use some time away from the city."

Her friends nodded in agreement, sharing their own desires for a summer getaway. The idea of a peaceful seaside retreat sounded appealing to all of them.

A few weeks later, Amelia found herself standing on a sandy beach, the gentle waves of the Atlantic Ocean lapping at her feet. The salty sea breeze was a welcome change from the urban air she was used to. She had rented a cozy little cottage overlooking the ocean, and as she stood there, watching the sunset paint the sky with hues of orange and pink, she felt a profound sense of peace.

The sea stretched out endlessly before her, a stark contrast to the confined streets of the city. It was a reminder of the vastness of the world and the endless possibilities that lay ahead. Amelia

took a deep breath, savoring the moment. This trip was exactly what she needed to recharge and gain perspective.

As the sun dipped below the horizon, she thought about her friends back in the city and smiled. She would return to the busy streets and bustling life soon enough, but for now, she was content to listen to the soothing sounds of the sea and enjoy the tranquility of her seaside escape.

Chinese translation

在纽约市一个阳光明媚的星期六早晨，街头巷尾充满了往常的喧嚣与忙碌。空气中回荡着车鸣、人声交谈，还有脚步在街道上节奏分明地敲击的声音。阿梅莉亚的公寓外同样热闹非凡。她站在窗前，手握咖啡杯，静静望着这座活力四射的城市苏醒。她今天的计划是与大学时期的老朋友们一起享用早午餐——这是她们每个月雷打不动的传统，即便每个人的生活都日趋忙碌。

穿过熙攘的人行道时，阿梅莉亚一边走一边想着自己有多么珍惜这些片刻。这座始终在奔跑的城市，是她最能感受到“活着”的地方。她熟练地在人群中穿梭，熟悉的街景与城市的声响让她倍感安心。

她抵达那家安静又别致的小咖啡馆时，朋友们已经等候多时。空气中弥漫着新煮咖啡和烘焙点心的香气。她一进门，便被笑容和拥抱包围。咖啡馆坐落在城市一角较为安静的街区，对她们而言，这里是远离喧嚣的小小绿洲。

她们在熟悉的卡座落座，很快便沉浸在日常的节奏里——闲聊、笑声、彼此的生活点滴。服务员过来点餐时，大家早已迫不及待地选择各自的心头好。阿梅莉亚点了这家招牌的牛油果水波蛋吐司，而朋友们则从松软的煎饼到香气扑鼻的欧姆蛋各取所爱。

当食物上桌，餐桌顿时安静下来，众人沉浸在第一口的美妙滋味中。阿梅莉亚轻闭双眼，感受那奶油般顺滑的牛油果、水波蛋流淌的蛋黄和酥脆吐司交织出的完美口感。正是这些生活中的简单快乐，让城市的日子如此令人着迷。这顿饭美味无比，她们在餐桌前度过了几个小时，一边品尝美食，一边回忆往事、畅谈未来的相聚。

饭后，她们决定去附近的高线公园散步——这是一条建于旧铁路上的空中绿道，宛如都市中的一抹宁静绿意。她们边走边聊，欣赏沿途绿植与艺术装置。谈话渐渐转向了即将到来的夏日计划。

“我在考虑去海边旅行，”阿梅莉亚提道，“听说有一个靠海的小镇非常不错。我感觉自己需要暂时离开一下这座城市。”

朋友们纷纷点头表示认同，也分享了自己对夏日假期的期待。一个宁静的海边小镇，听起来对她们所有人来说都很有吸引力。

几周后，阿梅莉亚果真站在了一片柔软沙滩上，大西洋温柔的海浪轻轻拍打在她脚边。海风带着咸味扑面而来，与她习惯的城市空气截然不同。她租了一间临海的小屋，站在海边，看着夕阳将天际染成橙色与粉红色，心中涌上一种深沉的宁静与满足。

眼前的海洋浩瀚无边，与城市狭窄的街道形成鲜明对比。它仿佛提醒着她世界的广阔与无尽的可能。她深吸一口气，细细品味这一刻。此次旅行，正是她所需要的——充电，重新找回内心的平衡。

当太阳沉入海平线之下，她想到还在城市中的朋友们，嘴角微微扬起。她知道自己很快会回到那喧嚣繁忙的生活中去，但此刻，她只想静静聆听海浪的低语，享受这段属于她自己的宁静海边时光。
