# Supplementary material for: The Effects of (Dis)similarities Between the Creator and the Assessor on Assessing Creativity: A Comparison of Humans and LLMs
Source: J Intell. 2025 Jul 3;13(7):80. doi: 10.3390/jintelligence13070080 (PMC12295035; doi:10.3390/jintelligence13070080)
Supplement: Supplementary file 1 [file jintelligence-13-00080-s001.zip › Supplementary Folder/Stage 1 - Story Collection/Originally Collected Stories/Western AI - ChatGPT/Story 8 - Non-creative.pdf]

English original version

Jessica hurried along the bustling sidewalks of New York City, her footsteps syncing with the cacophony of honking cars, chattering pedestrians, and the distant hum of subway trains. The towering skyscrapers seemed to close in around her, amplifying the frenetic energy of the busy street. She clutched her backpack tightly, weaving through the throngs of people, her mind racing with thoughts of her upcoming presentation. The city had a way of making her feel both insignificant and invincible at the same time, a paradox she had come to embrace over her three years at Columbia University.

Reaching the entrance of her building, she took a deep breath, trying to steady her nerves. Her presentation on sustainable urban development was crucial, not just for her grade but for her future career. Jessica had always been passionate about environmental issues, and this project was her chance to make a real impact. She made her way to the lecture hall, her heart pounding with a mix of excitement and anxiety.

The presentation went smoothly, and by the time she finished, Jessica felt a wave of relief wash over her. Her professor’s approving nod and the applause from her classmates reassured her that she had done well. With her spirits lifted, she decided to treat herself to a nice meal at her favorite café, a small but cozy spot a few blocks away from campus.

As she entered the café, the aroma of freshly brewed coffee and baked pastries greeted her. She found a corner table and ordered her usual: a bowl of creamy tomato soup and a turkey club sandwich. The meal was nothing fancy, but it was comforting and delicious. The first bite of the sandwich was a burst of flavors – the savory turkey, crispy bacon, fresh lettuce, and ripe tomatoes all coming together perfectly. Jessica savored each bite, feeling a sense of contentment settle over her. She watched the world go by outside the café window, the city's rhythm a comforting background to her thoughts.

With her stomach full and her mind at ease, Jessica decided to take a walk to Central Park. The sun was setting, casting a golden hue over the city. She strolled through the park, enjoying the tranquility that contrasted sharply with the city's usual hustle. As she wandered, she found herself thinking about her future, dreaming of the changes she hoped to bring about in the world of urban development.

Lost in her thoughts, Jessica eventually made her way to the edge of the park, where she could see the Hudson River stretching out before her. The sight of the water brought back memories of her childhood summers spent by the sea. The rhythmic sound of the waves and the salty breeze were imprinted in her mind, a soothing reminder of simpler times.

She sat on a bench overlooking the river, watching as the sun dipped below the horizon. The city skyline reflected on the water, creating a mesmerizing blend of lights and colors. Jessica felt a deep sense of peace, as if the sea, even at a distance, had a calming effect on her busy mind.

In that moment, she realized that, like the sea, she needed to find balance in her life. The city’s energy and her own ambitions were important, but so was taking the time to breathe, to enjoy a meal, to appreciate the beauty around her. With renewed resolve, Jessica stood up, ready to face whatever challenges lay ahead, knowing that she could always find solace in the simple pleasures – a tasty meal, a quiet park, and the endless expanse of the sea.

Chinese translation

杰西卡匆匆行走在纽约市熙熙攘攘的人行道上，她的脚步声与汽车喇叭声、行人交谈声和远处地铁的轰鸣声交织在一起，形成了一曲喧闹的城市交响乐。高耸的摩天大楼似乎在她周围合拢起来，更加放大了这条繁忙街道上的紧张气氛。她紧紧抱着背包，在人群中穿梭，脑海里飞快地想着即将到来的演讲。这个城市总能让她感到渺小又无比强大，这种矛盾的感觉，在她哥伦比亚大学三年的学习生活中，已成为她所熟悉的一部分。

到达教学楼门口时，她深吸一口气，试图平复紧张的情绪。她关于可持续城市发展的演讲不仅对她的成绩至关重要，更关乎她未来的职业生涯。杰西卡一直对环保议题充满热情，而这个项目正是她实现影响力的机会。她走进讲堂，心跳加速，兴奋与焦虑交织在一起。

演讲进行得非常顺利，当她讲完时，一股如释重负的感觉涌上心头。教授满意的点头和同学们的掌声让她感到自己表现得不错。心情大好之下，她决定犒赏一下自己，去她最喜欢的咖啡馆吃顿好吃的，这家小而温馨的咖啡馆离校园只有几个街区。

她一走进咖啡馆，便被新鲜咖啡和烘焙面包的香气包围。她找了个角落的座位，点了她的常点：一碗奶油番茄汤和一份火鸡俱乐部三明治。这顿饭虽然不算奢华，却温暖人心、令人满足。三明治的第一口仿佛在味蕾上绽放——咸香的火鸡、酥脆的培根、新鲜的生菜和多汁的番茄完美融合。杰西卡细细品味每一口，感到一种满足感悄然落在心头。她透过咖啡馆的窗户望着外面，城市的节奏成了她思绪中舒缓的背景乐。

吃饱后，心情放松的杰西卡决定去中央公园散步。夕阳正缓缓落下，为城市披上一层金色的光辉。她在公园中漫步，享受着与城市喧嚣形成鲜明对比的宁静。在这片静谧中，她开始思考自己的未来，梦想着自己能在城市发展领域带来的改变。

沉浸在思绪中的她，不知不觉来到了公园的尽头，眼前是浩瀚的哈德逊河。望着水面，她回忆起童年夏天在海边度过的时光。海浪有节奏的声音和咸咸的海风已深深印在她的记忆中，像一剂平静心灵的良药，让她想起那些简单却美好的日子。

她坐在一张面朝河流的长椅上，凝视着太阳沉入地平线的景象。城市的天际线倒映在水面上，光与色交织出迷人的画面。杰西卡感到一股深深的宁静，仿佛即便远离大海，那份海洋般的平和依旧能安抚她忙碌的思绪。

就在那一刻，她意识到，正如大海一样，她的生活也需要找到平衡。城市的活力和她的抱负固然重要，但也不能忽视呼吸、品味一顿饭、欣赏身边美景的时刻。带着新的决心，杰西卡站起身，准备迎接前方的挑战。她知道，只要愿意，她总能在简单的快乐中找到安慰——一顿美味的餐点、一片宁静的公园、还有那无垠的大海。
