# Supplementary material for: The Effects of (Dis)similarities Between the Creator and the Assessor on Assessing Creativity: A Comparison of Humans and LLMs
Source: J Intell. 2025 Jul 3;13(7):80. doi: 10.3390/jintelligence13070080 (PMC12295035; doi:10.3390/jintelligence13070080)
Supplement: Supplementary file 1 [file jintelligence-13-00080-s001.zip › Supplementary Folder/Stage 1 - Story Collection/Originally Collected Stories/Western AI - ChatGPT/Story 9 - Non-creative.pdf]

English original version

On a typical Tuesday morning, Emily hurried down the bustling streets of downtown Chicago. The cacophony of honking cars, the murmur of pedestrians, and the occasional shout from street vendors filled the air. She clutched her tote bag tightly, weaving through the throngs of people, all moving with determined purpose. Emily was on her way to her internship at a marketing firm, a position she had worked hard to secure.

As she reached the towering office building, she paused for a moment to catch her breath and glanced around. The city had a certain charm in its chaos, a rhythm that made her feel alive and part of something bigger. She took a deep breath and pushed open the glass doors, ready to tackle the day ahead.

Midday rolled around, and Emily’s stomach growled in anticipation of lunch. She and her coworker, Sarah, had planned to try a new restaurant that had recently opened a few blocks away. It was known for its eclectic menu and delicious dishes, so they were both excited for a break from the usual sandwich shop.

As they entered the cozy bistro, the rich aroma of spices and freshly cooked food enveloped them. They found a table by the window and eagerly scanned the menu. Emily settled on a plate of creamy pasta with sun-dried tomatoes and basil, while Sarah opted for a gourmet burger with truffle fries. When the food arrived, it did not disappoint. Emily savored each bite, the flavors dancing on her palate. She and Sarah chatted about their weekend plans, the conversation flowing easily over their delightful meal.

Back at the office, the afternoon passed quickly as Emily immersed herself in a new project. By the time she left work, the sun was beginning to set, casting a golden glow over the city. The streets were still busy, but the frenetic energy of the morning had mellowed into a more relaxed hum. She decided to take the long way home, enjoying the evening air and the vibrant cityscape.

As she walked, her phone buzzed with a message from her best friend, Lily. They hadn’t seen each other in weeks, and Lily was suggesting a spontaneous weekend trip. Emily’s eyes lit up at the thought. She had been feeling the weight of her responsibilities lately, and a getaway sounded perfect.

That Friday, Emily and Lily packed their bags and headed to the coast. After a few hours’ drive, they arrived at a quaint seaside town. The salty breeze and the sound of waves crashing against the shore instantly lifted Emily’s spirits. They checked into a charming bed-and-breakfast and spent the evening exploring the town’s boardwalk, filled with quirky shops and seafood stalls.

The next day, they rented bicycles and rode along the coastline, the vast expanse of the sea stretching out beside them. They stopped at a secluded beach, where they laid out a picnic of fresh fruit, cheese, and crusty bread. As they sat on the warm sand, watching the sun dip below the horizon, Emily felt a profound sense of peace. The sea had a way of soothing her soul, its endless horizon reminding her of the limitless possibilities ahead.

Emily returned to the city with a renewed sense of purpose and a lightness in her step. The busy streets of Chicago welcomed her back, but she carried the tranquility of the sea with her, a

reminder that amidst the hustle and bustle, moments of calm and connection were always within reach.

Chinese translation

在一个普通的星期二早晨，艾米丽匆匆走在芝加哥市中心繁忙的街道上。喇叭声此起彼伏，行人的低语和街头小贩的吆喝声交织成一片嘈杂的交响。她紧紧抱着手提包，在熙熙攘攘的人群中穿梭，每个人都带着坚定的目标匆匆前行。艾米丽正前往她实习的营销公司，那是她努力争取来的职位。

当她到达那座高耸的办公楼时，她稍作停顿，喘了口气，环顾四周。这座城市在混乱中自有一种魅力，一种让她感到活着、并成为某个更大整体一部分的节奏感。她深吸一口气，推开玻璃门，准备迎接新的一天。

中午时分，艾米丽的肚子开始咕咕叫，期待着午餐的到来。她和同事莎拉计划去试试新开的一家餐厅，距离公司不过几个街区。这家餐厅以其多样化的菜单和美味佳肴闻名，她们都对这次脱离日常三明治店的午餐充满期待。

一走进这家温馨的小餐馆，香料和新鲜食物的香气扑面而来。她们在窗边找了个座位，兴致勃勃地翻看菜单。艾米丽点了一盘奶油意大利面，搭配风干番茄和新鲜罗勒，莎拉则选择了松露薯条搭配的美味汉堡。菜品上桌后，果然没有让她们失望。艾米丽细细品尝每一口，味道在舌尖跳跃。她和莎拉一边吃一边轻松地聊着周末的计划，整个用餐过程愉快自在。

回到办公室后，下午的时间在艾米丽全身心投入新项目中飞快流逝。下班时，夕阳洒下金色的光辉，城市沐浴在一片温暖中。街道依旧繁忙，但早晨那种紧张忙碌的氛围，已经转为温和平静。她决定绕远路回家，享受这片刻的清新空气和生机勃勃的城市风景。

就在这时，她的手机震动了一下，是她最好的朋友莉莉发来的消息。她们已有好几周没见面了，而莉莉建议来一场说走就走的周末旅行。这个想法令艾米丽眼前一亮。最近她一直感到责任压身，而一次短途旅行正是她所需要的。

到了星期五，艾米丽和莉莉收拾好行李，出发前往海岸。在开了几个小时的车后，她们抵达了一个宁静的海滨小镇。咸咸的海风和海浪拍打岸边的声音立刻让艾米丽的心情轻松了许多。她们在一家迷人的家庭旅馆入住，傍晚则在木栈道上漫步，那里布满了个性小店和海鲜摊位。

第二天，她们租了自行车沿着海岸线骑行，广阔的海洋在一旁延展开来。她们在一处僻静的海滩停下，铺开野餐布，拿出新鲜的水果、奶酪和香脆的面包。在温暖的沙滩上坐下，看着夕阳缓缓沉入海平线，艾米丽感受到一种深深的平静。大海总有一种治愈她心灵的魔力，那无尽的地平线仿佛在提醒她，未来充满无限可能。

回到城市后，艾米丽带着焕然一新的心情踏上熟悉的街道。芝加哥繁忙的节奏重新将她拥入怀中，但她心中依旧留有那片海的宁静。这段回忆提醒着她：即使在喧嚣与奔波之中，总有属于她的片刻安宁与连接，随时都可抵达。
