# Supplementary material for: The Effects of (Dis)similarities Between the Creator and the Assessor on Assessing Creativity: A Comparison of Humans and LLMs
Source: J Intell. 2025 Jul 3;13(7):80. doi: 10.3390/jintelligence13070080 (PMC12295035; doi:10.3390/jintelligence13070080)
Supplement: Supplementary file 1 [file jintelligence-13-00080-s001.zip › Supplementary Folder/Stage 1 - Story Collection/Originally Collected Stories/Western Human Participants/Story 1 - Creative.pdf]

English original version

Hi, I am George. I am a turtle. I think I am lost. A few hours ago I climbed out of my home, and wondered off into the world. The reason I climbed out of the tank I usually live in, is that I think my owner left me there to die. He left a few weeks ago to go on vacation, so I decided to leave too. Now I am standing in a busy street somewhere in some city. I think it is some place called Scheveningen, because I see a rather large sign saying "Vis Scheveningen". Do you know that place? You probably will, but I've never been taken out of my tank before. Finally, I got something to eat. I've never been this happy. I am missing my tank a bit though. I miss floating on the water, and the water's always nice and cool. Suddenly I see a group of people with wet hair and huge almond shaped boards walking down some stairs a few meters in front of me. They must know where to find water, and I decide to follow them. I walk down the stairs, and I notice something is different. I am standing on something soft, warm and comfortable. I look in front of me, and I see miles of sand, hills, and somewhere in the distance, I see water coming towards me in huge waves. I've never seen so much water. I see so many children playing with the sand. They're building castles almost twenty times my size. As I walk towards the water, I see that people are swimming. Some are floating on the water lying on large flamingo's and unicorns. Everything is so peaceful, and finally I reach the water and decide to dive in. The cold water feels amazing around my shell. I feel complete, I've found my new home.

Chinese translation

嗨，我是乔治。我是一只海龟。我觉得我迷路了。几个小时前，我爬出了我的家，闯入了世界。我之所以爬出我通常居住的水箱，是因为我觉得我的主人把我留在那里是要让我死去。他几个星期前去度假了，所以我决定跟着离开。现在我站在某个繁忙的城市街道上。我觉得这个地方叫做 Scheveningen，因为我看到一个相当大的标志写着"Vis Scheveningen"。**你知道那个地方**吗？你可能会知道，但我以前从未被带出过水箱。最后，我找到了一些吃的。我从未这么开心过。不过我有点想念我的水箱。我想念在水上漂浮，水总是那么凉爽。突然，我看到一群头发湿漉漉、手里拿着巨大杏仁形板的人从我前面几米远的楼梯上走下来。他们一定知道哪里能找到水，所以我决定跟着他们。我走下楼梯，注意到有些不同。我站在某种柔软、温暖和舒适的东西上。我朝前看，看到了漫长的沙滩、山丘，远处，我看到水以巨大的波浪朝我涌来。我从未见过这么多水。我看到很多孩子在沙滩上玩耍。他们建造的城堡几乎是我体积的二十倍。当我走向水边时，我看到人们在游泳。有些人躺在大火烈鸟和独角兽上漂浮在水上。一切都是那么宁静，最后我到达水边，决定潜入水中。冰冷的水环绕着我的壳感觉很棒。我感到完整，我找到了我的新家。
