# Supplementary material for: The Effects of (Dis)similarities Between the Creator and the Assessor on Assessing Creativity: A Comparison of Humans and LLMs
Source: J Intell. 2025 Jul 3;13(7):80. doi: 10.3390/jintelligence13070080 (PMC12295035; doi:10.3390/jintelligence13070080)
Supplement: Supplementary file 1 [file jintelligence-13-00080-s001.zip › Supplementary Folder/Stage 1 - Story Collection/Originally Collected Stories/Western Human Participants/Story 10 - Non-creative.pdf]

English original version

It was the beginning of may, and the morning started sunny and bright. Jack had not seen such a blue sky since he left his hometown last September. For the first time in weeks he felt energized and ready to take on the challenges of New York City. Most of the time he still doubts his choice of moving to the big city, but he tries not to think about it. Now that his mother is ill, the most important thing is saving up money to send home to her. So slowly he puts on his suit and tie, grabs a quick bagel from the pantry, and he is on his way. The streets of New York are so busy, yet it can feel so lonely. Jack feels so alone. Suddenly he is stopped in his tracks, he sees the most beautiful girl he has ever laid eyes on. She locks eyes with him, and it is as if time stops. She softly smiles at him, but then quickly disappears back into the crowd. Strangely, he felt that this would not be the last time he saw her. The day moves slowly. Jack has his daily routine of endless corporate paperwork and faxes. As he is making some copies he suddenly hears the clicks of high heels behind him, he slowly turns around and there she is, the girl from the street. He is greeted with the same beautiful smile that took his breath away. “Oh, hello… uhm… do you need the copier?” is all that he could manage to mumble. “Haha, yes, but take your time. What’s your name?” over the next 30 minutes, they go from exchanging formalities to hysterically laughing at each other's jokes, and there is an instant connection. Jane was her name, the most beautiful name of all. The next day they decide to meet for a tasty meal at a cheap but nice restaurant. As they are sat at the table, Jack can’t help but notice strange looks from the people around them. The waiter comes to their table: ‘Hello sir, what would you like to eat?’. Jack replies: ‘Hello, I would like the truffle burger, and my lady would like the lobster ravioli.’ The waiter shot him a confused look, but then nodded his head and went to put his order in. As the night went on, Jack fell more and more in love with Jane. She was the girl of his dreams and he instantly felt as his he would never be lonely again. The next days were amazing, they spent every waking moment together. On friday, Jack proposed they would go to the beach, Jane agreed. It had been a sunny week, so the water was nice and warm. As they went for a swim, they were playfully splashing each other with water. Jack turns around to look at the beach, but when he looks back, Jane has vanished. He starts calling out her name, but she is nowhere to be found. He is frantically looking for her, completely panicked. The lifeguard noticed and jumps in the water. “Sir, what is wrong?”. “Jane, the girl I came with, she.. I turned around for a second and suddenly she was gone!” Against his will, the lifeguard drags him back onto the beach and says “Sir, I saw you arrive, you came here alone”. Slowly the realization kicks in for Jack. Saying nothing more, he picks up his phone and starts dialling a number. “Dr. White… It happened again.”

Chinese translation

五月初，清晨阳光明媚。杰克自去年九月离开家乡以来就没有见过这样蓝的天空。几个星期以来，他第一次感到精力充沛，准备迎接纽约市的挑战。大多数时候，他仍然对自己搬到这个大城市的选择感到怀疑，但他尽量不去想。现在，他母亲生病了，最重要的是要省钱寄回家给她。于是他慢慢地穿上西装和领带，从食品储藏室拿了个速食贝果，就出发了。纽约的街道如此繁忙，却又感觉如此孤独。杰克感到如此孤独。突然间，他被吸引住了，他看到了他见过的最美丽的女孩。她与他对视，仿佛时间停滞了。她对他微笑，但很快又消失在人群中。奇怪的是，他觉得这不会是他最后一次见到她。这一天过得很慢。杰克有着每天无尽的公司文件工作和传真的例行工作。当他在复印一些文件时，突然听到身后高跟鞋的声音，他慢慢转身，她就在

那里，街上的女孩。他看到了那个曾经令他屏住呼吸的美丽微笑。“哦，你好... 嗯...你需要打印机吗？”他只能支支吾吾地说。“哈哈，是的，但不用着急。你叫什么名字？”在接下来的30分钟里，他们从交换礼貌到彼此开玩笑，笑得前仰后合，两人之间有了一种即时的联系。她叫简，是所有名字中最美丽的。第二天，他们决定在一家便宜但不错的餐馆吃顿美味的饭菜。当他们坐在桌子旁时，杰克不禁注意到周围人的奇怪眼光。服务员来到他们的桌子：“您好先生，您想吃什么？”杰克回答：“你好，我想要松露汉堡，我的女士想要龙虾馄饨。”服务员一脸困惑地看着他，然后点了点头，去点他的餐了。随着夜晚的深入，杰克越来越爱上简。她是他梦中的女孩，他立刻觉得自己再也不会孤独了。接下来的几天很美妙，他们每一个清醒的时刻都在一起。周五，杰克建议他们去海滩，简同意了。这是一个阳光明媚的一周，所以海水温暖宜人。当他们去游泳时，他们互相打着水玩闹。杰克转身看着海滩，但当他回头时，简已经消失了。他开始呼喊她的名字，但她却不见了。他焦急地四处寻找，完全陷入恐慌之中。救生员注意到了，跳进了水里。“先生，怎么了？”“简，我跟她一起来的女孩，她...我只是转身了一秒钟，突然她就不见了！”尽管不情愿，救生员还是把他拖回了海滩，并说：“先生，我看见你来的时候，你是一个人来的。”慢慢地，杰克开始意识到发生了什么。他什么也没有说，只是拿起手机开始拨打一个号码。“怀特博士...
