# Supplementary material for: The Effects of (Dis)similarities Between the Creator and the Assessor on Assessing Creativity: A Comparison of Humans and LLMs
Source: J Intell. 2025 Jul 3;13(7):80. doi: 10.3390/jintelligence13070080 (PMC12295035; doi:10.3390/jintelligence13070080)
Supplement: Supplementary file 1 [file jintelligence-13-00080-s001.zip › Supplementary Folder/Stage 1 - Story Collection/Originally Collected Stories/Western Human Participants/Story 11 - Non-creative.pdf]

English original version

Traffic is hell. My knees drum a quick rhythm as I inch the car another small step forward. Next to me, my mom is typing away at her phone, swearing quietly under her breath. “If this continues, we won’t make it on time,” she says, throwing a hurried look out the front window. We’re still at least half an hour of normal traffic flow away from the Cologne airport. Over the break, we visited friends of the family in Germany, but with the end of summer quickly approaching, it is time to head back home. If traffic would allow us, that is. “We still have time,” I sooth, trying to remember if there is another route to the airport, “we planned in two hours of buffer time. As long as we’re out of here in the next 1.5 hours, we should be fine. Can you google whether we can take a different street?” The sun is beating down on us, warming the car unbearably, I’m hungry and I have to pee. All in all, not the greatest way to start the day. In the end, mom did find a quicker route, and we managed to be on time, with another hour to spare. “First things first, food!” I declare, my stomach rumbling it’s assent. Mom laughs but doesn’t disagree, and we end up sitting down in a small Caffe, mom ordering a muffin and espresso, and me a hot chocolate and bread roll with cheese and eggs. The first bite is heavenly, and I wash it down with creamy chocolate. Leaning back in my chair, I close my eyes and take a moment to savor the taste, quite honestly a little surprised that airport food could be that good. Or maybe I was just that hungry. Next to us is a huge window – a wall made of glass, really – and it gives us a perfect view of the track outside, planes taking off with a roar of the engines. So much concrete is making my eyes hurt. I miss home; out little cottage in the suburban neighborhood, less than ten minutes bike ride to the sea. On good days, me and my friends used to climb all the way to the roof with blankets and food, and we’d watch the sea, bath in the sun and enjoy the weekends. 90 minutes later we’re sitting on the plane back home, my mom asleep only 10 minutes into the flight. I put in my headphones and take out my book. It is the second one in the series, and I’ve been dying to find out how the story continues, after the first book ended in a massive cliffhanger, the heroin sneaking into the lair of the dragon to rescue the prince. Being engrossed in my book, I barely notice when we start to descent, only putting the book way when my mom directs my attention outside. Beneath us, the sea is blue and sparkling, reaching as far back as I can see; a vast emptiness of rich color. My heart soars, and I press my face into the window. We’re finally home.

Chinese translation

交通真是一团糟。我的膝盖轻击着方向盘，车子又往前挪了一小步。我妈妈坐在我旁边，手指飞快地敲击着手机屏幕，嘴里轻声咒骂着。她抬头急急地朝前看了一眼，“**如果**这样下去，我们就赶不上了。”**我**们距离科隆机场至少还有半个小时的正常交通流程。在假期期间，我们去德国拜访了家人的朋友，但是随着夏天即将结束，是时候回家了。只要交通允许的话，这是。“**我**们还有时间的，”**我安**抚道，试图回忆是否有其他路线可以到达机场，“**我**们计划了两个小时的缓冲时间。只要在接下来的一个半小时内出发，我们应该没问题。你能查一下我们能不能走另外一条路吗？”**太阳**炽热地照在我们身上，让车内变得难以忍受，我又饿又想上厕所。总的来说，这不是开始一天的最佳方式。最后，妈妈找到了一条更快的路线，我们成功地准时出发，还多了一个小时的时间。“**首先得吃点**东西！”**我宣布道，我的胃咕咕叫着**。妈妈笑了，但没有反对，我们最终坐在了一家小咖啡馆里，妈妈点了一个松饼和浓缩咖啡，我点了一杯热巧克力和一块带有奶酪和鸡蛋的面包卷。第一口吃下去是天堂般的享受，我用浓郁的巧克力冲淡了

口感。我靠在椅子上闭上眼睛，花了一会儿时间来品尝这种味道，相当诚实地有点惊讶于机场食物居然如此美味。或许我只是太饿了吧。我们旁边是一个巨大的窗户——实际上是一面玻璃墙——让我们可以完美地看到外面的跑道，飞机轰鸣着起飞。这么多的混凝土让我的眼睛有些疼。我想念家；在郊区小屋，离海不到十分钟的自行车路程。在好天气里，我和朋友们常常爬到屋顶，铺上毯子，带上食物，然后我们就可以看着海，沐浴在阳光下，享受周末。90分钟后，我们坐在飞回家的飞机上，我的妈妈在飞行才10分钟就睡着了。我戴上耳机，拿出我的书。这是系列中的第二本，我迫不及待地想知道故事的发展，因为第一本书以一个巨大的悬念结尾，女主角潜入龙的巢穴营救王子。我专心读着书，几乎没有注意到我们开始下降，只有当妈妈引起我的注意力时，我才把书放开。在我们下面，大海蓝蓝的，闪闪发光，一直延伸到我能看到的地方；一片丰富色彩的广阔空间。我的心飞扬起来，我把脸贴在窗户上。我们终于回家了。
