# Supplementary material for: The Effects of (Dis)similarities Between the Creator and the Assessor on Assessing Creativity: A Comparison of Humans and LLMs
Source: J Intell. 2025 Jul 3;13(7):80. doi: 10.3390/jintelligence13070080 (PMC12295035; doi:10.3390/jintelligence13070080)
Supplement: Supplementary file 1 [file jintelligence-13-00080-s001.zip › Supplementary Folder/Stage 1 - Story Collection/Originally Collected Stories/Western Human Participants/Story 12 - Non-creative.pdf]

English original version

It was a Monday morning. Thomas woke up in his family apartment in New York, checked his alarm clock and realised he had slept through his alarm. He rushed into his clothes, ate a quick breakfast, and went off to work. Driving through the busy streets of New York was often tedious, but he managed to get to his office on Wall Street on time. His colleagues were glad to see him, and Thomas discussed his weekend with his friend Joe. Joe had gone skydiving on Saturday and described the exhilarating experience. After a few hours at work (he worked as an accountant), Joe suggested to go get lunch together. So Thomas and Joe went off to a local Italian lunchroom. Thomas had only had a small breakfast, so he was starving. They had delicious pasta salads and sandwiches. Completely stuffed, they returned to the office and worked for a couple more hours. Thomas enjoyed his work very much and had been in the job for over 5 years. After a short meeting with several colleagues, Thomas was already free at 3 in the afternoon. He picked up his two kids, Julian and Norah, from school, and drove home. His wife Melissa was already home too and had just made coffee. They drank coffee together and talked with the kids about what they had learnt in school that day. Julian had learnt about dinosaurs and was absolutely fascinated by it. Norah, who was a bit younger, had done a lot of drawing at school that day, and presented a drawing of the family. The drawing was splendidly colourful but completely out of proportion: Thomas' head was absolutely oversized, and he was about twice as large as the rest of the family members. But Thomas and Melissa told her it was beautiful, and that made Norah happy. Since Thomas was free from work early, they had some more time before dinner to spend together. Since it was a sunny day, they decided to drive off to a beach at the Lower Bay. They spent two hours at the beach. Julian had recently learnt how to swim, and Thomas constantly had to keep him away from the deeper sea, because Julian kept wanting to go further. Norah instead, walked through the water and laying on the beach with her mother. After a fun afternoon at the beach, they all returned home and had dinner together. Melissa had made delicious lasagna, Thomas' favourite dish. Afterwards, they watched a movie together, and brought the kids to bed. Thomas and Melissa were happy with their family life and their lovely children: and they were already awaiting a third! They headed to bed. And Thomas made sure to turn the volume of his alarm clock all the way up for the next morning...

Chinese translation

星期一早晨。托马斯醒来，发现自己已经睡过了闹钟。他匆忙穿好衣服，吃了一顿快速的早餐，然后去上班。穿行在纽约繁忙的街道上通常很乏味，但他还是设法准时到达了他在华尔街的办公室。同事们见到他很高兴，托马斯和他的朋友乔讨论了周末的情况。乔周六去跳伞了，并描述了令人兴奋的体验。在办公室工作了几个小时后（他是一名会计），乔建议一起去吃午饭。于是托马斯和乔去了当地的意大利午餐厅。托马斯只吃了一点早餐，所以他饿极了。他们吃了美味的意大利面沙拉和三明治。吃得饱饱的，他们回到了办公室，又工作了几个小时。托马斯非常享受自己的工作，并且已经在这份工作上工作了五年多。在和几个同事短暂会议后，托马斯下午三点就自由了。他接回了两个孩子，朱利安和诺拉，放学回家。他的妻子梅丽莎也已经回家了，刚刚煮好了咖啡。他们一起喝咖啡，和孩子们聊了他们今天在学校学到的东西。朱利安学到了关于恐龙的知识，并且对此非常着迷。年龄稍小的诺拉在学校画了很多画，还拿出了一幅全家福。这幅画色彩绚丽，但比例完全失调：托马斯的头绝对是超大的

，而且他比其他家庭成员大了一倍。但托马斯和梅丽莎告诉她，这是美丽的，这让诺拉很高兴。由于托马斯下午提早下班，他们有更多的时间可以一起度过。**由于天气晴朗，他**们决定开车去下湾的海滩。他们在海滩度过了两个小时。朱利安最近学会了游泳，托马斯不得不不停地阻止他靠近更深的海域，因为朱利安总是想游得更远。而诺拉则和她妈妈一起在水里走动，并躺在沙滩上。在海滩上度过了一个愉快的下午后，他们回到家，一起吃晚饭。梅丽莎做了美味的千层面，是托马斯最喜欢的菜。晚饭后，他们一起看了一部电影，然后把孩子们哄睡了。托马斯和梅丽莎对他们的家庭生活和可爱的孩子们感到满意：他们已经等待着第三个孩子的到来！他们去睡觉了。托马斯确保第二天早上把闹钟的音量调到最大...
