# Supplementary material for: The Effects of (Dis)similarities Between the Creator and the Assessor on Assessing Creativity: A Comparison of Humans and LLMs
Source: J Intell. 2025 Jul 3;13(7):80. doi: 10.3390/jintelligence13070080 (PMC12295035; doi:10.3390/jintelligence13070080)
Supplement: Supplementary file 1 [file jintelligence-13-00080-s001.zip › Supplementary Folder/Stage 1 - Story Collection/Originally Collected Stories/Western Human Participants/Story 13 - Non-creative.pdf]

English original version

The story begins in a busy street in the big city of New York. Marcia was a young woman who moved to the big town to achieve her dreams as an actor. She was dedicated to enroll in numerous workshops across the city as she believed to gain the highest concentration of competence in a city like New York. Marcia's move to New York was mostly done by impulses and this she is living in a run down apartment. If you ask her, she will tell you that it's the charme and according to her fantasies of am up-coming actor to endure these circumstances and do build character. She was rather naive and full of hope when it came to her dream. As Marcia made her way to her new workshop, she took the train in which she could observe many eccentric people, as she thought to herself. She saw many drag queens who had to take the public transportation as their work day has concluded in the very same morning her work day would only start to begin. Marcia felt like an outsider among the people she observed, she saw them as a big group of people belonging to this place and she started to doubt herself and her impulse decision to taker her dream seriously. When she left the train station to walk to the theatere where she was going to be studying at for a while, she tried to take everything in she could, it was New York after all and she was making sure to take everything in when she could. While she was taking pictures, Marcia noticed a pizza place, she believed it was from a show but she couldn't really think of which one. She carelessly entered as she was ready to treat herself to a tasty meal, pizza was something New York was known for, she couldn't pass up this opportunity. She ordered a slice, she was taken back by the business and it's practices as the place was busy and hectic even at such an early time. When she made it out of the pizza shop, she went through a dark alleyway to get to the theatre. As she made her way to her designated destination, Marcia grew more uncomfortable and continously checked her phone if she's following the right instructions. This couldn't be. When she heard a loud bang behind her, she did not even look back and burst into running. Suddenly mist seemed to surround her from all corners. At this time, Marcia dropped her pizza on the filthy floor as she observed what was going on. Marcia witnessed as the area around her turned to a black landscape, gone was the alleyway and the noise of the streets and now only a void was surrounding her. It seemed that this world was strangely familiar to her. She wandered around, not finding anyone else but being stuck in a limbo it seems. Marcia grows more scared and frightened but she can't shake off this feeling of familarity, as she reminisced of this feeling she spots someone at a distance. Marcia makes a run for it and recognizes her brother, she catches up to him while she was screaming for his name. As she finally reaches for him, she falls down witnesses him walking into the see. Marcia distraught by what's happening weaps and passes out in pain. She feels herself waking, she's back at home, with a vlog from a student the theater of New York playing. Her mom enters her room and asks "Why are you not getting ready for the funeral, it's your brother get it together."

Chinese translation

故事始于纽约这个繁忙的大城市的一条街道上。玛西娅是一个年轻的女子，她搬到这个大城市是为了实现自己作为演员的梦想。她致力于参加城市各地的许多工作坊，因为她相信在像纽约这样的城市中能够获得最高水平的竞争力。玛西娅搬到纽约的决定大多是出于冲动，所以她住在了一间破旧的公寓里。如果你问她，她会告诉你这是魅力所在，根据她对即将成为一名演员的幻想，忍受这些环境会培养性格。当谈到她的梦想时，她相当天真和充满希望。当玛西娅前往她的新工作坊时，她搭乘了火车，可以观察到许多古怪的人，她认为自己是。她看到了许多变装皇后，因为她想，这些人的工作日已经在同一天的早晨结束了，而她的工作日刚刚开始。

玛西娅觉得自己是观察到的人群中的一个局外人，她把他们视为这个地方的一大群人，她开始怀疑自己和她冲动地认真对待自己的梦想的决定。当她离开火车站步行去剧院时，她试图尽可能地吸收一切，毕竟这是纽约，她要确保尽可能多地吸收。当她在拍照时，玛西娅注意到了一个披萨店，她相信这是一个节目，但她无法想起是哪一个。她毫不在意地走进去，因为她准备好给自己来一顿美味的餐点，披萨是纽约的特色之一，她不能错过这个机会。她点了一块披萨，她被这家生意兴隆和它的经营方式震撼了，因为即使在这样一个早上，这个地方也是忙碌和混乱的。当她走出披萨店时，她穿过一条黑暗的小巷去往剧院。当她朝着指定的目的地前进时，玛西娅变得更加不安，不停地查看手机以确保她是否按照正确的指示行事。这不可能发生。当她听到背后传来一声巨响时，她甚至没有回头，就开始奔跑。突然间，薄雾似乎从四面八方围绕着她。这时候，玛西娅把披萨掉在了肮脏的地板上，因为她观察到了周围发生的事情。玛西娅目睹了她周围的地区变成了一片黑色的景观，小巷和街道的噪音消失了，现在只剩下了一片虚无。这个世界似乎对她来说有些奇怪的熟悉。她四处游荡，找不到其他人，只是被困在了一个空间里。玛西娅越来越害怕和恐惧，但她无法摆脱这种熟悉的感觉，因为她回忆起这种感觉，她在远处看到了一个人。玛西娅冲过去，认出了她的兄弟，她边跑边喊他的名字。当她最终赶到他身边时，她跌倒了，目睹他走进了大海。玛西娅被所发生的事情弄得心烦意乱，哭泣着痛苦地昏了过去。她感到自己醒来了，她回到了家里，屏幕上播放着纽约剧院的学生的vlog。她妈妈走进她的房间，问："你为什么还没准备好去葬礼，那是
