# Supplementary material for: The Effects of (Dis)similarities Between the Creator and the Assessor on Assessing Creativity: A Comparison of Humans and LLMs
Source: J Intell. 2025 Jul 3;13(7):80. doi: 10.3390/jintelligence13070080 (PMC12295035; doi:10.3390/jintelligence13070080)
Supplement: Supplementary file 1 [file jintelligence-13-00080-s001.zip › Supplementary Folder/Stage 1 - Story Collection/Originally Collected Stories/Western Human Participants/Story 2 - Creative.pdf]

English original version

In the middle of the big city, where the streets are always crowded and noisy, there's a little café that feels like a cozy hideout. I, a 19-year-old psychology student, often escape there from my hectic university life. One super busy day, with my head spinning from all the assignments and exams, I stumbled upon this café. It seemed like a cozy oasis compared to the chaos outside, so I decided to check it out. As I walked in, the smell of fresh coffee and baked goods instantly made me feel better. I found a quiet spot to sit, away from the hustle and bustle, and took a deep breath, feeling grateful for the peace and quiet. Looking at the menu, I spotted something familiar and comforting, avocado toast with eggs. It's a simple dish, but it always puts a smile on my face. I ordered it without hesitation and waited eagerly for it to arrive. When my meal came, it looked even better than I imagined. The avocado was perfectly ripe, and the eggs were cooked just right. I took a bite and was instantly transported to a happy place. The creamy avocado and runny eggs on crunchy toast were a match made in heaven. With each bite, I could feel the stress melting away, replaced by a warm feeling of contentment. It was like a little slice of happiness during a crazy day. After finishing my meal, I felt energized and ready to take on the world again. But instead of rushing back into the chaos of the city, I decided to take a leisurely stroll by the sea. Standing by the water, with the salty breeze on my face and the sound of waves crashing against the shore, I felt a sense of peace wash over me. The vastness of the ocean seemed to put things into perspective, reminding me that there's so much more to life than just deadlines and exams. As I watched the sun set over the horizon, painting the sky in shades of orange and pink, I felt grateful for moments like these – moments of quiet reflection and appreciation for the beauty of the world around us. And as I walked back to the city, feeling refreshed and renewed, I made a promise to myself to seek out these moments of peace and tranquility more often, knowing that they are the key to staying sane in a busy world.

Chinese translation

在这座繁华的城市中心，街道上总是挤满了人，嘈杂不止。但有一家小咖啡馆却让人感觉像是一个舒适的藏身之地。我，一个19岁的心理学学生，经常逃到这里，远离我忙碌的大学生活。有一天，我的头脑被所有的作业和考试弄得眩晕不已，我偶然发现了这家咖啡馆。与外面的混乱相比，它看起来像是一个温馨的绿洲，所以我决定进去看看。当我走进去时，新鲜咖啡和烘焙的美食的香气立刻让我感觉好多了。我找到一个安静的地方坐下，远离喧嚣，深深地吸了一口气，为平静感到感激。看着菜单，我看到了一个熟悉而令人安心的东西，鳄梨吐司配蛋。这是一道简单的菜，但它总是能让我笑容满面。我毫不犹豫地点了它，并迫不及待地等待着。当我的餐点送来时，它看起来甚至比我想象的还要好。鳄梨刚好成熟，鸡蛋煮得恰到好处。我咬了一口，立刻被带到了一个快乐的地方。酥脆的吐司上的奶油鳄梨和流动的鸡蛋简直是天作之合。每一口都能感觉到压力在消失，被一种温暖的满足感所取代。在这个疯狂的一天里，它就像是一小块快乐。吃完我的餐后，我感到充满活力，准备再次面对世界。但我没有急着回到城市的混乱中，而是决定悠闲地在海边散步。站在水边，感受着咸咸的海风拂过脸庞，听着波浪拍打岸边的声音，我感到一种平静的感觉油然而生。广阔的海洋似乎让人对事物有了新的认识，提醒我生活中远不止是截止日期和考试。当我看着太阳落下地平线，天空被橙色和粉红色的色彩涂抹时，我为这样的时刻感到感激——静静地反思，欣赏着我们周围世界的美丽。当我

走回城市时，感觉焕然一新，我对自己做出了一个承诺，要更经常地寻找这样的平静和宁静，因为我知道它们是在这个繁忙的世界中保持理智的关键。
