# Supplementary material for: The Effects of (Dis)similarities Between the Creator and the Assessor on Assessing Creativity: A Comparison of Humans and LLMs
Source: J Intell. 2025 Jul 3;13(7):80. doi: 10.3390/jintelligence13070080 (PMC12295035; doi:10.3390/jintelligence13070080)
Supplement: Supplementary file 1 [file jintelligence-13-00080-s001.zip › Supplementary Folder/Stage 1 - Story Collection/Originally Collected Stories/Western Human Participants/Story 3 - Creative.pdf]

English original version

Hello dear reader, I will tell a story about Maria de Groot. You don't know Maria yet, that's not possible because Maria has been locked up all her life. Maria cannot tolerate sunlight, she is allergic to it and therefore very afraid. Yet Maria does not have a boring life, because she lives in a very high building above a very busy street in New York City. Her parents have made a gate for Mary in the sky, through which she can walk to see the people below and around her. Maria is therefore somewhat familiar with the outside world, even though she has never been there... Maria turned 18 this year, after which her parents gave her the opportunity to go outside a certain amount, on the condition that she does not will be outside for more than an hour. She must wear covering clothing and keep out of sunlight as much as possible. Maria is tense but also extremely enthusiastic about her new adventure where she hopes to make many new friends. The evening before, she goes out in the car with her driver to further explore the city before her big day. She visits many major roads, where it is never quiet in New York, and looks ahead to where she can best go during the day. She has already planned her entire route, but hopes for some exciting moments tomorrow that will allow her to experience some unexpected moments. The day breaks and at 12 o'clock Maria steps out of the door, her mother is crying, her father looks especially worried. Maria immediately heads towards the streets where she had been last night. She sees a boy standing there, all alone. She approaches him and greets him. He looks around in surprise and a bit of alarm. Maria asks him to eat a meal together, not realizing that it is strange if you don't know each other. The young man thinks differently and drags Maria into a white van that has just arrived behind them. He kidnapped Maria. For a long time, Maria is mostly afraid, until the boy arrives with a meal for her, then she feels fine. She sees it as an adventure, making new friends. When she finally regains her sight because the door of the bus is thrown open, the boy is standing in front of her again. He tells her that her parents lied to her, that she is not allergic to the sun and does not have to be afraid of him. Maria is surprised and does not believe what he tells her. Maria therefore decides to kill the boy and the driver of the car with the knife she brought with her and later dump them in the sea. That same evening she tells her parents about her adventures and suggests they go outside again for an hour next week. Maria isn't that smart.

Chinese translation

亲爱的读者，我要讲一个关于玛丽亚·德·格鲁特的故事。你还不认识玛丽亚，这是不可能的，因为玛丽亚一生都被关在里面。玛丽亚无法忍受阳光，她对阳光过敏，因此非常害怕。然而，玛丽亚的生活并不乏味，因为她住在纽约市一条非常繁忙的街道上的一栋非常高的建筑物里。她的父母为玛丽亚在天空中建造了一扇门，通过这扇门，她可以走出去看下面和周围的人。因此，玛丽亚对外界有些熟悉，尽管她从未去过那里... 玛丽亚今年已经18岁了，之后她的父母给了她出门一定的机会，条件是她不能在外面呆超过一个小时。她必须穿着遮盖衣服，并尽量避开阳光。玛丽亚感到紧张但也非常兴奋，她希望在新的冒险中能结交到许多新朋友。前一天晚上，她和司机一起乘车进一步探索了她眼前的这座城市。她参观了许多主要的道路，在纽约从不会安静，然后想象着第二天她应该去哪里。她已经计划好了整个路线，但希望明天能有一些令人兴奋的时刻，让她体验一些意外的时刻。天亮了，中午12点，玛丽亚走出门，她的母亲在哭泣，她的父亲看起来特别担心。玛丽亚立刻朝着昨晚她去过的街道走去。她看到一个男孩站在那里，孤零零的。她走上前去和他打招呼。他惊讶地四处张望，有些惊慌。玛丽亚邀请

他一起吃饭，没有意识到如果你们彼此不认识，这样做是很奇怪的。这位年轻人的想法有所不同，他将玛丽亚拖进了刚到达他们身后的一辆白色货车里。他绑架了玛丽亚。很长一段时间，玛丽亚都感到恐惧，直到男孩给她带来了一顿饭，然后她感觉好多了。她将这看作是一次冒险，结交新朋友。当她最终恢复视力时，因为车门被推开了，男孩再次站在她面前。他告诉她她的父母欺骗了她，她并不对阳光过敏，也不必害怕他。玛丽亚感到惊讶，不相信他告诉她的话。因此，玛丽亚决定用她带来的刀杀死男孩和车上的司机，然后把他们扔进海里。同一天晚上，她告诉她的父母她的冒险经历，并建议下周再一次外出一个小时。玛丽亚并不聪明。
