# Supplementary material for: The Effects of (Dis)similarities Between the Creator and the Assessor on Assessing Creativity: A Comparison of Humans and LLMs
Source: J Intell. 2025 Jul 3;13(7):80. doi: 10.3390/jintelligence13070080 (PMC12295035; doi:10.3390/jintelligence13070080)
Supplement: Supplementary file 1 [file jintelligence-13-00080-s001.zip › Supplementary Folder/Stage 1 - Story Collection/Originally Collected Stories/Western Human Participants/Story 4 - Creative.pdf]

English original version

Today I'm going to tell you something about my past few days. it all started on a thursday. I was in town with my friend Henk. Henk and I went to the center to have lunch. We came to the center by metro, but on the way we met artists at the metro station. We saw dancers, singers and instrument players. One of the artists played the saxophone. I recognized the artist with the saxophone. I didn't know anything I recognized him from. It was a man of above average age and he was bald. I asked Henk if he knew him but he didn't. So I thought hard and then I remembered. It was my old music teacher from high school. We spoke briefly and told him that we would come visit each other later in the city. As we exited the subway and walked along a busy street, a taxi passed by. There was a man in the taxi who spoke to us and said that we had to be careful where we walk in a city like Lisbon. Maybe I forgot to mention it, but we were in Lisbon. After the taxi almost ran us over, we walked to the center. When we arrived at the center, there was a lot of choice for lunch. We walked past many restaurants but were not blown away by anything. Finally, after being accosted by many men who wanted to charm us in, we finally went to a rooftop café in the center. Henk and I had delicious burgers there. Henk had a cheese burger with extra pickle and I had a chicken burger. My burger had avocado on it and I'm not really crazy about that but I ate it and it was very tasty. When we finally finished eating and enjoying the view we saw shining lights in the distance. It was like there was a festival somewhere so we were curious. We also heard many cars honking. Henk asked the bartender what was going on, the bartender indicated that it was the final of the local football tournament so the city was in commotion. After a delicious meal we went downstairs. Henk wanted to drink cocktails so we looked for a nice terrace where we could drink them cheaply. So we walked through the center and then we suddenly came across a large procession of rioting young people who were celebrating that their favorite football club won. This looked fun at first until some fireworks were set off and it got very close. Then we walked quickly and arrived at the beach where we finally sat down and had cocktails. We had a view of the sea and, according to Henk and I, it was a nice end to our day in Lisbon. And so the end of our story about our day in Lisbon.

Chinese translation

今天我要告诉你一些我过去几天的事情。一切都始于一个星期四。我和我的朋友亨克在城里。我和亨克去市中心吃午饭。我们乘地铁来到市中心，但在路上我们在地铁站遇到了艺术家。我们看到了舞者、歌手和乐器演奏者。其中一个艺术家吹奏着萨克斯管。我认出了那个吹萨克斯管的艺术家。我对他的熟悉感来自哪里我不知道。他是一个年龄超过平均水平的人，他是个秃头。我问亨克是否认识他，但他不认识。所以我努力回忆，然后我想起来了。那是我高中的音乐老师。我们简短地交谈了一下，并告诉他我们会在城里稍后拜访彼此。当我们走出地铁站，沿着繁忙的街道走时，一辆出租车驶过。出租车里有个人对我们说，在里斯本这样的城市里，我们要小心走路。也许我忘了提一下，但我们在里斯本。在出租车几乎撞到我们之后，我们走到了市中心。当我们到达市中心时，午餐的选择很多。我们走过很多餐馆，但没有被任何东西震撼到。最后，在被很多男人搭讪后，他们想要魅惑我们进去，我们终于去了市中心的一个屋顶咖啡馆。亨克和我在那里吃了美味的汉堡。亨克点了一份芝士汉堡加额外的

泡菜，我点了一个鸡肉汉堡。我的汉堡上有鳄梨，我对此并不是很喜欢，但我吃了它，味道很好。当我们终于吃完并享受了美景时，我们看到远处有闪亮的**灯光。就像某个地方正在**举办节日一样，所以我们很好奇。我们也听到很多汽车的喇叭声。亨克问了酒吧里的调酒师发生了什么事，调酒师表示这是当地足球比赛的决赛，所以整个城市都在骚动。吃过美味的餐后，我们下楼了。亨克想要喝鸡尾酒，所以我们找了一个可以便宜喝酒的漂亮露台。所以我们穿过市中心，突然遇到了一大群年轻人聚集在一起庆祝他们最喜爱的足球俱乐部获胜的游行。起初这看起来很有趣，直到有些烟花被点燃，离我们很近。然后我们赶快走开，来到了海滩，最后坐下来喝鸡尾酒。我们能看到大海，根据亨克和我，这是我们在里斯本的一天的美好结束。这就是我们在里斯本的一天的故事的结局。
