# Supplementary material for: The Effects of (Dis)similarities Between the Creator and the Assessor on Assessing Creativity: A Comparison of Humans and LLMs
Source: J Intell. 2025 Jul 3;13(7):80. doi: 10.3390/jintelligence13070080 (PMC12295035; doi:10.3390/jintelligence13070080)
Supplement: Supplementary file 1 [file jintelligence-13-00080-s001.zip › Supplementary Folder/Stage 1 - Story Collection/Originally Collected Stories/Western Human Participants/Story 5 - Creative.pdf]

English original version

It was summer and I just arrived in Barcelona, one of my favorite cities to spend my summer vacation in. I went together with two friends, who also like Barcelona a lot. We dropped our suitcases at the hotel, and went directly to the supermarket to buy some groceries for the week we would spend there. It was late in the afternoon, which meant that people were going home after work. The streets were very busy, but fortunately the supermarket was just around the corner. After we bought some groceries, we saw a cute little coffeeshop. We went inside and bought ourselves a delicious, iced coffee. After we finished our iced coffees we decided to return to the hotel, and sleep for a bit, since we were very tired from traveling. About two hours later, we woke up, and we were a bit hungry. So we took a shower, did our make-up, put on some cute clothes and went into the centrum to find a nice place to eat something. We found a very cute place, just next to a fountain. In the background a violist was playing some beautiful classical music. We all ate some paella, since it is one of our favorite meals. When we finished our meals, we decided to take a walk along the streets, hoping we would find some cute boutiques to buy some accessories. Many boutiques later, we found a busy terrace just near our hotel, and we decided to do some drinks before going back to our hotel. We met some lovely people there, which were also people who were staying at our hotel. We talked a lot about traveling and vacations, since we all had a passion for those subjects. We talked for a very long time, and eventually decided to drop our purchases at the hotel, and go out in the city. We found a nightclub where they played some great R&B music, which happens to be our favourite music genre. We danced all night, had a few more drinks, met even more fun and sweet people and took many pictures. At the end of the night, we decided to go back to our hotel. We came back, cleaned ourselves and went to bed, since we were all exhausted. The day after, we woke up and talked about everything that happened the night before. We looked at the photos, taken the night before. We had a laugh about everything that happened and we made ourselves ready to go to the beach. It was a really warm and sunny day, so we had to use a lot of suncream. We swam a lot in the sea, we even saw many fishes. I called with my family, to tell them how much fun I had there. I started reading a new book at the beach, but I fell asleep since the book was quite boring. I remember how pleasant it felt to lie there on the beach, with the heat of the sun. I would love to go back some day.

Chinese translation

那是夏天，我刚到巴塞罗那，这是我最喜欢度暑假的城市之一。我和两个也喜欢巴塞罗那的朋友一起来的。我们把行李放在了酒店，直接去超市买了一周的食物。那时已经下午很晚了，意味着人们下班回家了。街道非常繁忙，但幸运的是超市就在附近。买了些食物后，我们看到了一家可爱的小咖啡店。我们走进去，买了一杯美味的冰咖啡。喝完冰咖啡后，我们决定返回酒店休息一会儿，因为长途旅行让我们感到非常累了。大约两个小时后，我们醒来，有点饿了。所以我们冲了个澡，化了妆，穿上了一些可爱的衣服，去市中心找个地方吃点东西。我们找到了一个非常可爱的地方，就在一个喷泉旁边。背景里，有一位小提琴手正在演奏一些美妙的古典音乐。我们都吃了些西班牙海鲜饭，因为这是我们最喜欢的食物之一。吃完饭后，我们决定沿着街道散步，希望能找到一些可爱的精品店买些配饰。经过许多精品店之后，我们找到了一个繁忙的露台，就在我们的酒店附近，我们决定在返回酒店之前喝点东西。我们在那里遇到了一些可爱的人，他们也住在我们的酒店。我们谈了很多关于旅行和度假的话题，因为我们都对这些话题充满了热情。我们聊了很长时间，最终决定把购物的东西放在酒店，然后去城里逛逛

。我们找到了一个夜总会，他们播放一些很棒的R&B音乐，这恰好是我们最喜欢的音乐类型。我们整晚跳舞，喝了更多的酒，认识了更多有趣和友善的人，并拍了许多照片。在夜晚结束时，我们决定回酒店。我们回到酒店，洗了个澡，上床睡觉，因为我们都筋疲力尽了。第二天，我们醒来，谈论了前一晚发生的一切。我们看了前一天晚上拍的照片。我们笑了笑关于发生的一切，准备去海滩。那是一个非常温暖和阳光明媚的一天，所以我们不得不涂很多防晒霜。我们在海里游了很多，甚至看到了许多鱼。我给家里打了电话，告诉他们我在那里玩得多么开心。我在海滩上开始看一本新书，但由于书相当无聊，我睡着了。我记得躺在沙滩上感觉是多么的愉快，享受着阳光的热情。我希望有一天能再回去。
