# Supplementary material for: The Effects of (Dis)similarities Between the Creator and the Assessor on Assessing Creativity: A Comparison of Humans and LLMs
Source: J Intell. 2025 Jul 3;13(7):80. doi: 10.3390/jintelligence13070080 (PMC12295035; doi:10.3390/jintelligence13070080)
Supplement: Supplementary file 1 [file jintelligence-13-00080-s001.zip › Supplementary Folder/Stage 1 - Story Collection/Originally Collected Stories/Western Human Participants/Story 6 - Creative.pdf]

English original version

“Salmon! Get your fresh salmon!” a vendor cried, just barely loud enough to be audible over the sound of thousands of customers and tens of market stall keepers, all trying to achieve the same. His voice was rough and cracked slightly, from the strain of yelling all day. The King’s Lane was busy as ever, perhaps even moreso. Rahira hastily made her way through the crowd. Her movements were methodical, as she weaved in and out of open spaces in the large mass of people. Apple stands she passed, and bread vendors. Butchers, too. The street was the stage to a cacophony of sales pitches, haggling and the clinking of coin. Further ahead, Rahira heard the trumpets call. She quickened her pace even more, wriggling herself through small cracks between hands, legs, and elbows. She even got struck by a few, but she didn’t have time to pay attention to that. Again, the trumpets sounded. They were at least twenty, no, fifty. She jumped from side to side, making her way to the walls, doors and windows of the houses that lined the Lane, then suddenly strafing back into the crowd. A type of traversal one can only learn from living in a city as big as the Capital, and then still you would have to study the dance of the crowds for years. She was sure she was going to miss it. All days, she was up early, but on the day of her father’s departure to the war in the far west, she overslept. Sleep hadn’t embraced her until the sun was already risen. She had been too worried. Too scared. She leapt, through the crowds, just barely making it to a full sprint. She could already feel the breeze of the sea, and she could smell the docks’ taverns by their ale, and the warehouses by their salted meats. The crowd was thinning, as market reached its end. Only true, hardy hagglers and clever salesmen were left to fill the street. Finally freeing herself from the masses, she sprinted the last bit of the way down the slope to the sea. Just ahead, the orderly, imposing formations of soldiers were marching in perfect synchrony toward the large transport ships that were docked at the pier. She finally caught up with them, and trailed past the leagues of soldiers, looking for her father. And there he was. Upon a black steed, surrounded by bannermen, her father rode in the parade. Trumpets called again. Rahira called out to him. They were near deafening up close. Despite the ringing in her ears, she called out again. This time, Rahira’s father turned his head, and saw her. She smiled a full smile, but not one of joy or contentness. It was a smile of reassurance and wisdom. He knew what was about to come to pass. She knew it too. Deployment was planned to last five years. The letter from the general’s scribe had clearly stated it. Five years, if the legion would hold out that long. Distress calls from the front had only been increasing in number and frequency. Finally, at the end of the King’s Lane, the soldiers were called to a halt. For some time they stood still. Then, they turned and moved to the large military dining hall. One more meal on the soil of home. One more night of song, dance, and joy. Rahira’s father looked at her one more time. He summoned the same smile to his face, but this one was more. This one said “love”. Rahira’s tears said the same. Five years.

Chinese translation

“三文鱼！新鲜的三文鱼！”一个小贩叫喊着，声音勉强足够大，能在成千上万的顾客和十几个市场摊位主之间听到，他们都在努力做同样的事情。他的声音沙哑，稍微有些嘶哑，因为整天喊叫的劳累。国王巷一如既往地繁忙，甚至可能更加繁忙。拉希拉匆忙地穿过人群。她的动作是有条理的，她在人群的大群人中穿梭，来回穿梭。她路过了苹果摊，面包摊。还有屠夫。街道上充满了各种吆喝声、讨价还价声和硬币的叮当声。在更远的前方，拉希拉听到了号角声。她加快了步伐，穿过人们之间的小缝隙，手、腿和肘部之间。她甚至被一些人碰到了，但她没有时间去注意这些。再次，号角声响起。至少有二十个，不，五十个。她左右跳跃，穿过巷道两侧的墙壁、门和窗户，然后突然又钻进人群中。这种穿梭方式只有在如首都这样大的城市中

生活过，然后你仍然需要多年的时间研究人群的舞蹈。她确信自己要错过了。每天，她都起得很早，但在她父亲远征西部的那一天，她睡过头了。直到太阳升起才入睡。她太担心了。太害怕了。她跃过人群，勉强达到全速奔跑。她已经能感觉到海风，还能通过他们的啤酒闻到码头的酒馆和肉库的咸肉。随着市场接近尾声，人群开始稀疏了。只有真正的、坚强的讨价还价者和聪明的推销员还留在街上。最终，她摆脱了人群，狂奔着最后一段路走**向海**边。就在前方，整齐有序的士兵们以完美的同步步伐向停泊在码头的大型运输船行进。她最终赶上了他们，并在士兵的队伍中寻找她的父亲。他就在那里。在一匹黑色的战马上，被旗帜围绕着，她的父亲骑在游行队伍中。号角声再次响起。拉希拉对他喊道。近距离听起来几乎令人聋。尽管耳朵里嗡嗡作响，她还是再次喊了出来。这一次，拉希拉的父亲转过头，看到了她。她露出了一个满意的微笑，但不是喜悦或满足的微笑。这是一种安慰和智慧的微笑。他知道即将发生的事情。她也知道。部署计划持续五年。将军的文书的信中明确说明了这一点。五年，如果军团能撑到那么久的话。前线的求援呼声只增不减。最后，在国王巷的尽头，士兵们被叫停了。他们站了一段时间。然后，他们转身向大型军事餐厅走去。在家乡土地上的最后一餐。最后一夜的歌声、舞蹈和欢乐。拉希拉的父亲又看了她一眼。他脸上召唤出同样的微笑，但这次更多了。这个微笑传达了**“爱”。拉希拉的眼泪也**传达了同样的情感。五年。
