# Supplementary material for: The Effects of (Dis)similarities Between the Creator and the Assessor on Assessing Creativity: A Comparison of Humans and LLMs
Source: J Intell. 2025 Jul 3;13(7):80. doi: 10.3390/jintelligence13070080 (PMC12295035; doi:10.3390/jintelligence13070080)
Supplement: Supplementary file 1 [file jintelligence-13-00080-s001.zip › Supplementary Folder/Stage 1 - Story Collection/Originally Collected Stories/Western Human Participants/Story 7 - Non-creative.pdf]

English original version

Last year Penelope went on a holiday, she was traveling all by herself for the first time. Penelope has lived towards this moment since she graduated for highschool but had to work to get the money for this big trip. When she finally had enough money, she left to the other side of the world; New Zealand. Her mother dropped her of at the airport en she flew. When she arrived at her destination, she was overwhelmed. The airport was bigger than any airport she had ever seen and she did not know where to go. At that moment she found another girl travelling alone. They were able to help each other and find a way to the luggage and out of the airport. Finally, some fresh air she thought. There were caps everywhere, people were running, walking and shouting to each other. She was in the capital city of New Zealand. It was something that she never imagined before but she could get used to it. It was inspiring to see the people do what they loved, everyone was smiling and content with their activities. It was almost as if there were no sadness. She saw people dancing on the streets, skating, making art and strangers having the most beautiful time together. She thought, why not, I will try to make contact with others as well (which was a big step for her). She walked upon a stranger and started a conversation. The conversation turned out to be interesting and took longer than expected. But Penelope actually kind of liked it. Although, she became a bit hungry. She told the stranger and he knew one of the greatest restaurants of the city. Together, they walked towards it. The stranger asked if Penelope would mind if he sat with her a little bit longer. She hesitated at first, but remembered that this trip was going to be about stepping out of her comfort zone. And while she remembered the purpose of the trip, she immediately told the stranger that she did not mind. They had a lovely lunch and he invited her to come to his work the day after. He was a volunteer at a animal hospital and wanted to show her his favorite animals. So, she came. She found out that it was a hospital for sea animals. There were dolphins all around, was one big shark and lots of other sealife. The big shark was in there because he lost a fin. He had to learn how to swim again. The hospital has been working on this project for almost 5 years and it looked like he was able to go back to the sea in a short amount of time. Afterwards Penelope went to her hotel and moved on to doing what she was planning to do. A week later, she walked into the stranger again. He told her that the shark was going to be released into the sea this afternoon. He asked her if she wanted to come as well. She immediately said yes, wow!! They left to the beach with the shark, stepped onto a boat, and once they were far enough from the shore, they released the shark. This was such a beautiful experience, all thanks to the stranger and the openness of Penelope.

Chinese translation

去年，佩内洛普去度假了，这是她第一次独自旅行。自从高中毕业以来，佩内洛普就一直在为这一刻而努力工作，以赚钱支付这次重大旅行的费用。当她终于有了足够的钱，她去了地球的另一边**——新西**兰。她的母亲送她去机场，然后她飞行了。当她抵达目的地时，她被压倒了。机场比她见过的任何机场都大，她不知道该去哪里。就在那时，她遇到了另一个独自旅行的女孩。她们能够互相帮助，并找到一条通往行李区和机场外的路。最后，新鲜空气，她想。到处都是出租车，人们在奔跑，走路，彼此大声喊叫。她在新西兰的首都。这是她以前从未想象过的事情，但她可以适应它。看到人们做自己喜欢的事情是鼓舞人心的，每个人都在微笑，对他们的活动感到满意。几乎就像没有悲伤一样。她看到人们在街上跳舞，滑冰，制作艺术品，陌生人在一起

度过了最美好的时光。她想，为什么不呢，我也会试着与其他人联系（这对她来说是一个很大的进步）。她走近一个陌生人，并开始了一段对话。对话结果很有趣，时间比预期的长。但佩内洛普其实有点喜欢。尽管，她有点饿了。她告诉陌生人，他知道这座城市最棒的餐厅之一。他们一起走向那里。陌生人问佩内洛普是否介意他再和她坐一会儿。她起初犹豫了一下，但想起这次旅行是为了走出自己的舒适区。当她想起旅行的目的时，她立刻告诉陌生人，她不介意。他们享受了美好的午餐，他邀请她第二天去他的工作地点。他是一名动物医院的志愿者，想向她展示他最喜欢的动物。所以，她来了。她发现这是一家海洋动物医院。四周都是海豚，还有一只大鲨鱼和许多其他海洋生物。这只大鲨鱼因为失去了一只鳍而在里面。它必须重新学会如何游泳。医院已经为这个项目工作了近**5年，看起来它很快就能重新回到海洋了。之后，佩内洛普去了她的酒店**，继续做她计划要做的事情。一周后，她又遇到了那个陌生人。他告诉她，今天下午鲨鱼将被释放到海里。他问她是否想一起去。她立刻说是，哇！他们带着鲨鱼去了海滩，登上了一艘小船，一旦离岸足够远，他们释放了鲨鱼。这是一个美好的经历，全靠陌生人和佩内洛普的开放性。
