# Supplementary material for: The Effects of (Dis)similarities Between the Creator and the Assessor on Assessing Creativity: A Comparison of Humans and LLMs
Source: J Intell. 2025 Jul 3;13(7):80. doi: 10.3390/jintelligence13070080 (PMC12295035; doi:10.3390/jintelligence13070080)
Supplement: Supplementary file 1 [file jintelligence-13-00080-s001.zip › Supplementary Folder/Stage 1 - Story Collection/Originally Collected Stories/Western Human Participants/Story 8 - Non-creative.pdf]

English original version

The sunlight, that is ever so slightly coming though the curtain, wakes Nathan up. It is now 8 am on a Saturday morning, way too early to wake up. But the light of the sun woke him, and the heat in the room and the noise from the street are keeping him from falling back asleep. Annoyed, he turns around while thinking to himself: ‘Ugh, this is the downside of living in LA’. Convinced he won’t fall back asleep, he grabs his phone and sees a notification of a text message. It’s from Sophia, the girl he went out with last night who is responsible for this nagging hangover. It says: ‘I had fun last night’ and the next one: ‘You wanna grab breakfast?’ He catches himself smiling and sits up in bed. ‘Sure! See you at Sunrise café in an hour?’, he replies. ‘See you then!’ reads the answer from Sophia. Nathan sweeps his leg over the edge of the bed, stands up, and walks towards the mirror. ‘My god I look horrible’, he thinks. ‘I have to hurry up if I want to look good and be there in an hour’. After a little more then an hour, he arrives at the Sunrise café. Sophia is already there, waiting on him at a table in the sun. She looks absolutely amazing. ‘Hi mister party panda!’, she calls out too him. ‘Jeez, how come you are not more hungover’, Nathan sighs. ‘That’s because I stopped drinking several shots before you did, haha’. They sit down together and order their breakfast. Nathan orders the Saturday Spirit breakfast, ideal for hangovers since it contains bagels, eggs, bacon, juice, coffee, and fries. Sophia orders the Bibabanana breakfast, with banana pancakes, juice, coffee, maple syrup, fruit, and roasted oats. They chitchat about all kinds of things and Nathan slowly feels the hangover reducing. At least he is not on the edge of puking anymore. When they are finished with their breakfast, they decide to order another coffee. After a while it starts getting too warm in the sun, which makes sense given that it is July in one of the hottest cities in America. ‘Do you want to go to the beach for a little while? Blow of sum steam?’, Sophie suggests. ‘Heck yeah’, Nathan replies. It’s a good thing that locals in LA never go anywhere without their swim clothing. While Sophie waves over the waiter, Nathan thinks to himself, ‘She might just be the most beautiful girl I have ever met.’ Even though they have only met a couple weeks ago, it feels like they were destined for each other. They decide to take the metro to the beach, since it’s way faster than the bus and also less hot. When they arrive around 12pm, the beach is already crowded with families and teenagers. They spot a group of musicians left on the boulevard and decide to watch for a while. They are playing some type of bachata music, and Nathan decides to take Sophia for a dance. After a while, Sophia says: ‘Okay, we have to stop. I’m getting too hot now’. Nathan replies: 'I thought you were pretty hot before too’. ‘Haha, you flirt. Let’s go for a swim!’, Sophia proposes. As they run of into the sea together, Nathan thinks: ‘This is the happiest I have been in a long time’.

Chinese translation

阳光透过窗帘轻轻照在了内森身上，把他唤醒了。现在是周六早上8点，早得太离谱了。但太阳的光芒唤醒了他，而房间里的热和街上的噪音又让他无法再入睡。他有些烦躁，转过身来自言自语：“唉，这就是住在洛杉矶的不好之处。”相信自己不会再入睡，他拿起手机，看到了一条短信通知。是索菲亚发来的，昨晚和他约会的女孩，也是造成他头痛欲裂的原因。短信上写着：“昨晚玩得很开心”，接着又是一条：“你想吃早餐吗？”他发现自己在微笑，坐起身来。“当然！在日出咖啡馆见面？一个小时后？”他回复道。“那时见！”索菲亚的回答如是。内森把腿从床边放了下来，站了起来，走向镜子。“天哪，我看起来真糟糕”，他想。“如果想要看起来好一点，还有一个小时就得快点了”。一小时零几分钟后，他来到了日出咖啡馆。索菲亚已经在那里了，坐在阳光下的一张桌子旁边等他。她看起来绝对美丽。“嘿，派对小熊先生！”她冲他喊

道。“天哪，你怎么没有更严重的宿醉呢”，**内森**叹了口气。“**因**为在你之前我就停止了喝了好几杯，哈哈”。**他**们一起坐下来点了早餐。内森点了周六精神早餐，对于宿醉来说是理想的，因为里面有百吉饼、鸡蛋、培根、果汁、咖啡和薯条。索菲亚点了香蕉松饼早餐，里面有香蕉松饼、果汁、咖啡、枫糖浆、水果和烤燕麦。他们闲聊着各种事情，内森慢慢感觉到了宿醉的减轻。至少他不再感到快要呕吐了。当他们吃完早餐后，决定再来一杯咖啡。过了一会儿，太阳下开始变得太热了，这在七月是有道理的，尤其是在美国最炎热的城市之一。索菲亚提议：“**你想去海**滩晒晒太阳吗？发泄一下？”“**当然**”，**内森回答道。洛杉**矶当地人离开家都会随身携带游泳衣，这一点真是太好了。当索菲亚招呼服务员的时候，内森心里想着：“**她可能是我**见过的最漂亮的女孩了。”**尽管他**们只是几周前才认识，但感觉他们注定要在一起。他们决定乘地铁去海滩，因为比公交车快得多，也不那么热。当他们在中午左右到达时，海滩已经挤满了家庭和青少年。他们看到大道上还留着一群音乐家，决定停下来看一会儿。他们在演奏一种巴恰塔音乐，内森决定带索菲亚跳一支舞。过了一会儿，索菲亚说：“**好了，我**们得停下来了。我现在太热了。”**内森回答道**：“**我**觉得你之前也很火辣。”“**哈哈，你在**调情。我们去游泳吧！”**索菲**亚建议道。当他们一起跑向大海时，内森心想：“这是我很久以来最幸福的时刻”。
