# Supplementary material for: The Effects of (Dis)similarities Between the Creator and the Assessor on Assessing Creativity: A Comparison of Humans and LLMs
Source: J Intell. 2025 Jul 3;13(7):80. doi: 10.3390/jintelligence13070080 (PMC12295035; doi:10.3390/jintelligence13070080)
Supplement: Supplementary file 1 [file jintelligence-13-00080-s001.zip › Supplementary Folder/Stage 1 - Story Collection/Originally Collected Stories/Western Human Participants/Story 9 - Non-creative.pdf]

English original version

My first day of free time out here in Barcelona. I have been looking forward to this day from the moment I started my new job. I needed a job quick, so I looked everywhere to find one. However, this job is not the best. To be honest, my colleagues make it bearable. My boss on the other hand is a total disaster. I get the feeling he does not like me at all. I correct myself: I do not have to worry today about work, bosses or colleagues. It is my first free day in three weeks! I moved to the city a month ago and have not seen very much of it. The weather is very nice today, hence why the streets are so busy. I wonder what I would like to do now. I think a park would be a nice start of this day. A few moments later I arrive at park Güell and lay down my blanket on the green grass. I sit down and take my book out of my bag. I start reading and time flies by. Hours later I come to my senses by my stomach making a growling sound. I now realize I have not eaten a single thing today, but only grabbed a coffee on the way here. While I am packing my stuff, I see a squirrel running by. It reminds me of my elderly home, back in the country, where I saw squirrels in the backyard every day. My father sometimes fed them nuts directly out of his hand. I smile as I walk my way to the exit of the park. Finally, I sit down at a café, to eat something. The waiter comes over and I see the best pancakes I have ever seen coming my way. I am drooling. I quickly grab my phone to snap a picture before I demolish the pancakes. They were so expensive, but it is worth all my money. One second, I think about getting a second plate, but I decide not to and head towards the beach. The waves splash onto shore, and I feel so at ease. Here at the beach with the sun burning on my face, it feels like I have no problems at all. Maybe I will take a dip in the ocean later. First, I am drowning myself in sunscreen and put on my big hat. I lay down in this chair I rented. I have the best view over the beach and can look very far. In my free time, I love doing some people watching, and what better place than the beach? There are lots of families. I see fathers building sandcastles with their children, and mothers taking their babies to feel the cold sea water for the first time. I must have taken a nap, because when I wake up the beach is not full anymore. There are a few people left, groups of friends who are drinking beers and relaxing after a day of studying. I look to my left and see: a squirrel. It is the same as I saw this morning because I recognize the unusual red spot on his forehead.

Chinese translation

我在巴塞罗那度过的第一个自由时间。从我开始新工作的那一刻起，我就一直期待着这一天。我需要尽快找到一份工作，所以到处都在找。然而，这份工作并不是最好的。老实说，是我的同事们让它变得能忍受。但是我的老板简直是个灾难。我觉得他根本不喜欢我。我要自我纠正：今天我不用担心工作、老板或同事。这是我三周来的第一个休息日！我一个月前搬到这个城市，但还没有看到太多。今天天气很好，所以街上非常繁忙。我在想现在想做什么。我觉得去公园是这一天的一个不错的开始。几分钟后，我来到了格韦尔公园，将毯子铺在绿草地上。我坐下来，从包里拿出了书。我开始阅读，时间飞逝。几个小时后，我的胃开始咕咕叫。我意识到我今天一整天都没有吃东西，只是在来这里的路上买了杯咖啡。当我收拾东西时，我看到一只松鼠跑过。这让我想起了我在乡下的老家，每天都能在后院看到松鼠。我父亲有时会直接从手中喂它们坚果。我笑着走向公园的出口。最后，我坐在一家咖啡馆里吃东西。服务员过来了，我看到了我见过的最棒的煎饼就要送来了。我在流口水。我赶紧拿起手机拍了张照片，然后就要毁掉这些煎饼了。它们太贵了，但是花了我所有的钱也是值得的。我想着要不要再点一个盘子，但我决定不要，转身朝海滩走去。海浪拍打着岸边，我感到非常轻松。在这里，海滩上

阳光晒在脸上，感觉就像我根本没有任何问题一样。也许我晚些时候会在海里游个泳。首先，我在自己身上涂抹了防晒霜，戴上了我的大帽子。我躺在我租的椅子上。我有最好的海滩景色，可以看得很远。在空闲时间，我喜欢观察人群，哪里比海滩更好呢？有很多家庭。我看到父亲和孩子们一起筑沙堡，母亲带着婴儿第一次感受冰冷的海水。我一定是睡着了，因为当我醒来时，海滩上不再那么拥挤了。还有一些人，是一群朋友，他们在喝啤酒，放松一天的学习。我向左看去，看到了一只松鼠。这和今天早上看到的一样，因为我认出了它额头上的异常红斑
