# Supplementary material for: The Effects of (Dis)similarities Between the Creator and the Assessor on Assessing Creativity: A Comparison of Humans and LLMs
Source: J Intell. 2025 Jul 3;13(7):80. doi: 10.3390/jintelligence13070080 (PMC12295035; doi:10.3390/jintelligence13070080)
Supplement: Supplementary file 1 [file jintelligence-13-00080-s001.zip › Supplementary Folder/Stage 1 - Story Collection/Testing Instructions for Stage 1.pdf]

Testing Instructions for Stage 1 (English)

[Pretend you are a university student. / Pretend you are a university student who has been given this task.]

Your Task

We ask you to write [a common, not very creative / an uncommon, very creative] story of 500-600 words. To help you a bit we will give you three small details that we like you to include in your story. The first detail should be included somewhere in the beginning of your story. The second detail somewhere in the middle. And the third detail somewhere at the end. Other than these three details you are completely free to write about anything. However, please do not write any offensive messages in your story, and please do not share any personal information of your own in the story you write.

Given Details

Please include somewhere in the beginning of your story the following detail: Something related to a busy street in a big city. Please include somewhere in the middle of your story the following detail: Something related to a tasty meal. Please include somewhere at the end of your story the following detail: Something related to the sea.

(The following note was only added to human participants)

We recommend you to:

- Finish the story in about 30-60 minutes.
- Write the story offline (e.g., in Word) to prevent unexpected loss because of the network breaking down.

Testing Instructions for Stage 1 (Chinese)

[假设你是一名大学学生]

**您的任务

在这项实验中，请写下一个500至600字的非创新、平凡[或者创新、不平凡]的故事。我们将提供三个小细节，希望您能将它们加在您的故事中。这三个细节分别需要出现在：故事的开头，故事的中间和故事的结尾。除了这三个细节以外，您可完全自由发挥任何内容。但是，请不要在您的故事中包含任何具有冒犯性的信息，也不要分享有关您自己的任何个人信息。

细节信息

请在故事开头的某个位置包含以下详细信息：
与大城市繁忙街道有关

请在故事中间的某个位置包含以下详细信息：
与好吃的食物有关

请在故事结尾处包含以下详细信息：
与海洋有关

请在下面的文本框中写下您的故事。

我们建议您：

- 在30-60分钟内完成该故事。
- 线下撰写您的故事（如在Word中），以防止因网络断联而导致丢失。
