# Supplementary material for: The Effects of (Dis)similarities Between the Creator and the Assessor on Assessing Creativity: A Comparison of Humans and LLMs
Source: J Intell. 2025 Jul 3;13(7):80. doi: 10.3390/jintelligence13070080 (PMC12295035; doi:10.3390/jintelligence13070080)
Supplement: Supplementary file 1 [file jintelligence-13-00080-s001.zip › Supplementary Folder/Stage 2 - Story Assessment/Testing Instructions for Stage 2.pdf]

Testing Instructions for Stage 2 (English)

[Pretend you are a university student. /Pretend you are a university student who has been given this task.]

Your Task

In this experiment, you will read a total of 16 short stories with a length of approximately 400-600 words. After reading each story, we kindly ask you to rate how creative this story is with three questions. Please read the explanations of each question carefully before choosing a score.

[Story]

Intuitively, how creative is this story?
1: Not creative at all
2
3
4
5
6: Very creative

Researchers generally agree that, for something to be considered creative, it should be original (novel, unique, or surprising) AND appropriate (valuable, effective, or aesthetically appealing).

Specifically:

- **Originality** refers to an idea or product being unique from other ideas or products, typically with a lower probability of occurring among the general population, and most people would not think of it.
- **Appropriateness** refers to an idea or product being appropriate and valuable in the specific context wherein it is generated, typically (with some imagination) reasonable or realizable.

Based on the above elaborations, how original is this story?
1: Not original at all
2
3
4
5
6: Very original

Based on the above elaborations, how appropriate is this story?
1: Not appropriate at all
2
3
4
5
6: Very appropriate

Testing Instructions for Stage 2 (Chinese)

[假设你是一名大学学生]

欢迎参加本次实验！

您的任务

在本实验中，您将一共阅读并评估 16 个字数约500-600字的短篇故事。对于每个故事，我们将会问您三个问题，分别涉及到故事的原创性、恰当性和创意力。

请从 1 至 6 分进行打分，1 表示最低，6 表示最高。

*** 请仔细阅读每个问题下方关于术语含义的解释。***

[故事#]

Q1. 不假思索的话，您觉得这个故事的创造性如何？

1 完全没创造性
2
3
4
5
6很有创造性

研究人员普遍认为，认可某些事物具有创造性应同时考虑，它是否是独创的（新颖、独特或令人惊奇）并且适恰的（有价值、有效或具有审美吸引力）。

- 独创性通常指一个想法或产品独特于其他想法和产品存在，通常在人群中产生的概率比较小，即大部分人都想不到。
- 适恰性通常指一个想法或产品在被提出的场景下是合适的、有价值的，存在一定的合理性（可能需要一定程度的想象）或可实现性。

Q2. 基于上面的解释，您觉得这个故事的独创性如何？

1 完全不独创
2
3
4
5
6非常独创

Q3. 基于上面的解释，您觉得这个故事的适恰性如何？

1完全不适恰

2

3

4

5

6非常适恰
